# Higher-Order Thinking Skills Optimizer: A Metaheuristic Algorithm Inspired by Human Behavior and Its Application in Real-World Constrained Engineering Optimization Problems

**DOI:** 10.3390/biomimetics11030191

**Published:** 2026-03-05

**Authors:** Zhixin Han, Ying Qiao, Hongxin Fu, Yuelin Gao

**Affiliations:** Ningxia Collaborative Innovation Center for Scientific Computing and Intelligent Information Processing, School of Mathematics and Information Science, North Minzu University, Yinchuan 750021, China; 15620696128@163.com (Z.H.); 15530163089@163.com (H.F.); 1993001@nun.edu.cn (Y.G.)

**Keywords:** human-inspired algorithm, metaheuristic, constrained problems, optimization algorithm, higher-order thinking skills, engineering design

## Abstract

With the increasing complexity of optimization problems, existing methods are often inadequate for addressing these challenges, creating a pressing need for more versatile and robust approaches capable of solving a wide range of optimization problems. Meta-heuristic algorithms have become powerful tools in this regard, owing to their flexibility, ease of implementation, and suitability for high-dimensional and complex problems. This paper introduces the Higher-order Thinking Skills Optimizer (HTSO), a novel meta-heuristic algorithm inspired by Higher-order Thinking Skills (HOTS) from educational theory. HTSO simulates the four key aspects of HOTS: creativity, problem-solving, critical thinking, and decision-making. Creativity reflects the intrinsic human drive for knowledge, prompting exploration of unknown domains. When faced with difficulties, individuals focus on gathering information to solve problems. However, due to the inconsistent quality of information, critical thinking is essential for effectively assessing it. In HTSO, creativity and problem-solving serve as the exploration and exploitation mechanisms, respectively. Crucially, critical thinking functions as a metacognitive controller that evaluates the quality of solutions and dynamically guides the selection and adaptation of creativity strategies, thereby ensuring an effective balance between exploration and exploitation. Moreover, HTSO is designed as a user-friendly algorithm with minimal parameter tuning requirements, and its key parameter demonstrates strong robustness across diverse problem types and dimensions, which enhances its practical applicability. Extensive experiments were conducted across three CEC benchmark sets with multiple dimensions (CEC-2017: 30, 50, 100 dimensions; CEC-2020: 10, 15, 20 dimensions; CEC-2022: 10, 20 dimensions), comparing HTSO with 21 other algorithms, including several CEC champion algorithms. The results demonstrate that HTSO outperforms all comparative algorithms on most test functions, indicating high effectiveness and robustness. Furthermore, HTSO was compared with 14 algorithms on 12 real-world constrained engineering optimization problems. Finally, HTSO and 14 other algorithms were applied to unmanned aerial vehicle 3D path planning in seven different complex mountainous scenarios. HTSO also achieved the best performance across all tested real-world engineering problems and UAV path planning scenarios, consistently outperforming the comparative algorithms. These results demonstrate the effectiveness and potential of HTSO in addressing real-world optimization challenges.

## 1. Introduction

An optimization problem involves finding the optimal solution that either maximizes or minimizes an objective function, subject to a given set of constraints [1]. In practical engineering applications, the first step is to define the optimization objective—such as minimizing mass, cost, or time—and identify the independent variables that influence this objective. A mathematical model for the objective function is then developed based on the relationship between these variables and the objective. Constraints, such as time, resource, and physical limitations, are expressed as mathematical equations or inequalities. By integrating the objective function with these constraints, a complete optimization model is constructed. A single-objective optimization model can be represented by Equation (1).

(1) $$\begin{array}{cc} \; & Minimize:\;f\left(\vec{x}\right),\vec{x}=\left(x_{1},x_{2},\ldots ,x_{D}\right)\\ \; & Subject\;to:\\ \; & g_{i}\left(\vec{x}\right)\leq 0,i=1,\ldots ,n\\ \; & h_{j}\left(\vec{x}\right)=0,j=n+1,\ldots ,m\\ \; & lb_{u}\leq x_{u}\leq ub_{u},u\in 1,2,3,\ldots ,D \end{array}$$

In this context, $f(\vec{x})$ denotes the objective function, and $\vec{x}$ is a *D*-dimensional vector of variables, $x_{1}$ through $x_{D}$. The functions $g_{i}\left(\vec{x}\right)$ and $h_{j}\left(\vec{x}\right)$ represent *n* inequality constraints and *m* equality constraints, respectively. The variables $lb_{u}$ and $ub_{u}$ indicate the lower and upper bounds of $x_{u}$, respectively.

Over time, researchers have developed numerous optimization methods to tackle a diverse set of problems. In recent years, rapid technological advancements and the emergence of artificial intelligence (AI) have helped alleviate resource scarcity caused by a growing population. However, these developments have also led to increasingly complex optimization challenges. Consequently, traditional optimization algorithms often fail to effectively solve all problem types. As a result, identifying a versatile algorithm capable of addressing a broad range of optimization problems remains a critical focus of current research.

Existing optimization methods are generally divided into two main categories: deterministic and stochastic approaches [2,3]. Deterministic methods include well-established techniques such as linear programming [4], dynamic programming [5], gradient descent [6], and Newton’s method [7]. These methods offer advantages like stability, controllability, and strong theoretical support. However, they often encounter significant challenges when addressing complex problems, including high computational demands and a tendency to become trapped in local optima. Moreover, the search space of real-world constrained optimization problems is highly complex and irregular. High-dimensional problems are often associated with a nonlinear and nonconvex nature. Traditional deterministic methods, limited by theoretical constraints, are generally effective only for solving simple problems in theory and are frequently inadequate when faced with complex optimization tasks. Consequently, deterministic methods are increasingly inadequate for solving today’s complex optimization tasks. In contrast, stochastic methods have demonstrated notable progress in terms of stability, robustness, and generalizability, making them powerful tools for tackling complex optimization problems.

With the advancement of AI and the progression of related research, a highly efficient optimization approach—meta-heuristic algorithms—has emerged [8]. These algorithms utilize the powerful computational capacity of computers and do not rely on problem-specific knowledge. Meta-heuristic algorithms, a subset of stochastic methods, are inspired by natural phenomena and mechanisms to solve optimization problems. These algorithms excel at avoiding local optima, thus facilitating more efficient searches for the global optimum. Owing to their flexibility, adaptability, ease of implementation, and user-friendliness, meta-heuristic algorithms have gained widespread adoption since their inception. Consequently, over the past several decades, they have been applied in a broad range of fields, including healthcare [9], finance [10], the environment [11], and image recognition [12], consistently demonstrating significant performance improvements. For instance, PSO has been utilized in typhoon wind speed prediction models [13], GWO in training multi-layer perceptrons [14], GJO in skin cancer prediction and diagnosis [15], CS in face recognition technology [16], and SA and TS in workshop scheduling problems [17], among others.

In recent years, the complexity of engineering optimization problems has increased alongside technological advancements. Meta-heuristic algorithms, known for their exceptional global search capabilities and adaptability to complex optimization challenges, have demonstrated significant potential across various subfields of engineering. For instance, in aerospace, several meta-heuristic algorithms have been employed in aircraft design [18] and drone path planning [19], showcasing their versatility. GWO has proven to be highly stable in identifying optimal locations for charging station layout problems [20]. GA is applied to optimize the design of functionally graded porous nanobeams, focusing on natural frequency and buckling load. The results demonstrate substantial effectiveness, enabling the efficient identification of optimal solutions, improving performance, and aiding decision-making in the design of complex nanostructures [21]. WOA has been shown to optimize the quality of Internet of Things (IoT) services, significantly improving resource utilization, service acceptance rates, and reducing delays and energy consumption [22]. The Red Fox Optimization Algorithm efficiently designs 2D and 3D functionally graded material structures by optimizing material parameters and distributions, substantially enhancing mechanical performance through optimal material mixtures and volume fraction exponents [23]. It is evident that meta-heuristic algorithms, owing to their flexibility, adaptability, and robustness, have become increasingly important in the field of global optimization and have evolved into essential tools for addressing real-world optimization problems.

### 1.1. Classification of Meta-Heuristic Algorithms

Over the past few decades, the meta-heuristic algorithms proposed by researchers have been broadly classified into five categories: swarm-based algorithms, evolution-based algorithms, physics-based algorithms, mathematics-based algorithms, and human behavior-based algorithms. Table 1 presents the various types of meta-heuristic algorithms that have been proposed in recent decades.

- **Swarm-based algorithms** are a prominent class of meta-heuristic optimization methods inspired by the collective behaviors of social organisms such as ants, bees, birds, and fish. These algorithms employ decentralized control strategies, wherein agents coordinate their actions toward common objectives through local interactions or adaptive responses to environmental stimuli. They achieve optimization tasks through simple interactions and collaboration between individuals.
- **Evolution-based algorithms**, inspired by Darwin’s theories of natural selection and genetic evolution, emulate processes such as species evolution, survival of the fittest, genetic inheritance, and mutation, which together enhance population quality over time. They balance exploration and exploitation via mechanisms such as crossover, reproduction, and mutation, enabling the detection of high-value regions while preserving population diversity. This balance fosters rapid convergence and reduces the likelihood of premature convergence to local optima. Owing to their exceptional performance, evolution-based algorithms have been widely adopted across disciplines, including engineering and machine learning, where they have shown remarkable effectiveness in solving complex optimization problems, substantially improving both efficiency and accuracy.
- **Physics-based algorithms** are inspired by natural phenomena, laws, and mechanisms in physics, leveraging phenomena such as motion, gravity, repulsion, and energy conservation between objects to develop optimization algorithms. Guided by physical principles, they regulate the states of search agents to sustain a balanced trade-off between exploration and exploitation.
- **Mathematics-based algorithms**, inspired by mathematical formulations, leverage established formulas, functions, patterns, and other theoretical constructs to guide exploration and exploitation, thereby maintaining a balance between these two phases and enabling the population to progressively approach the theoretical optimum. As a recently developed branch of meta-heuristic algorithms, mathematics-based algorithms possess a more robust theoretical foundation than other types and exhibit substantial potential for high performance.
- **Human behavior-based algorithms** draw inspiration from human society, as well as cognitive and psychological processes, and they aim to find global optima by emulating human intelligence in collaboration, competition, and environmental adaptation. Moreover, humans, as highly intelligent agents, possess innate cognitive self-regulation that naturally balances exploration and exploitation, thereby conferring strong optimization capabilities. While such algorithms have demonstrated success on numerous problems, they often model relatively macroscopic or monolithic social learning processes. There remains a significant opportunity for further research in finely modeling the cognitive differences within individuals, their dynamic decision-making processes, and strategy adjustments based on self-assessment.

### 1.2. Exploration and Exploitation

Exploration and exploitation are two fundamental components of meta-heuristic algorithms. Exploration enables the algorithm to investigate the solution space extensively, whereas exploitation concentrates on refining the search within areas deemed promising, as discovered through exploration, in order to move closer to the global optimum. Meta-heuristic algorithms typically commence their search for an optimal solution within the problem’s solution space using an initial matrix of randomly generated solutions. During the iterative process, these random solutions interact, exhibiting behavior that fluctuates between dispersal and aggregation, which drives their continuous movement within the solution space. Typically, in the initial stages of iteration, the entire population is dedicated to discovering high-potential regions to prevent premature convergence to a local optimum. Once the search reaches a mature stage, and it is deemed that high-potential regions have been sufficiently explored, the algorithm’s primary focus gradually shifts from exploration to exploitation, enabling the population to efficiently refine its search within these promising areas.

Maintaining an effective balance between these two processes is crucial to avoiding premature convergence to local optima and increasing the likelihood of identifying the global optimum. Furthermore, achieving this balance enhances the algorithm’s adaptability and robustness when addressing a wide range of optimization problems. Equations (2) and (3) provide the definitions for the metrics that assess the exploration and exploitation capabilities of meta-heuristic algorithms. Particle diversity, which provides additional insight into search behavior, is measured using Equation (4). Notably, recent studies have observed that optimal algorithm performance is often achieved when the exploration and exploitation curves intersect at approximately 10% of the maximum number of iterations [107].

(2) $$Exploration\left(\%\right)=\frac{Di\nu (t)}{Di\nu_{max}}\times 100$$

(3) $$Exploitation\left(\%\right)=\frac{\left|Div\left(t\right)-Div_{max}\right|}{Div_{max}}\times 100$$

(4) $$Div\left(t\right)=\frac{1}{D}\sum_{d=1}^{D}\frac{1}{N}\sum_{i=1}^{N}\left|median\left(x_{d}\left(t\right)\right)-x_{id}\left(t\right)\right|$$

Overall, the performance of meta-heuristic algorithms in addressing complex, real-world optimization problems is significantly dictated by the strategic balance and management of their exploration and exploitation phases.

### 1.3. Motivation

Although meta-heuristic algorithms have made significant progress over the past several decades, some issues remain unresolved. For instance, as discussed in Section 1.2, while the optimal balance between exploration and exploitation can maximize algorithm performance, how to achieve this balance remains a challenge. Recent studies have shown that dynamically adjusting parameters and integrating other optimization techniques can effectively balance exploration and exploitation; however, these approaches are not universally applicable and therefore require further improvement [108].

Moreover, as meta-heuristic algorithms function as black-box models characterized by complex internal mechanisms and extensive use of randomness, their interpretability is limited. This makes it difficult to theoretically explain the effectiveness and stability of the employed strategies, which is a major barrier to their adoption as effective tools for solving critical real-world problems [109]. Therefore, enhancing interpretability without compromising algorithm performance represents a significant current challenge.

To address these issues, researchers continuously propose novel update strategies and new algorithms, striving for breakthroughs in effectiveness and applicability. While an increasing number of engineering problems are being addressed using meta-heuristic algorithms, the ’No Free Lunch’ theorem [110] asserts that no single algorithm is universally applicable. Therefore, the pursuit of more robust and widely applicable meta-heuristic algorithms remains a central goal in the field.

Although human behavior-based algorithms, particularly Teaching–Learning-Based Optimization (TLBO) and Brain Storm Optimization (BSO), have garnered significant attention for their intuitive concepts, they exhibit certain inherent limitations in their algorithmic mechanisms. For instance, TLBO simplifies the population into a single teacher and a group of learners, where all learners follow similar patterns of learning from the teacher or each other. This homogeneous learning strategy overlooks the differences in cognitive levels among individuals, potentially causing the population to converge prematurely to the current best solution and become trapped in local optima. Similarly, while BSO uses clustering to simulate idea generation, its strategy selection is relatively fixed and lacks a self-adaptive mechanism for dynamically adjusting the aggressiveness of its exploratory behavior based on the quality of an individual’s solution. While metaphorically successful, these algorithms lack, at an algorithmic level, the core human ability to reflect on one’s own state and choose different problem-solving pathways.

To address these limitations, this paper proposes a novel meta-heuristic algorithm, the Higher-order Thinking Skills Optimizer (HTSO), inspired by Higher-order Thinking Skills (HOTS) from educational psychology. The core innovation of HTSO is not merely to simulate a human behavior, but to construct, at the algorithmic level, a dynamic multi-strategy search framework based on individual self-assessment. Specifically, HTSO introduces a critical thinking module that enables each individual to evaluate the quality of its own solution. Based on this assessment, an individual adaptively selects one of three distinct creativity strategies for exploration: leaders adopt a robust Expert’s Breakthrough, mid-level individuals engage in Collaborative Exploration, and under-performers execute a large-step Paradigm Shift. This heterogeneous exploration mechanism allows the algorithm to intelligently balance global exploration and local exploitation according to the search progress and individual states.

Furthermore, HTSO performs fine-grained local search by simulating problem-solving combined with Lévy flight and ensures the population’s evolutionary direction through a decision-making stage. Compared to existing algorithms, HTSO’s contribution lies in translating abstract cognitive processes into a concrete, adaptive operator selection mechanism, aiming to provide a more powerful and robust optimization tool for tackling increasingly complex modern optimization problems.

### 1.4. Contribution

Inspired by the theory of Higher-order Thinking Skills (HOTS) in educational psychology [111], this work proposes a novel meta-heuristic algorithm and underscores the following major contributions:

- Inspired by Higher-order Thinking Skills (HOTS), we propose a novel meta-heuristic algorithm, the Higher-order Thinking Skills Optimizer (HTSO). Its core contribution lies in introducing a self-assessment-driven, dynamic multi-strategy search mechanism. The algorithm simulates critical thinking to evaluate the quality of each individual’s solution and, based on the outcome, adaptively selects one of three distinct creativity update strategies. This enables heterogeneous exploration behaviors, overcoming the limitations of monolithic learning strategies found in conventional human-based algorithms, thereby achieving a more effective balance between global exploration and local exploitation.
- In this study, the effectiveness of HTSO and 21 other algorithms is systematically examined using three benchmark suites: the CEC-2017 set with problem sizes of 30, 50, and 100; the CEC-2020 set featuring 10, 15, and 20 dimensions; and the CEC-2022 set comprising 10 and 20 dimensions. These algorithms include award-winning methods, such as DE, LSHADE, LSHADE-cnEpSin, and LSHADE-SPACMA, which have received recognition in CEC competitions. To determine if the observed differences among algorithms are statistically meaningful, the Wilcoxon rank-sum test is utilized. Algorithm stability is depicted using boxplots, and convergence speed is represented by convergence curves. Finally, the experimental results are visualized with Sankey diagrams, stacked bar charts, heatmaps, and Friedman ranking line charts. The results indicate that HTSO outperforms the comparison algorithms on the majority of test functions across all dimensionalities, demonstrating its high effectiveness and robustness.
- Fifteen algorithms, including HTSO, are evaluated on 12 real-world constrained optimization problems in the field of mechanics. The results are presented through boxplots, convergence curves, and radar charts, which depict the rankings of the algorithms based on both their average and best performance across the problems.
- HTSO is applied to the unmanned aerial vehicle (UAV) 3D path planning problem and evaluated against 14 other algorithms on seven distinct mountain terrains. The results show that HTSO outperforms all other algorithms in terms of overall performance.

Due to the large volume of experimental data from the CEC test set, presenting all results within the main text would occupy excessive space. Therefore, the detailed results of the CEC test set experiments are provided in Table S1 while the results of the Wilcoxon rank-sum tests are included in Table S2. The minimum value in each row of Table S1 is highlighted in bold.

The remainder of this paper is organized as follows: Section 2 introduces the main concepts of HOTS in education and discusses how HOTS inspired the development of HTSO. Section 3 provides a detailed explanation of the HTSO algorithm. Section 4 presents the experimental results of HTSO on three benchmark test suites: CEC-2017 (30, 50, and 100 dimensions), CEC-2020 (10, 15, and 20 dimensions), and CEC-2022 (10 and 20 dimensions). Section 5 evaluates the generalizability of HTSO to real-world engineering problems through experiments on 12 mechanics-related constrained engineering optimization problems. Section 6 discusses the experimental results of applying HTSO to UAV 3D path planning across seven mountainous terrain models. Finally, Section 7 presents the experimental conclusions, highlights the strengths and limitations of HTSO, and suggests directions for future work.

## 2. Inspiration and Foundations of Higher-Order Thinking Skills Optimizer

This section will provide a detailed explanation of the inspiration behind HTSO—Higher-order Thinking Skills (HOTS)—and briefly describe how we simulate HOTS to construct the mathematical model of HTSO.

### 2.1. HOTS History

Higher-order Thinking Skills (HOTS) refer to cognitive processes that go beyond the simple recall of facts or basic comprehension. These skills involve the ability to analyze, evaluate, synthesize, and create new knowledge, enabling individuals to solve complex problems, make reasoned decisions, and think critically and creatively. In the development of education in the 21st century, HOTS are widely recognized as core abilities for enhancing learning outcomes, fostering creativity, and solving complex problems [112]. Unlike lower-order thinking skills (such as rote memorization), HOTS emphasize understanding, analysis, evaluation, and innovation. Its fundamental components typically include critical thinking, creativity, problem-solving abilities and decision-making thinking [113]. This concept was first proposed by Bloom’s Taxonomy of Cognitive Domains [114] and has undergone theoretical expansion and practical deepening from the late 20th century to the early 21st century, gradually becoming an important standard for measuring students’ deep learning abilities in higher education.

Recent advancements in educational methodologies, including Blended Learning (BL) [115] and Collaborative Inquiry-Based Learning (CIBL) [116], have led to a growing body of research on Higher-order Thinking Skills (HOTS). For instance, Lee et al. (2024) [117] argued that within the Self-Regulated Learning (SRL) framework, guiding students to generate their own ideas and subsequently employing technological tools for iterative refinement can significantly enhance the development of HOTS. Additionally, Lu et al. (2021) [118] conducted empirical studies revealing that factors like learning motivation and collaborative communication positively influence HOTS through the “deep learning pathway,” confirming the mediating role of “learning methods” on HOTS within CIBL environments. This suggests that HOTS are developed through iterative processes of cognitive processing, collaboration, and evaluation, a paradigm that lends itself well to the structure of an optimization algorithm.

Although HOTS have been extensively utilized in education—particularly in the development of teaching strategies, classroom activity design, and learning outcome assessment—it is notable that their core principles have not yet been systematically incorporated into the design of meta-heuristic algorithms within the field of optimization. Notably, the four fundamental components constitute the essential capabilities required for solving complex optimization problems. Building on this insight, the present study introduces a novel meta-heuristic optimization algorithm, termed Higher-order Thinking Skills Optimizer (HTSO), which draws inspiration from the core elements of HOTS.

### 2.2. HOTS Core

The four core components of HOTS—creativity, problem-solving, critical thinking and decision-making thinking—each play a distinct role in the learning process.

- **Creativity**: Creativity refers to the ability to combine or synthesize existing ideas, images, or expertise in novel ways, as well as the propensity to think, respond, and work imaginatively. This process is characterized by a high level of innovation, divergent thinking, and a willingness to take risks. A core component of higher-order thinking skills, it is highly valued in higher education for its role in fostering unconventional perspectives and producing original, influential ideas [119]. Promoting creative thinking enables learners to break away from traditional thought patterns, cultivating adaptable approaches to solving problems and driving innovative progress.
- **Problem-solving**: Problem-solving is the capacity to recognize and define problems, generate alternative solutions, select and implement the most effective solution, and evaluate the results. As a fundamental aspect of higher-order thinking skills, problem-solving plays a critical role in the context of higher education. It entails recognizing multifaceted challenges, collecting and evaluating relevant information, formulating viable responses, and determining the most suitable options [120]. In a constantly shifting global environment, the capacity to tackle emerging and unpredictable problems becomes essential, preparing learners to handle authentic situations and professional demands where traditional approaches may fall short.
- **Critical thinking**: Critical thinking involves actively interpreting one’s experiences and consciously expressing analytical, evaluative, and inferential judgments regarding what to believe or how to act [121]. In higher education, the ability to think critically is vital for students to assess and interpret information with objectivity, ultimately enabling sound decision-making. Such rigorous examination motivates learners to go beyond passive acceptance, prompting them to actively question and evaluate the relevance and credibility of the content they encounter [122].
- **Decision-making thinking**: Decision-making is an intellectual process that involves choosing among alternatives to respond to specific circumstances. This process is fundamental for both individuals and groups, as the quality of the choices made directly impacts subsequent outcomes. Consequently, fostering this capability in students is a vital component of education.

The primary role of HOTS is to enhance individuals’ abilities to analyze and solve problems in depth, strengthen innovation and critical thinking, and promote the development of rational decision-making and autonomous learning. By fostering higher-order thinking, individuals are better equipped to address complex challenges and adapt to societal changes, thereby demonstrating higher levels of cognition and creativity in academic, professional, and everyday contexts. Initially, creativity is employed to generate a diverse range of new ideas and solutions, which are then systematically analyzed and implemented through problem-solving skills. Subsequently, critical thinking is applied to rationally evaluate and filter these solutions, identifying their strengths and weaknesses. Finally, decision-making skills are used to weigh alternative options, make optimal choices, and execute them in practice. This process facilitates the effective integration of innovation and scientific decision-making.

### 2.3. Mapping of HOTS

In this study, we simulate four cognitive modes of HOTS: creativity, problem-solving, critical thinking, and decision-making thinking, which is the behavior that ultimately materializes and outputs the thought process. We establish a mathematical model to form the HTSO algorithm. The proposed algorithm is organized into several key stages:

- Creativity can be defined as the capacity to challenge conventions, integrate disparate information, and generate novel concepts. We translate this principle into the exploration phase of our algorithm, where individuals in the population assimilate multi-source information and traverse unconventional pathways. This process proactively investigates unexploited regions within the solution space (see Equation (9)). The core function of this phase is to emulate an inspirational leap by augmenting search diversity, thereby mitigating premature convergence. The magnitude and nature of these innovative jumps are manifested through various stochastic perturbation terms, mirroring an individual’s varied attempts in an unknown domain.
- Problem-solving involves the strategic utilization and integration of existing knowledge to perform targeted local optimization. Within the HTSO framework, this concept is analogous to the exploitation phase. During this stage, individual agents refine their positions by referencing the guidance of other agents, specifically using the current optimal individual as a benchmark for fine-tuning their immediate neighborhood (see Equation (15)). This process is comparable to human deep deduction and path refinement under established conditions, facilitating convergence toward a local optimum.
- Critical thinking is not only confined to evaluating the strengths and weaknesses of various solutions; it also serves as the metacognitive control center of higher-order thinking, guiding the direction of innovation and enabling dynamic evaluation and adaptive strategy adjustment. This faculty enables dynamic assessment and strategic self-adaptation. Within the HTSO framework, this concept is modeled by introducing a dynamic evaluation parameter, CT (see Equation (8)). CT is not merely a numerical value; rather, it simulates the thinker’s metacognitive judgment of their own cognitive level within the group. Specifically, the formula for CT directly mirrors how a learner assesses their own performance: by comparing their current state to the best-known standard within the context of the entire group’s performance range. This calculated value simulates the thinker’s metacognitive judgment of their own cognitive level within the group. A low CT value indicates that the individual perceives themselves as being at the forefront, and thus, their creative actions should be more exploratory. Conversely, a high CT value suggests that the individual recognizes a significant gap between themselves and the optimal solution, prompting them to adopt more aggressive and radical paradigm-shifting strategies.
- In the context of higher-order thinking, decision-making manifests as a goal-oriented process of dynamic adjustment and optimal selection. Within the HTSO algorithm, this cognitive concept is mathematically instantiated through a targeted selection mechanism after each evolutionary step. By eliminating inferior individuals while retaining superior choices (see Equation (18)), the algorithm operationalizes the psychological principle of real-time evaluation and rapid adjustment as a concrete mathematical procedure that promotes population convergence.

In summary, within the HTSO framework, creativity is mapped to the global exploration phase, which aims to diversify the population. Critical thinking functions as a dynamic controller that selects among various creativity strategies, optimizing the direction and effectiveness of the search. Problem-solving is mapped to the local exploitation phase, focusing the search on promising solutions. Decision-making provides the selection mechanism that drives the population towards optimal solutions.

## 3. Higher-Order Thinking Skills Optimizer

**Figure 1:**
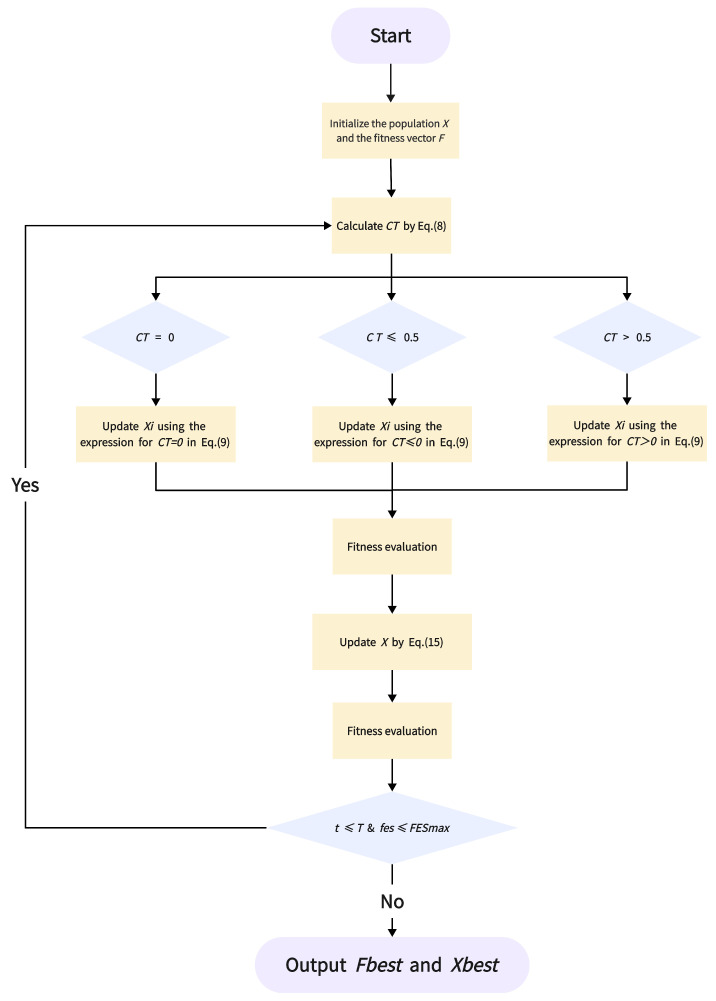
The flowchart of HTSO.

Section 2 introduced the inspiration behind HTSO. This section (Section 3.1, Section 3.2, Section 3.3 and Section 3.4) provides a detailed description of the mathematical models developed based on HOTS’s four cognitive abilities, which together form the complete HTSO algorithm. Section 3.5 presents the time complexity analysis of HTSO, followed by its pseudocode. The flowchart of HTSO is shown in Figure 1.

### 3.1. Initialization Phase

HTSO is a meta-heuristic algorithm that begins by initializing a population of *N* search agents. Each agent is randomly generated based on Equation (5).

(5) $$X_{i,j}=lb_{j}+r_{0}\times \left(ub_{j}-lb_{j}\right),i=1,2,\ldots ,N,j=1,2,\ldots ,Dim$$

(6) $$X={\left[\begin{array}{cccccc} x_{1,1} & x_{1,2} & \ldots & x_{1,j} & \ldots & x_{1,Dim}\\ x_{2,1} & x_{2,2} & \ldots & x_{2,j} & \ldots & x_{2,Dim}\\ \vdots & \vdots & \ddots & \vdots & \ddots & \vdots \\ x_{i,1} & x_{i,2} & \ldots & x_{i,j} & \ldots & x_{i,Dim}\\ \vdots & \vdots & \ddots & \vdots & \ddots & \vdots \\ x_{N,1} & x_{N,2} & \ldots & x_{N,j} & \ldots & x_{N,Dim} \end{array}\right]}_{N\times Dim}$$

*X* represents the population matrix, where $X_{i}$ denotes the $i^{th}$ individual in the population, and $X_{i,j}$ indicates the value of the $i^{th}$ individual in the $j^{th}$ dimension. Dim refers to the problem’s dimensionality. The variables lb and ub represent the lower and upper bounds of the solution space, respectively, while $r_{0}$ denotes a random number uniformly distributed between 0 and 1. The initialized population is expressed in Equation (6). The vector *F*, consisting of the fitness values obtained by evaluating the objective function for each individual, is referred to as the fitness vector, as shown in Equation (7).

(7) $$F={\left[\begin{array}{c} F_{1}\\ \vdots \\ F_{1}\\ \vdots \\ F_{N} \end{array}\right]}_{N\times 1}={\left[\begin{array}{c} F(X_{1})\\ \vdots \\ F(X_{i})\\ \vdots \\ F(X_{N}) \end{array}\right]}_{N\times 1}$$

where $F_{i}$ denotes the objective function value calculated for the $i^{th}$ individual $X_{i}$. The HTSO algorithm assesses the quality of individuals by comparing their fitness values, progressively searching for the global optimal solution to the problem.

Subsequently, the HTSO simulates four cognitive processes in HOTS: creativity, problem-solving, critical thinking, and decision-making, while exploring novel feasible solutions in each iteration.

### 3.2. Creativity and Critical Thinking

In the HTSO algorithm, each search agent is conceptualized as an autonomous thinker. During each iteration, these agents engage in an exploratory phase, akin to a brainstorm, to generate novel solutions.

A fundamental premise, however, is that these thinkers do not share a uniform cognitive capability. During the optimization process, the assessment by individuals of their own state forms the basis for subsequent decision-making. This metacognition—defined as the evaluation and regulation of one’s own thought processes—lies at the core of critical thinking. In this study, we introduce critical thinking (CT) as a model for this metacognitive evaluation mechanism. By quantifying the metacognitive discrepancy, the algorithm can simulate the decision-making process through which individuals select different innovation strategies based on their own competency levels. Consequently, prior to generating a new potential solution during the exploration phase, each thinker $X_{i}$ first engages in self-reflection to assess the quality of its current solution. This reflective process is quantified by Equation (8).

(8) $$CT=\frac{F_{i}-F^{best}}{F^{worst}-F^{best}}$$

The mathematical structure of Equation (8) is intentionally designed to mirror the cognitive process of critical self-assessment. $F_{i}-F^{best}$ represents the cognitive discrepancy. In a learning context, a thinker critically evaluates their current understanding $F_{i}$ by comparing it against the ideal solution available $F^{best}$. This difference quantifies how far they are from the frontier of knowledge. $F^{worst}-F^{best}$ represents the overall spectrum of knowledge within the current population. It contextualizes the individual’s performance gap. A large denominator signifies a diverse group with varied performance levels, while a small one indicates a more homogeneous group. By normalizing the individual performance gap by the overall knowledge spectrum, the CT value becomes a relative, context-aware measure of self-assessed competence. It is not an absolute score but a judgment of one’s standing within the peer group—a cornerstone of critical reflection.

Therefore, the CT value is more than a normalized fitness score; it is a direct algorithmic translation of a thinker’s self-awareness. A CT value approaching 0 signifies a self-perception of being an expert at the frontier, while a value near 1 represents the self-awareness of being a novice. This self-assessment is critical, as it dictates the subsequent choice of creative strategy, moving from abstract thought to concrete action.

Based on the results of critical thinking-based self-assessment, each thinker adopts one of three distinct creative thinking modes to explore the solution space. These three modes emulate innovative approaches at various levels of expertise, ranging from expert to novice, as formally described in Equation (9). Preliminary experiments indicate that when the threshold of CT is set to 0.5, HTSO achieves a good balance between the two strategies.

(9) $$X_{i}^{new}=\left\{\begin{array}{cc} Expert^{\prime }s\;Breakthrough & CT=0\\ Collaborative\;Exploration & CT\leq 0.5\\ Paradigm\;Shift & else \end{array}\right.$$

**Figure 2:**
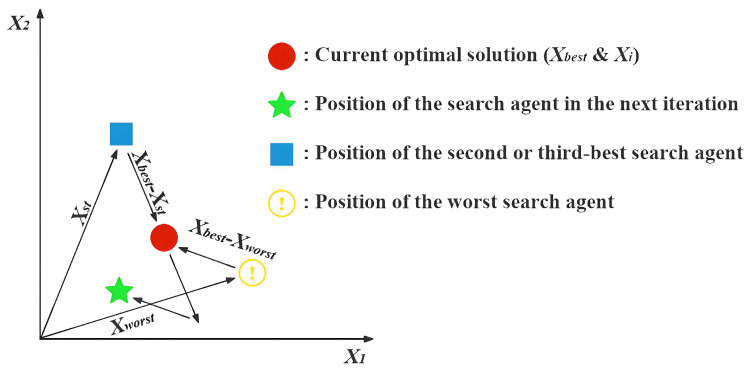
Schematic illustration of the expert’s breakthrough in two dimensions.

**Figure 3:**
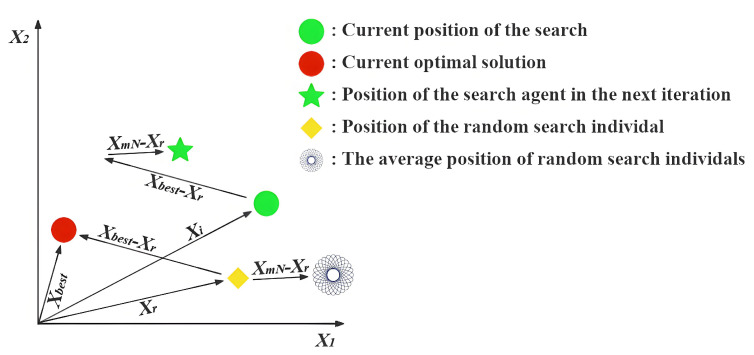
Schematic illustration of the collaborative exploration in two dimensions.

**Figure 4:**
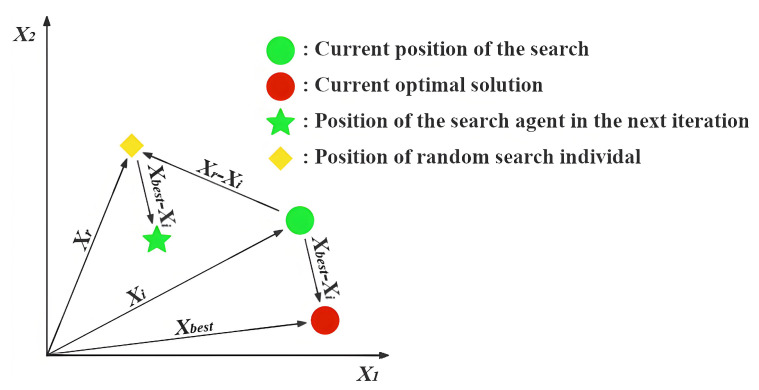
Schematic illustration of the paradigm shift in two dimensions.

- **Expert’s Breakthrough (CT=0)**: When a thinker recognizes that they are in the optimal position ($X_{i}=X^{best}$), they assume the role of a domain expert. True experts do not remain stagnant; their breakthroughs often stem from deep reflection on the existing knowledge system or by gaining insights through the analysis of failures. The mathematical modeling of this behavior is presented in Equation (10), and Figure 2 illustrates the search process under this strategy excluding the influence of random variables.
  (10) $$X_{i}^{new}=X_{i}+\left(t/T\right)\times h\left(2\right)\times \left(X^{best}-X^{st}\right)+\left(1-t/T\right)\times h\left(3\right)\times \left(X_{i}-X^{worst}\right)$$
  (11) $$h\left(\omega \right)=0.01\omega \times \sin\left(1-t/T\right)\times \vec{r}\times \;mid\times \left(\frac{3}{2}-\frac{X_{i}}{2mid}\right)$$
  (12) $$mid=\frac{ub-lb}{2}$$
  In Equation (10), $X^{st}$ denotes an individual randomly selected with equal probability between the second-ranked individual, $X^{2nd}$, and the third-ranked individual, $X^{3rd}$, based on fitness. The symbol *t* indicates the ongoing iteration, whereas *T* stands for the total allowable iterations. The expression for the function h(Ω), which incorporates random variables, is provided in Equation (11). The variable mid represents the midpoint between ub and lb, as expressed in Equation (12).
  $X_{i}$ represents the expert’s current, highly-refined knowledge base, which serves as the foundation for any new breakthrough. $X^{best}-X^{st}$ simulates an expert benchmarking against other top performers. It represents incremental innovation derived from analyzing closely related, successful ideas. $X_{i}-X^{worst}$ models the crucial cognitive act of reflecting on unsuccessful attempts to understand pitfalls and explore entirely different directions. It introduces a divergent push away from poor-performing regions. The function h(ω) is designed to simulate the spark of inspiration in an expert’s thinking process. Its components—a non-linear temporal decay sin(1−t/T) and randomness $\vec{r}$—collectively model the complex, non-deterministic nature of a creative leap.
- **Collaborative Exploration (CT≤0.5)**: When a thinker recognizes that they are a high-quality contributor but not the best performer, they adopt an open and collaborative learning strategy. In this approach, the thinker not only learns from the leader but also draws from the collective intelligence of the team. Figure 3 illustrates the search process of this strategy when random values are omitted, and Equation (13) presents the corresponding update formula.
  (13) $$X_{i}^{new}=X_{i}+\left(\left(1-t/T\right)\times \left(X_{m_{N}}-X^{r}\right)+\left(t/T\right)\times \left(X^{best}-X^{r}\right)\right)\times r$$
  In Equation (13), $X_{m_{N}}$ is the average of 2 to *N* randomly selected $X_{i}$ values from *X*. $X^{r}$ refers to a randomly chosen $X_{i}$ from *X*, and *r* denotes a randomly generated value within the interval [0,1].
  $X^{best}-X^{r}$ reflects the process of learning from the current best thinker, embodying a preference for excellence. $X_{m_{N}}-X^{r}$ captures inspiration gained from the collective intelligence $X_{m_{N}}$, simulating the process of brainstorming and helping to prevent premature convergence to local optima. The weighting factors 1−t/T and t/T describe the shift in learning focus over time. In the early stages, the thinker gives more weight to the collective opinion (1−t/T is larger); as time progresses and the direction becomes clearer, more emphasis is placed on following the best leader (t/T increases).
- **Paradigm Shift (CT≥0.5)**: When a thinker perceives themselves as a novice or finds themselves in a suboptimal position, simple imitation or collaboration is insufficient to bring about a qualitative breakthrough. At this point, a paradigm shift or cognitive leap is required to rapidly overcome the predicament. An illustration of the search strategy is provided in Figure 4, and the corresponding formulation is presented in Equation (14).
  (14) $$X_{i}^{new}=X_{i}+100mid\times (\left(t/T\right)\times \left(X^{best}-X_{i}\right)\times \vec{N}+\left(1-t/T\right)\times \left(X^{r}-X_{i}\right)\times \vec{r_{1}}$$
  This large scaling factor 100mid represents the paradigm shift itself. Unlike the incremental steps of an expert, a novice requires a radical, large-scale change in thinking to break away from a poor position, hence the large jump size. $\left(X^{best}-X_{i}\right)\times \vec{N}$ models the primary learning strategy for a novice: strong, direct guidance from $X^{best}$. The Brownian motion vector $\vec{N}$ simulates an intense and focused, yet slightly stochastic, effort to catch up to the state-of-the-art. $\left(X^{r}-X_{i}\right)\times \vec{r_{1}}$ represents the element of random trial-and-error. While primarily following the leader, a novice also learns from random peers $X^{r}$ and makes accidental discoveries. This maintains a degree of exploration and prevents the individual from becoming a mere copy of the best solution. As with other strategies, the weights t/T shift over time, initially encouraging more random exploration and later focusing more on learning from the best as the search matures.

### 3.3. Problem-Solving

**Figure 5:**
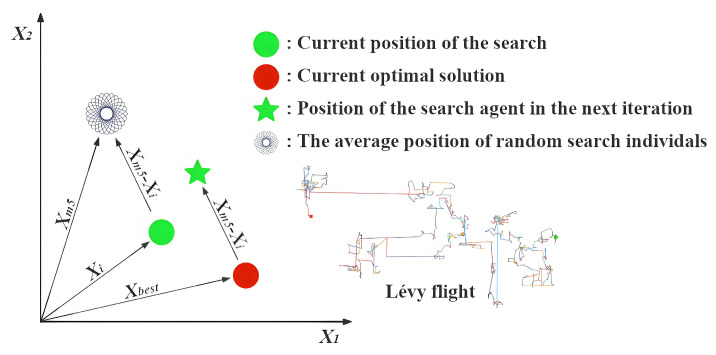
Schematic illustration of the problem solving in two dimensions.

If the creativity stage is characterized by divergent thinking and exploration, then the problem-solving stage focuses on the depth of convergent thinking, namely, exploitation. In this stage, the thinker has already identified a promising area—namely, the area around the presently optimal solution, $X^{best}$—and aims to further refine and optimize the solution. However, effective problem-solving is not merely a straightforward linear approach; it often involves both intensive, focused refinement and occasional breaks from conventional thinking patterns. To model this complex cognitive process, we introduce the mechanism of Lévy flights. Figure 5 illustrates the search process of this strategy when the random component is disregarded, and the corresponding mathematical formulation is presented in Equation (15).

(15) $$X_{i}^{new}=X^{best}+IP\times \left(X_{m_{5}}-X_{i}\right)\times ly$$

(16) $$IP={\left(1-t/T\right)}^{\left(2t/T\right)};$$

(17) $$\left\{\begin{array}{c} LF\left(y\right)=0.01\times \left(u\times \sigma \right)/(|v^{\left(1/\beta \right)}|)\\ \sigma ={\left\{\frac{\Gamma (1+\beta )\times \sin(\pi \beta /2)}{\Gamma \left(\frac{1+\beta }{2}\right)\times \beta \times \left(2^{\beta -1}\right)}\right\}}^{1/\beta } \end{array}\right.$$

In this formulation, *u* and *v* represent independent random variables uniformly distributed over the interval [0,1], and β denotes a constant, which is typically assigned the value 1.5.

In this context, $X_{m_{5}}$ denotes the average value of 2 to 5 randomly selected $X_{i}$ values from *X*, and the IP expression is provided in Equation (16). Additionally, ly represents the random vector expression formed by the Lévy flight function, as outlined in Equation (17). *u* and *v* are random variables uniformly distributed between 0 and 1, and β is a constant typically set to 1.5.

$X^{best}$ establishes the problem context. All problem-solving efforts are anchored around the current best solution, simulating a deep dive into a promising area. $\left(X_{m_{5}}-X_{i}\right)$ represents a focused peer review. The thinker refines their idea by consulting with a small, randomly selected group of peers $X_{m_{5}}$ rather than the entire population, simulating collaboration within a specialized team. The Influence Parameter IP acts as a cognitive momentum factor. Its value decreases as the search progresses t→T, meaning the influence of peer consultation diminishes over time. This models the shift from collaborative brainstorming to more independent, fine-grained tuning as a solution nears perfection. ly is the Lévy flight function. The trajectory of a Lévy flight consists of many short moves and a few long jumps. Short moves correspond to sustained, incremental improvements to a solution, which form the primary pattern of problem-solving. The long jumps, on the other hand, simulate occasional bursts of inspiration encountered during problem-solving. This allows the thinker to break out of their current cognitive set, thus avoiding becoming trapped in a comfort zone.

### 3.4. Decision-Making

In decision-making within HOTS, options frequently change dynamically, requiring continuous evaluation and timely adjustments to the decision-making strategy. Typically, we prioritize retaining those options that most closely align with the goals and provide higher value. HTSO simulates this process by defining Equation (18), enabling the optimization results to progressively approach the optimal solution.

(18) $$X_{i}=\left\{\begin{array}{c} X_{i}^{\mathrm{new}},F_{i}^{new}<F_{i}\\ X_{i},else \end{array}\right.$$

In this context, $F_{i}^{new}$ denotes the fitness value associated with the newly generated individual, whereas $F_{i}$ corresponds to the fitness measurement of the original individual.

In each iteration of the HTSO process, the population is first initialized. Next, creativity performs the exploration function, followed by decision-making, which retains the superior individuals and discards the inferior ones. The population then enters the exploitation phase (problem-solving) to improve convergence. Finally, decision-making is applied once more. This process repeats until the iteration concludes.

### 3.5. Time Complexity Analysis

The runtime required by different algorithms can vary significantly. Even the same algorithm may exhibit different execution times depending on its implementation or the computational environment in which it is executed. Therefore, algorithm performance is typically assessed by analyzing its time complexity.

In this study, we use *O* notation to represent the time complexity of the HTSO algorithm, where *T* denotes the maximum number of iterations, Dim represents the problem’s dimensionality, *N* indicates the population size, and *M* corresponds to the time complexity of evaluating the objective function.

The HTSO algorithm divides each iteration into four distinct phases: initialization, creativity, problem-solving, and decision-making.

- **Initialization phase**: The population initialization occurs only once during the entire iteration process. Given that the dimensionality is Dim and the population size is *N*, the time complexity of this step is O(N×Dim). Next, the fitness of the *N* particles must be computed, with a time complexity of O(N×M). Thus, the initialization phase has an overall time complexity given by:
  O(N×Dim)+O(N×M)=O(N(Dim+M))
- **Creativity**: As part of the exploration phase, creativity is executed once per iteration. First, the algorithm executes the critical thinking self-assessment. This step incurs negligible computational overhead as it relies solely on basic arithmetic operations using pre-evaluated fitness values, without requiring additional function evaluations. Since each particle updates its position only once during this phase, the time complexity for position updates is O(N×D). In addition, the fitness of all *N* particles must be evaluated in each iteration, resulting in a time complexity of O(N×M). Given a maximum of *T* iterations, creativity is executed *T* times. Therefore, the total time complexity of the creativity phase is:
  (O(N×Dim)+O(N×M))×O(T)=O((Dim+M)NT)
- **Problem-solving**: This phase represents the exploitation phase of HTSO, with the execution process and frequency closely resembling those of the creativity phase. As a result, the time complexity is also:
  O((Dim+M)NT)
- **Decision-making**: During this stage, calculations for both fitness values and updated particle positions are not performed. The phase solely updates the population by comparing the fitness of the old and new particles. Each execution requires *N* fitness comparisons, performed twice per iteration, across *T* iterations. Thus, the time complexity of the decision-making phase is:
  O(2NT)

The four stages outlined above provide a brief overview of the components contributing to the time complexity of HTSO. By summing these stages, the total time complexity of HTSO is as follows:

O(N(Dim+M))+O((Dim+M)NT)+O((Dim+M)NT)+O(2NT)=O(N(Dim+M)(1+2T)+2T)

Therefore, the time complexity of HTSO, ignoring lower-order terms, is O((Dim+M)NT).

The pseudocode of the HTSO algorithm can be found in Algorithm 1.
**Algorithm 1** Pseudocode of HTSO.  1:**Input:** Population size *N*, Dimension *D*, lower bounds lb and upper bounds ub, Maximum number of Iterations *T* and Evaluation $FES_{max}$.  2:**Output:** The minimum fitness $F_{best}$ and the best solution $X_{best}$  3:**Initialization:** Initial population *X* and fitness *F* by Equation (5)–(7).  4:**while**  $t\;\leq \;T\;and\;fes\;\leq \;FES_{max}$  **do**  5:    Update population *X* by Equation (9)  6:    Calculate fitness *F* and update $F_{best}$ and $X_{best}$ by Equation (18)  7:    Update population *X* by Equation (15)  8:    Calculate fitness *F* and update $F_{best}$ and $X_{best}$ by Equation (18)  9:    t=t+1andfes=fes+2N10:**end while**11:**Return** The minimum fitness $F_{best}$ and the best solution $X_{best}$

## 4. The Results of the HTSO Algorithm on Different Test Sets

In this section, a comprehensive overview of the experimental findings for HTSO and its rival algorithms on the CEC benchmark suite is provided. Section 4.1 describes the experimental setup, Section 4.2 presents the results of the ablation study and parameter sensitivity analysis, and Section 4.3 analyzes the convergence behavior of HTSO. Detailed evaluations of the experimental outcomes on the CEC-2017, CEC-2020, and CEC-2022 benchmark sets are presented in Section 4.4, Section 4.5 and Section 4.6, respectively.

### 4.1. Experiment Settings

#### 4.1.1. Competing Algorithms and Parameter Settings

In the experiments conducted on the CEC benchmark set, we compare the performance of 22 algorithms, including the following:

- The HTSO algorithm newly proposed in this study.
- The winning algorithms of the CEC competition [123]:
  DE [53], LSHADE [124], LSHADE_SPACMA [125], LSHADE-cnEpSin [126].
- Algorithms or their improved versions that are widely recognized and applied:
  MELGWO [127], PPSO [128], WOA [30], SSA [32].
- Highly citepd and high-performance algorithms proposed in recent years:
  HO [49], GJO [38], DBO [43], SBOA [47], BKA [46], FTTA [103], SCSO [45], AO [37], SO [39], AVOA [35], GTO [36], RIME [74], SCA [78].

Table 2 presents the hyperparameter settings for the competitive algorithms. The performance of all algorithms is compared across three CEC test suites, covering a total of eight dimensions. The benchmark sets utilized in this study incorporate the CEC-2017 suite, which evaluates dimensions of 30, 50, and 100 [129]; the CEC-2020 suite, featuring tests in 10, 15, and 20 dimensions [130]; as well as the CEC-2022 suite, which includes dimensionalities of 10 and 20 [131]. The population size for all experiments was fixed at 30, and each experiment allowed up to 30,000 function evaluations. Every algorithm underwent 30 independent executions. The experimental results are summarized in Table S1 using four statistical indicators: mean, standard deviation (Std), best value, and worst value. Moreover, in the final row of each results table, the values cor1‘responding to the counts of wins (*W*), draws (*T*), and losses (*L*) are presented, summarizing how HTSO compares against other algorithms based on average performance. Here, ‘*W*’ stands for the total wins, ‘*T*’ signifies the number of ties, and ‘*L*’ indicates the losses obtained by HTSO.

To provide a more intuitive visualization of the experimental results, we employed heatmaps to illustrate the average optimization rankings of the 22 algorithms on each function, Sankey diagrams and stacked bar charts to depict the overall rankings of each algorithm, and line charts to display the Friedman mean ranks [132]. Subsequently, the Wilcoxon rank-sum test [133] with a significance level of p=0.05 was conducted to verify whether there are significant performance differences between HTSO and the competing algorithms, with the results reported in Table S2. In the table, ‘+’ indicates that HTSO performed significantly better than the competing algorithm on the corresponding function, ‘−’ indicates significantly worse performance, and ‘=’ denotes no significant difference. Consistent with the CEC experimental results, the outcomes of the Wilcoxon rank-sum test are also summarized in the table using the statistics ‘*W*’, ‘*T*’ and ‘*L*’. To visually illustrate the convergence rate, accuracy, and stability among the 22 algorithms, convergence curves and boxplots were employed in the final analysis.

All algorithms were executed under the same system configuration, which included a computer running the Windows 10 (64-bit) operating system, equipped with an Intel Xeon E-2224 3.40 GHz CPU and 16 GB of RAM. The experimental environment was established using MATLAB 2023a.

#### 4.1.2. Benchmark Functions

In this subsection, we thoroughly describe the categories of functions featured within the benchmark test suites of CEC-2017, CEC-2020, and CEC-2022.

Table 3 presents the function types included in the CEC-2017 test suite. In the CEC-2017 benchmark suite, F1 and F3 are classified as unimodal functions, which are utilized to evaluate how effectively the algorithm converges. Functions F4 to F10 are multimodal, crafted to evaluate the algorithm’s ability to avoid local optima during the search process. F11 through F20 are hybrid functions that have been rotated or translated, integrating three or more CEC-2017 benchmark functions and assigning specific weights to each sub-function. Functions F21 to F30 represent composite functions, each formed by integrating a minimum of three hybrid or CEC-2017 benchmark functions, which have undergone rotation and translation. Additionally, every individual sub-function is assigned specific bias values and weights. The intricate structure of these combined functions raises the level of challenge in algorithmic optimization.

A summary of the function categories included in the CEC-2020 test suite is provided in Table 4. This suite features a total of ten functions that pose significant difficulty: F1 serves as a unimodal function; F2 through F4 consist of rotated and translated multimodal functions; F5, F6, and F7 are classified as hybrid types; while F8 to F10 represent intricate composite functions.

Table 5 presents the function types of the CEC-2022 test suite. Within the CEC-2022 benchmark suite, F1 acts as a unimodal function. Functions F2 through F5 fall under the category of multimodal functions. The group comprising F6, F7, and F8 represents a combination of unimodal and multimodal characteristics. Lastly, F9 to F12 are described as composite functions that also exhibit multimodal properties.

### 4.2. Strategies Effectiveness and Parameter Sensitivity Analysis

This section presents an ablation study and a parameter sensitivity analysis. The former validates the effectiveness of the proposed strategies, while the latter examines the influence of parameters on algorithmic performance.

#### 4.2.1. Ablation Experiments

First, an ablation study is conducted to validate the effectiveness of the Creativity and Problem-solving components. Specifically, HTSO is compared against the following two variants:

- HTSO-α: HTSO without the Creativity component.
- HTSO-β: HTSO without the Problem-solving component.

The experimental settings are consistent with Section 4.1. Table S3 presents the experimental results along with the Wilcoxon rank-sum test results at a significance level of p=0.05. As shown in the last row of the CEC-2017 benchmark results, HTSO achieves a win/loss record of 25/4 against HTSO-α and 19/10 against HTSO-β. The Wilcoxon test results further reveal that HTSO significantly outperforms HTSO-α on 19 functions, shows no significant difference on 7 functions, and performs significantly worse on 3 functions. Similarly, compared to HTSO-β, HTSO significantly outperforms on 16 functions, exhibits no significant difference on 9 functions, and performs significantly worse on 4 functions.

The ablation study results demonstrate that HTSO significantly outperforms both HTSO-α and HTSO-β, confirming that the Creativity and Problem-solving components each play an essential role and are indispensable to the algorithm’s overall performance.

#### 4.2.2. Sensitivity Analysis

In HTSO, the Creativity component governs strategy selection through the dynamic parameter CT. An excessively large CT threshold leads to insufficient triggering of creative operations, thereby reducing population diversity and the ability to escape local optima. Conversely, an excessively small CT threshold may cause search behavior to become overly randomized, resulting in deviation from high-potential regions and weakened convergence stability. To identify an appropriate threshold that optimizes HTSO performance, a sensitivity analysis is conducted on the CT threshold. The threshold is varied across five values: 0.3, 0.4, 0.5, 0.6, and 0.7. Following the same experimental configuration as in Section 4.1, comparative experiments are performed on the 30-dimensional CEC-2017 benchmark suite to evaluate algorithm performance.

Table S4 presents the average results for CT threshold values of 0.3, 0.4, 0.5, 0.6, and 0.7. It can be observed that HTSO achieves the best average performance when the CT threshold is set to 0.5. Considering both solution optimality and robustness, CT=0.5 is selected as the default threshold setting in this study.

### 4.3. Convergence Behavior Analysis

**Figure 6:**
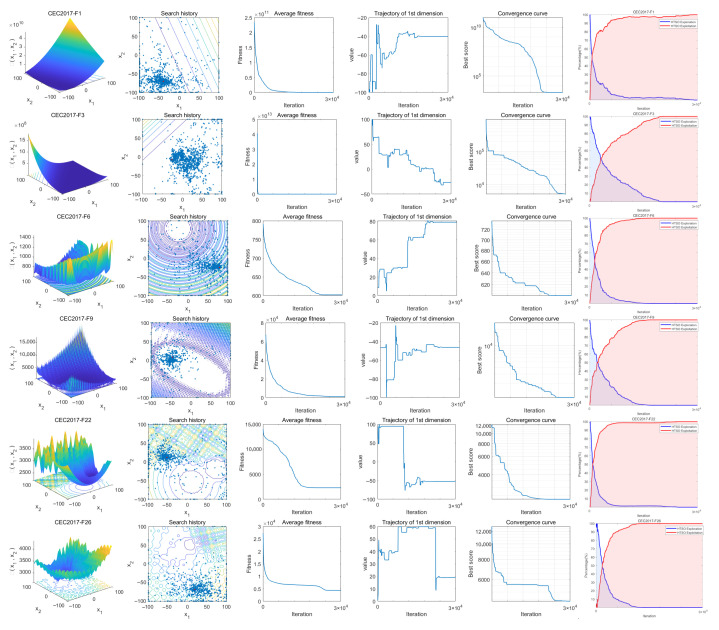
The convergence behavior of HTSO in the CEC-2017.

**Figure 7:**
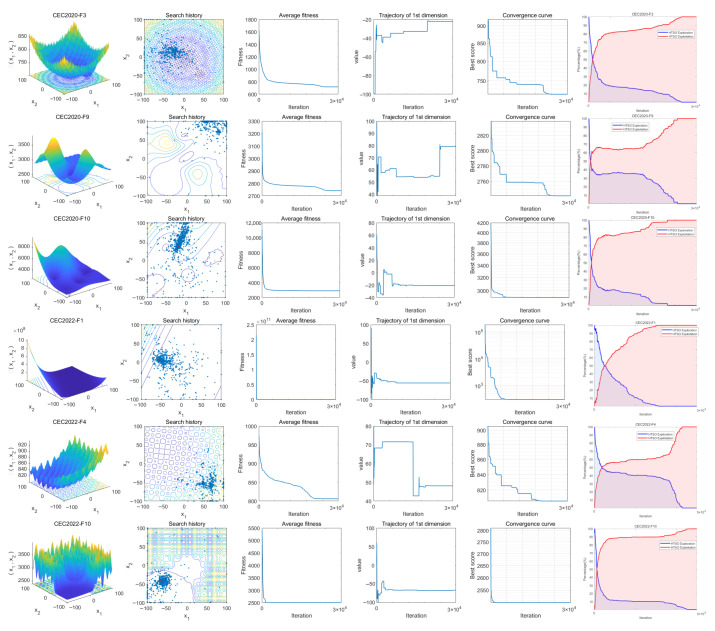
The convergence behavior of HTSO in the CEC-2020 and CEC-2022.

This section analyzes the exploration and convergence behavior of the HTSO algorithm through six subplots generated from the output runs on the CEC-2017, CEC-2020, and CEC-2022 test suites. Figure 6 and Figure 7 display the search behavior of HTSO on the CEC-2017 test suite and the CEC-2020 and CEC-2022 test suites, respectively. The following is a detailed analysis of the six subplots.

I. The initial column offers a three-dimensional visual depiction of the search space corresponding to the test functions.
II. The second column illustrates the search agents’ search history, highlighting that, throughout the process, they are broadly dispersed across the whole search space, showcasing impressive global search abilities. In the later stages of the search, they tightly converge around the optimal solution, indicating that HTSO possesses strong convergence abilities.
III. The variations in agents’ average fitness values throughout the search are shown in the third column. Initially, the agents are relatively scattered with varying fitness levels, suggesting a tendency towards global search. With the increase in iterations, the average fitness values decline swiftly, suggesting that the majority of search agents have the capability to locate the global optimal solution.
IV. The fourth column portrays the trajectories of individual agents during the search, transitioning from an initial fluctuating state to a stable condition. This process demonstrates a seamless shift from global to local search, which aids in acquiring the global optimal solution.
V. The fifth column displays the HTSO convergence curve, which gradually declines as iterations increase. This indicates HTSO’s ability to escape local optima and continue searching for the global optimal solution.
VI. The sixth column illustrates the balance between the exploration and exploitation phases of the HTSO algorithm. The figure illustrates that the exploration and exploitation curves of the HTSO algorithm converge around the 10% point; according to recent research, this suggests a remarkable equilibrium between its exploration and exploitation abilities.

### 4.4. Performance Comparison on the CEC-2017 Test Suite

#### 4.4.1. CEC-2017 Test Benchmark Functions Experimental Results

As shown in Table S1, the HTSO algorithm outperforms each comparison algorithm in all dimensions, with the value of ‘*W*’ consistently greater than ‘*L*’. According to the calculations, HTSO achieved first-place finishes 17, 18, and 18 times in the 30, 50, and 100 dimensions, respectively, out of comparisons across 29 test functions. In a total of 609 comparisons against 21 competing algorithms, HTSO won 572, 566, and 564 times in the 30, 50, and 100 dimensions, respectively, corresponding to winning rates of 93.92%, 92.94%, and 92.61%. This sustained high winning rate in 100-dimensional cases is particularly significant, as it demonstrates HTSO’s robustness against the curse of dimensionality, whereas many competing metaheuristics exhibit marked performance degradation in these high-dimensional search spaces.

#### 4.4.2. Ranking of the CEC-2017 Test Set

To enable intuitive comparison of the performance of the 22 algorithms on the CEC-2017 test set, this section presents the rankings of the algorithms according to mean fitness (Mean) using heatmaps, Sankey diagrams, and stacked bar charts for 30-, 50-, and 100-dimensional cases. Additionally, line graphs display the Friedman mean rankings of the 22 algorithms.

**Figure 8:**
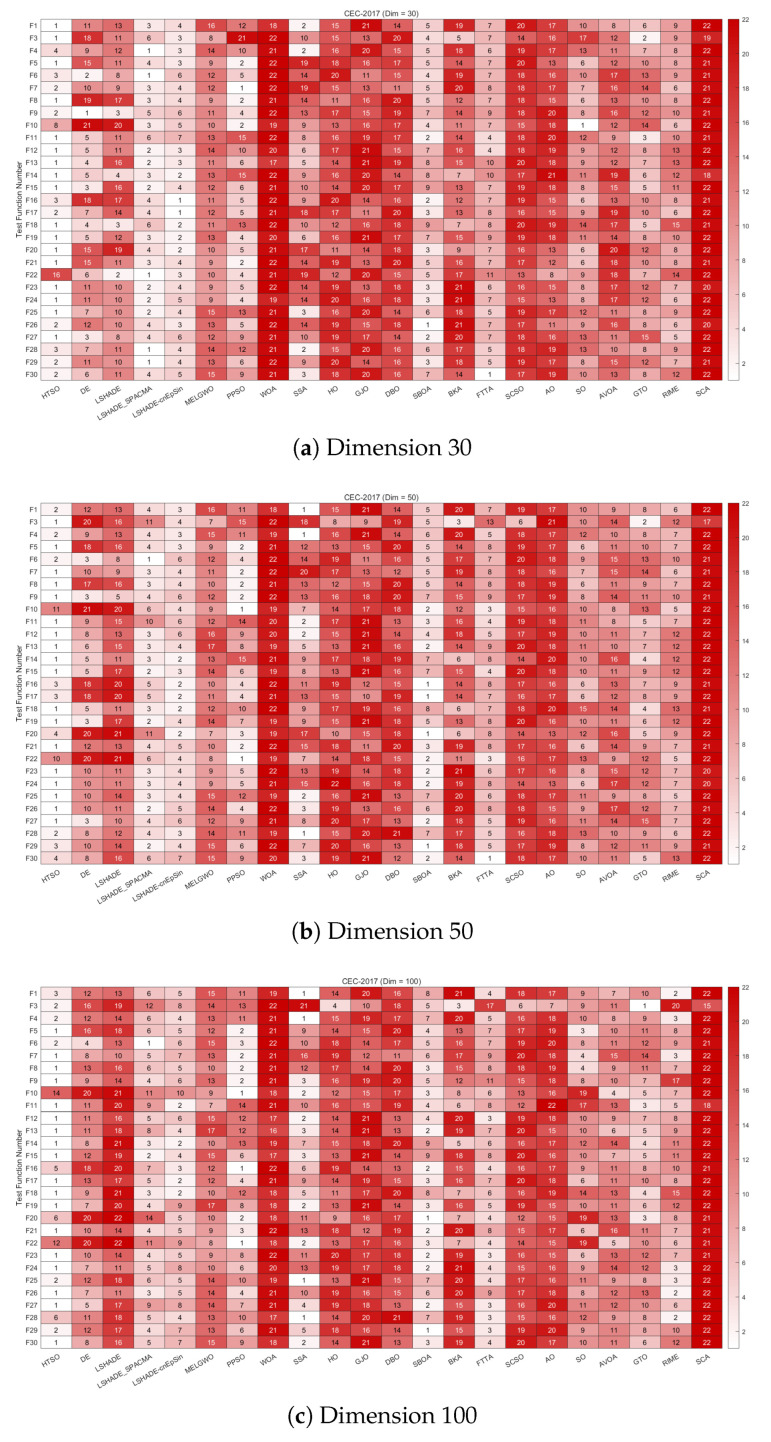
The Heat Map of the algorithm ranking of the CEC-2017.

Figure 8 shows the heatmaps of rankings across different functions and dimensions, providing a clear overview of each algorithm’s performance on every test function. Observations from the CEC-2017 test set, categorized by function type, are as follows:

- **Unimodal functions (F1 and F3)**: HTSO ranked first on F1 and F3 in 30 dimensions, and first on F3 in 50 dimensions, while also achieving top rankings on F1 in 50 and 100 dimensions, as well as F3 in 100 dimensions. Unimodal functions are important indicators of an algorithm’s convergence speed. Experimental results demonstrate that HTSO exhibits superior convergence speed on the CEC-2017 test set.
- **Multimodal functions (F4–F10)**: In 30 dimensions, HTSO achieved first place on F5 and F8, second on F7 and F9, and third on F6. In 50 and 100 dimensions, HTSO secured first place for F5, F7, F8, and F9 and second place for F4 and F6. It should be noted that despite its strong performance on most functions, HTSO performed poorly on F10, indicating that its search strategy is less effective for this function, leaving room for improvement. Multimodal functions contain numerous local optima, which makes it difficult for algorithms to avoid becoming trapped in these points. The results show that HTSO possesses a strong ability to escape local optima, enabling continued exploration of the solution space even in later iterations.
- **Hybrid functions (F11–F20)**: At 30 dimensions, HTSO ranked third on F16 and second on F17, while achieving first place on the remaining eight functions. At 50 dimensions, it again ranked third on F16 and F17, fourth on F20, and first on the remaining seven functions. In 100 dimensions, HTSO ranked fifth on F16 and sixth on F20, securing first place for the other eight functions. Hybrid functions are crucial for evaluating an algorithm’s performance, and HTSO demonstrates significant effectiveness and robustness across most functions of this type.
- **Composition functions (F21–F30)**: In 30 dimensions, HTSO was ranked first on F21, F23, F24, F25, and F27, and achieved top-three rankings on all functions except F22. In 50 dimensions, it ranked first on F21 and F23-F27, and placed second, third, and fourth on F28, F29, and F30, respectively. In 100 dimensions, HTSO ranked first on F21, F23, F24, F26, F27, and F30, and second on F25 and F29. Notably, HTSO performed poorly on F22 across all dimensions but performed exceptionally well on all other functions. As composition functions are highly complex, HTSO’s outstanding performance on these functions suggests its potential for solving complicated optimization problems.

Overall, HTSO demonstrates outstanding performance across most functions within the CEC-2017 test set. It not only converges rapidly and accurately but also shows a strong ability to avoid local optima and effectively addresses complex, high-dimensional optimization challenges.

**Figure 9:**
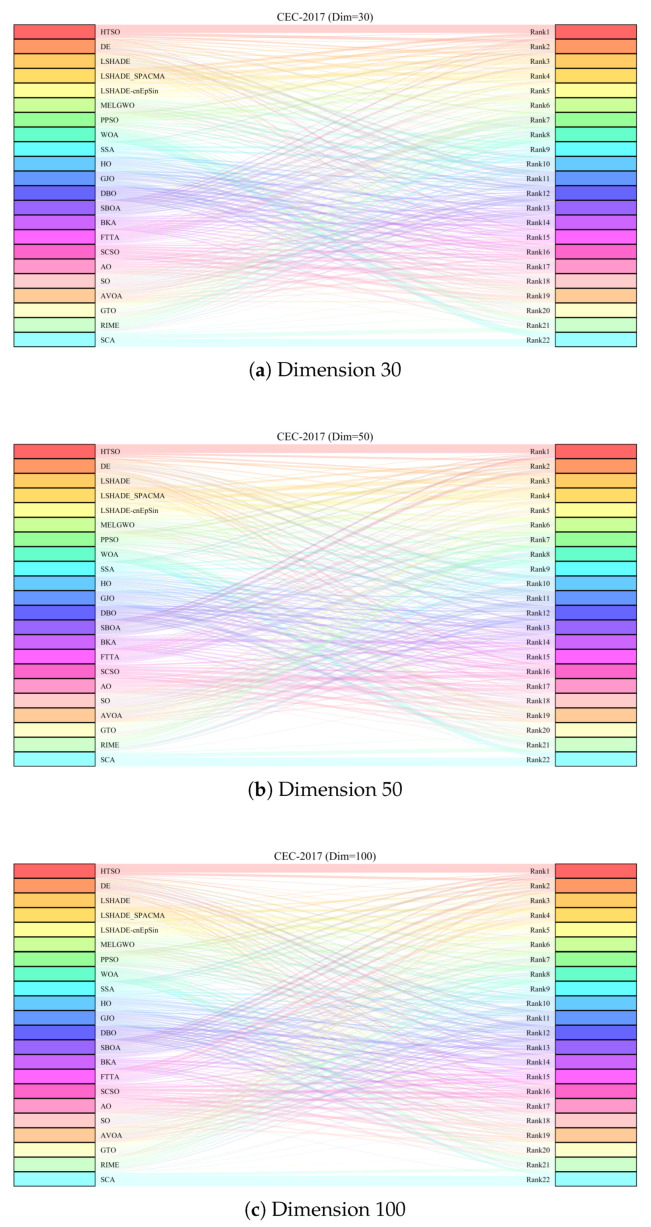
The Sankey diagrams of the algorithm ranking of the CEC-2017.

**Figure 10:**
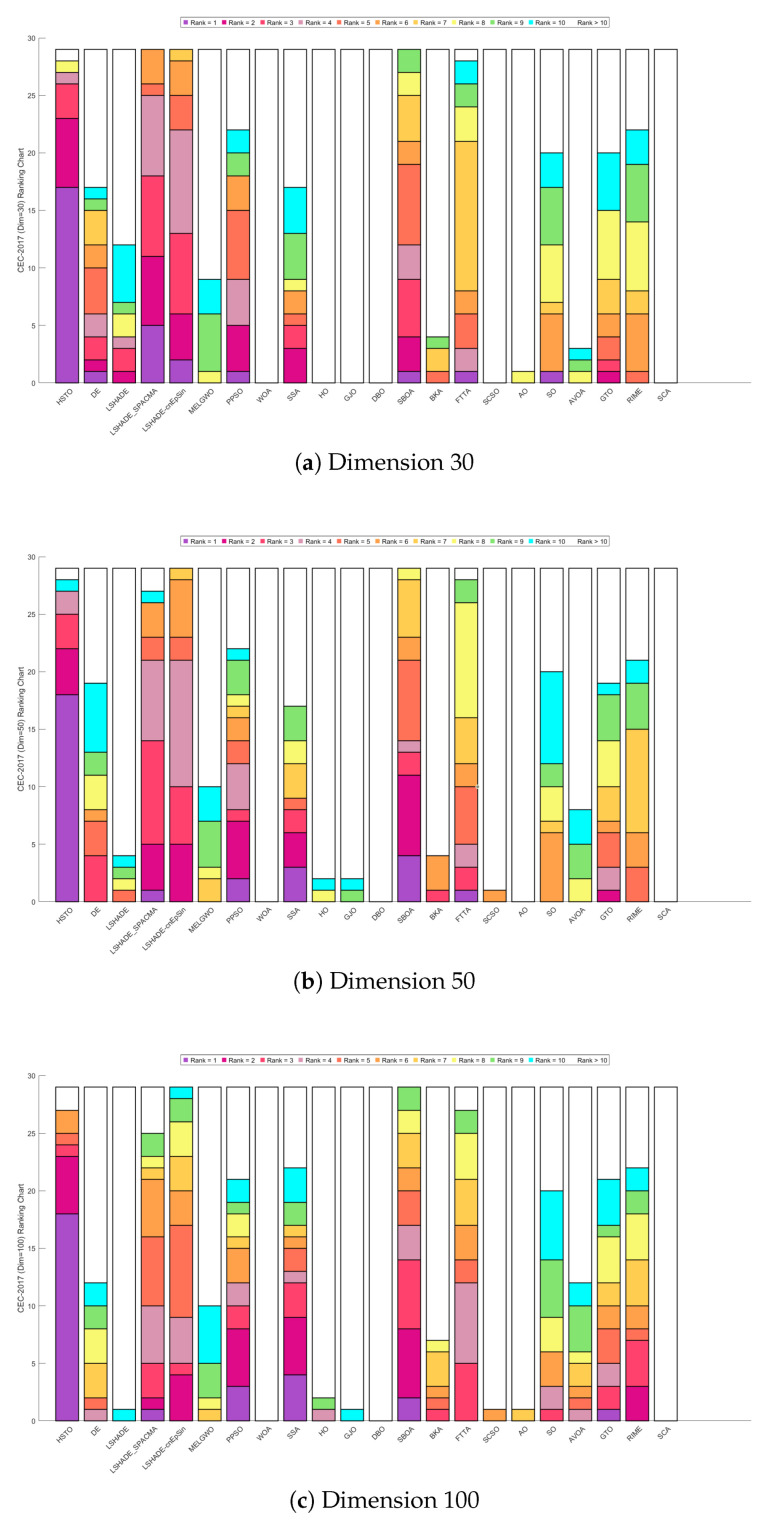
The Stacked bar charts of the algorithm ranking of the CEC-2017.

Figure 9 displays the Sankey diagrams for each dimension, where HTSO’s links to the ‘Rank 1’ node are the thickest among all 22 algorithms, indicating that HTSO achieved the most first-place rankings. Figure 10 presents the stacked bar charts for different dimensions, which clearly show that HTSO achieved the greatest number of first places across all dimensions. Specifically, in the 30- and 50-dimensional cases, only one function saw HTSO fail to enter the top ten, while in 100 dimensions, this occurred for two functions.

**Figure 11:**
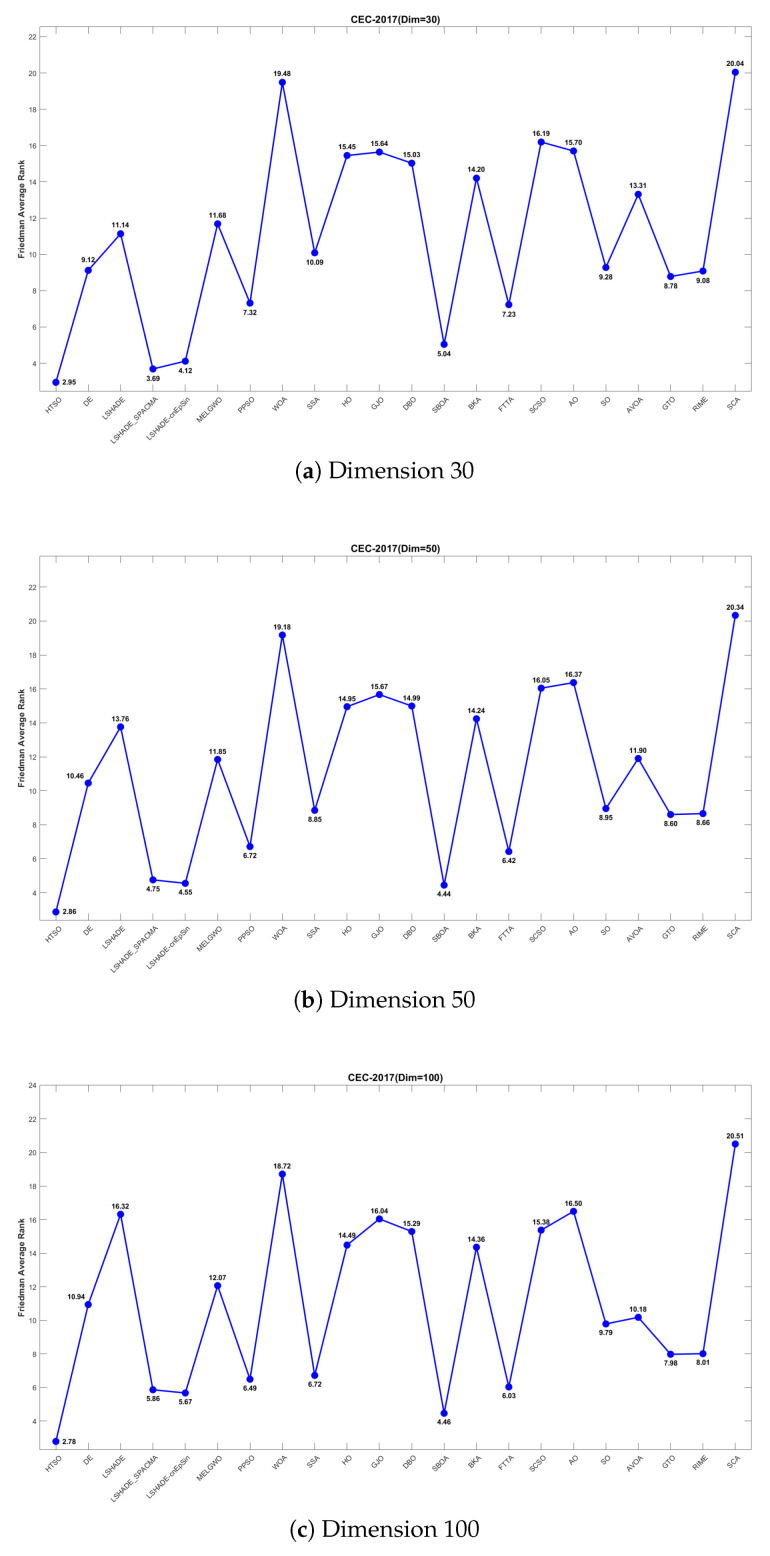
Friedman average ranking line charts of the CEC-2017.

Figure 11 further presents the line graphs of the Friedman mean rankings for each algorithm on the CEC-2017 test set across all dimensions. HTSO achieved the best average ranks: 2.95 for 30 dimensions (0.74 ahead of the second-place LSHADE_SPACMA at 3.69), 2.86 for 50 dimensions (1.58 ahead of second-place SBOA at 4.44), and 2.78 for 100 dimensions (1.68 ahead of second-place SBOA at 4.46). As the dimensionality increases, the gap between HTSO and the other algorithms widens, highlighting HTSO’s potential for high-dimensional optimization tasks.

#### 4.4.3. Wilcoxon Rank Sum Test of the CEC-2017 Test Set

Table S2 presents the results of the Wilcoxon rank-sum test, which lead to conclusions similar to those in Table S1. The test results show that the number of wins ‘*W*’ for HTSO in 30, 50, and 100 dimensions are 549, 549, and 550, respectively, corresponding to winning rates of 90.15%, 90.15%, and 90.31%. These results clearly indicate that HTSO is significantly different from other competing algorithms on the CEC-2017 test set.

#### 4.4.4. Convergence Curve of the CEC-2017 Test Set

**Figure 12:**
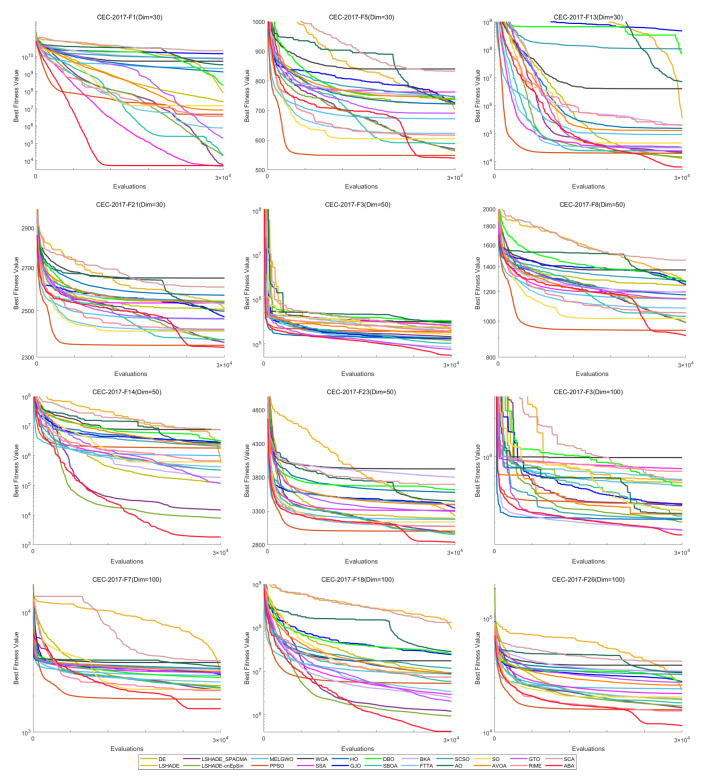
CEC-2017 test function convergence curve.

Figure 12 shows the convergence curves of 22 algorithms for selected functions on the CEC-2017 test set across various dimensions. The results demonstrate that, on unimodal functions F1 (Dim = 30), F3 (Dim = 50), and F3 (Dim = 100), HTSO rapidly converges to near-global optima in the early iterations, indicating strong exploitation capability. For multimodal functions F5 (Dim = 30), F8 (Dim = 50), and F7 (Dim = 100), while other algorithms are stuck in local optima late in the search, HTSO maintains particle diversity and continues to find better solutions, thereby sustaining competitive convergence accuracy. On hybrid functions F13 (Dim = 30), F14 (Dim = 50), F18 (Dim = 100), and composition functions F21 (Dim = 30), F23 (Dim = 50), F26 (Dim = 100)—especially for F14 (Dim = 50), F18 (Dim = 100), F23 (Dim = 50), and F26 (Dim = 100)—HTSO maintains fast convergence and retains the ability to discover new local optima in later iterations. This demonstrates that the introduction of the Lévy flight strategy during the exploitation phase effectively helps particles escape from local optima and explore previously unvisited areas, thereby improving convergence accuracy.

#### 4.4.5. Boxplot of the CEC-2017 Test Set

**Figure 13:**
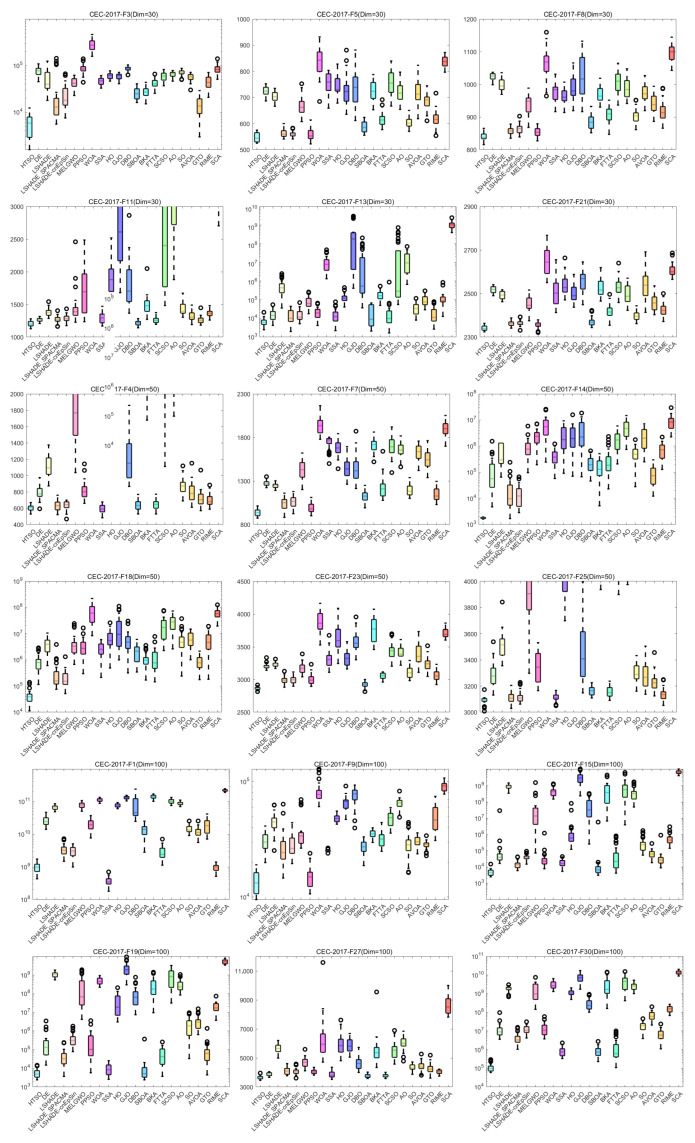
CEC-2017 test function boxplots.

Figure 13 presents the boxplots for the 22 algorithms based on 30 independent runs on functions F3, F5, F8, F11, F13, F21 (30 dimensions); F4, F7, F14, F18, F23, F25 (50 dimensions); and F1, F9, F15, F19, F27, F30 (100 dimensions) of the CEC-2017 test set. Note that some of the y-axes in the boxplots use exponential scales; thus, algorithms with more stable performance and higher convergence accuracy may display wider boxes. The results indicate that, for F5, F8, F11, and F13 (30 dimensions); F4, F7, F14, F18, F23, and F25 (50 dimensions); and F15, F19, F27, and F30 (100 dimensions), HTSO consistently exhibits the narrowest box. Additionally, for F3, F5, F8, F11, and F21 (30 dimensions); F4, F7, and F14 (50 dimensions); and F1 and F9 (100 dimensions), HTSO shows no outliers. Notably, on F14 (Dim = 50), where most algorithms have outliers and fail to converge stably near the global optimum, HTSO achieves a narrow box without outliers, reflecting both high convergence accuracy and stability.

These functions span all evaluated dimensions and encompass unimodal, multimodal, hybrid, and composition functions. Experimental results consistently demonstrate that HTSO not only achieves high convergence accuracy but also exhibits excellent stability and robustness.

### 4.5. Performance Comparison on the CEC-2020 Test Suite

#### 4.5.1. CEC-2020 Test Benchmark Functions Experimental Results

The experimental results of the CEC-2020 test suite in the 10-dimensional, 15-dimensional, and 20-dimensional cases are provided in Table S1. HTSO achieved first-place average results 4 times, 4 times, and 5 times in the 10-dimensional, 15-dimensional, and 20-dimensional cases, respectively. As shown in the final row (W|T|L), HTSO consistently satisfies W≤L compared to other algorithms. In 210 comparisons with 21 algorithms across 10 test functions, HTSO recorded *W* counts of 188, 191, and 186 for the 10-dimensional, 15-dimensional, and 20-dimensional cases, respectively, representing 89.52%, 90.95%, and 88.57% of the total comparisons.

#### 4.5.2. Ranking of the CEC-2020 Test Set

**Figure 14:**
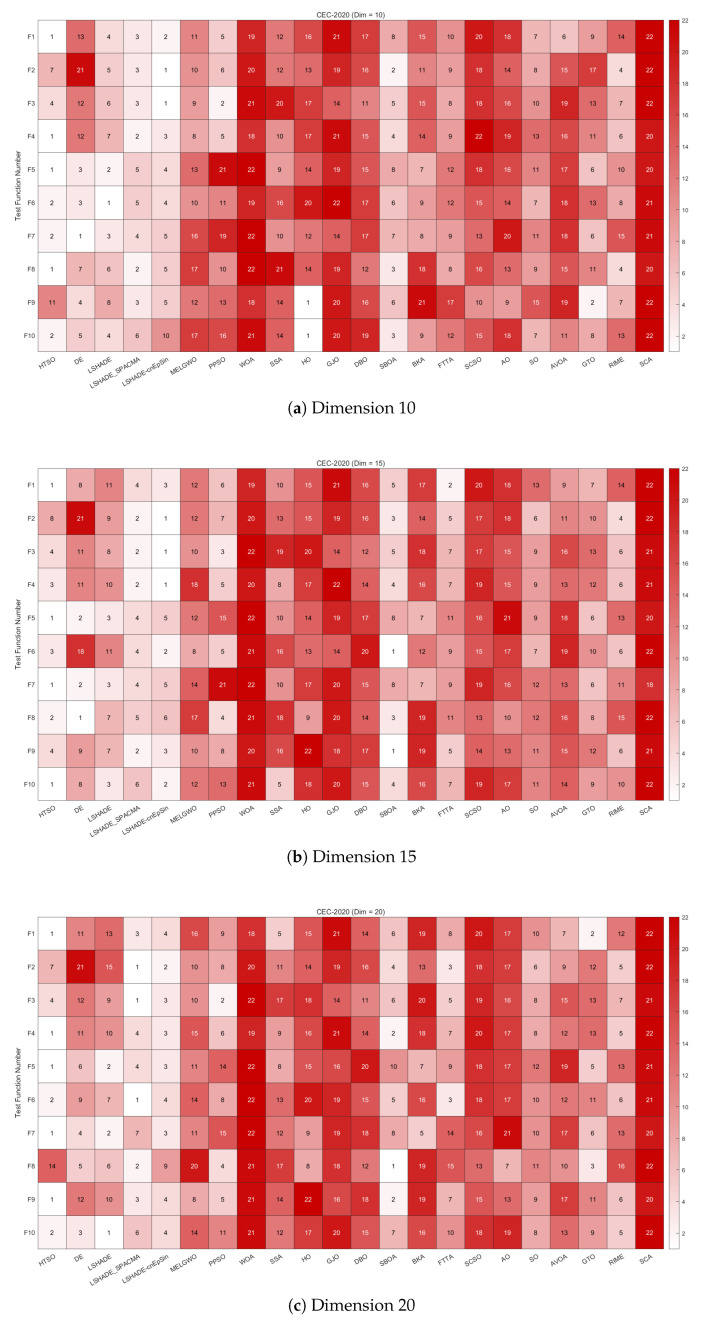
The Heat Map of the algorithm ranking of the CEC-2020.

Figure 14 provides heatmaps depicting the rankings of 22 algorithms on each function of the CEC-2020 test set for 10, 15, and 20 dimensions. HTSO achieved first place on F1, F4, F5, and F8 in the 10-dimensional case; on F1, F5, F7, and F10 in the 15-dimensional case; and on F1, F4, F5, F7, and F9 in the 20-dimensional case. This demonstrates that HTSO has the potential to achieve excellent performance across various types of functions and problem dimensions.

**Figure 15:**
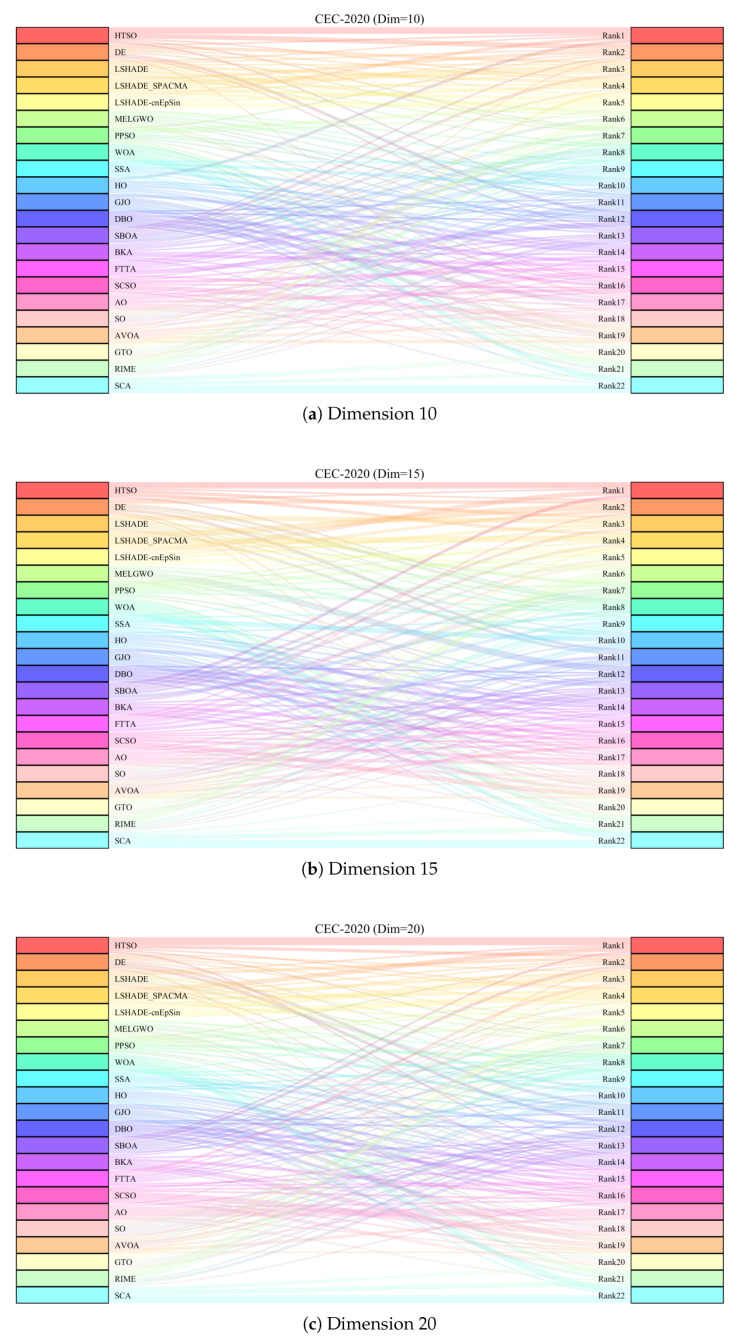
The Sankey diagrams of the algorithm ranking of the CEC-2020.

**Figure 16:**
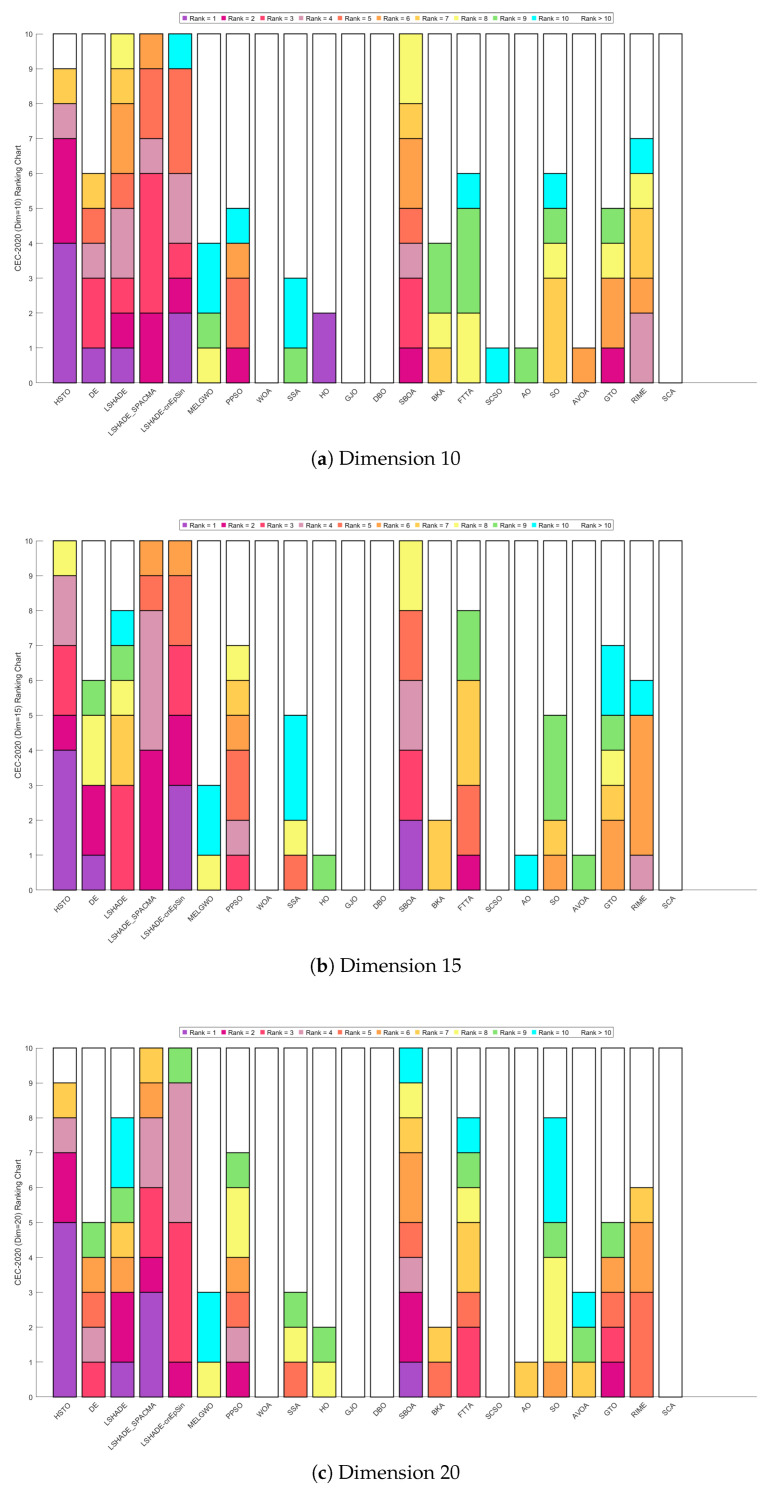
The Stacked bar charts of the algorithm ranking of the CEC-2020.

Figure 15 presents the Sankey diagrams of algorithm rankings, and Figure 16 shows the stacked bar charts of rankings on the CEC-2020 test set. It is evident that HTSO obtained the largest number of first-place positions in 10, 15, and 20 dimensions.

**Figure 17:**
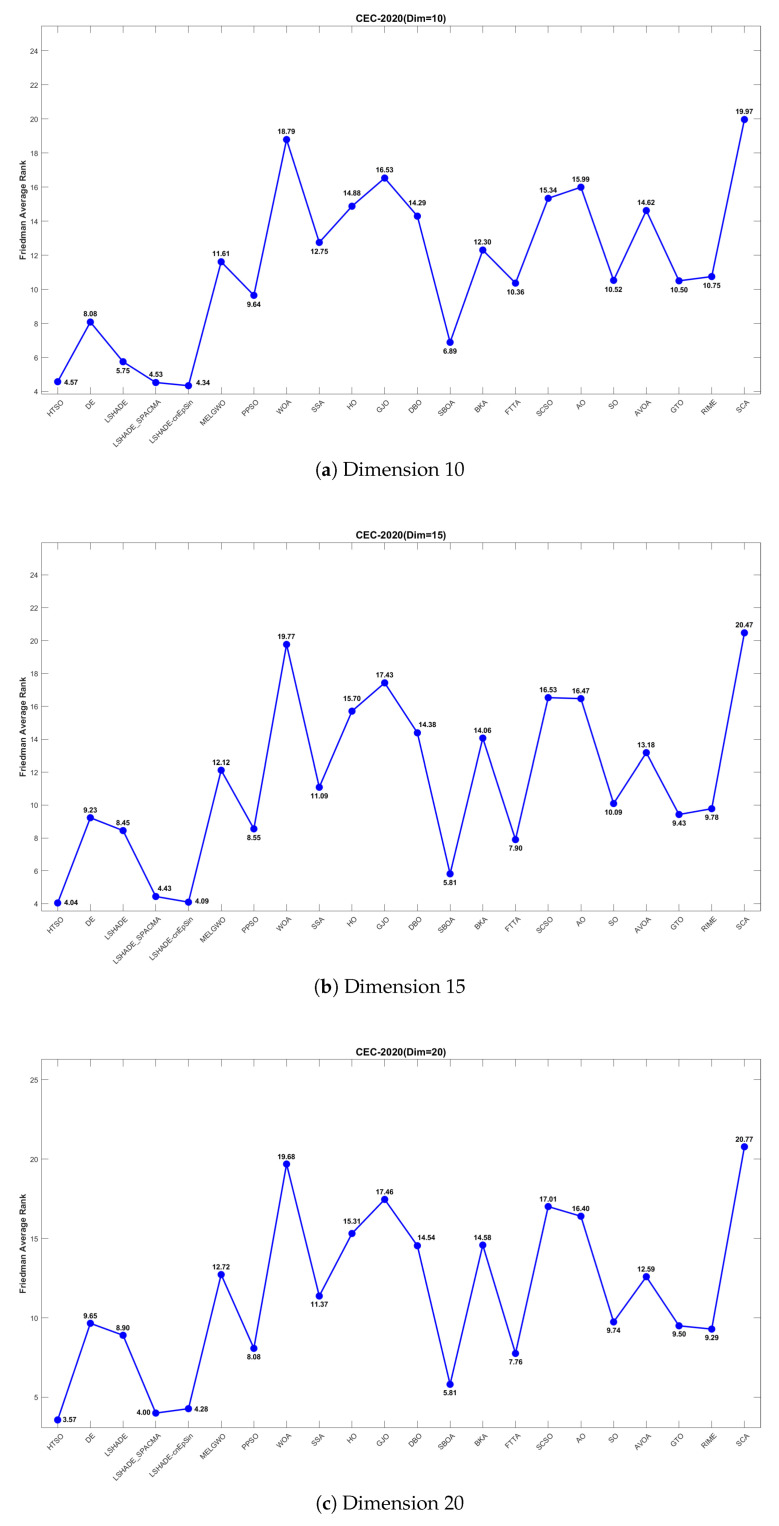
Friedman average ranking line charts of the CEC-2020.

Figure 17 further illustrates the Friedman mean ranking curves for the 22 algorithms. For 10-dimensional problems, LSHADE-cnEpSin ranked first (mean rank = 4.34), followed by LSHADE_SPACMA (4.53), and HTSO (4.57). In 15 dimensions, HTSO was ranked first (4.04), followed by LSHADE-cnEpSin (4.09) and LSHADE_SPACMA (4.43). For 20 dimensions, HTSO achieved first place (3.57), followed by LSHADE_SPACMA (4.00), and LSHADE-cnEpSin (4.28). It can be observed that in the 10-dimensional case, HTSO did not achieve the best results and performed slightly worse than LSHADE-cnEpSin and LSHADE_SPACMA. This is because LSHADE-cnEpSin and LSHADE_SPACMA, which have been repeatedly enhanced and have received awards in previous CEC competitions, exhibit exceptionally strong performance. However, these algorithms also present significant drawbacks, including substantial computational expense and complicated implementation processes. In contrast, HTSO has not undergone further improvements, yet achieves high convergence accuracy through its intrinsic search strategy and balanced exploitation-exploration mechanism. As the dimensionality increases, HTSO surpasses LSHADE-cnEpSin and LSHADE_SPACMA and further widens the performance gap, indicating substantial potential in addressing optimization challenges characterized by high dimensionality and complexity.

#### 4.5.3. Wilcoxon Rank Sum Test of the CEC-2020 Test Set

Table S2 presents the results of 210 Wilcoxon tests across three dimensions, with ‘*W*’ counts of 171, 178, and 181, corresponding to 81.43%, 84.76%, and 86.19% of the total tests. These results indicate that as the dimensionality increases, the frequency of ‘*W*’ occurrences in the Wilcoxon tests gradually increases, suggesting that HTSO shows considerable potential in addressing high-dimensional problems in the CEC-2020 test suite.

#### 4.5.4. Convergence Curve of the CEC-2020 Test Set

**Figure 18:**
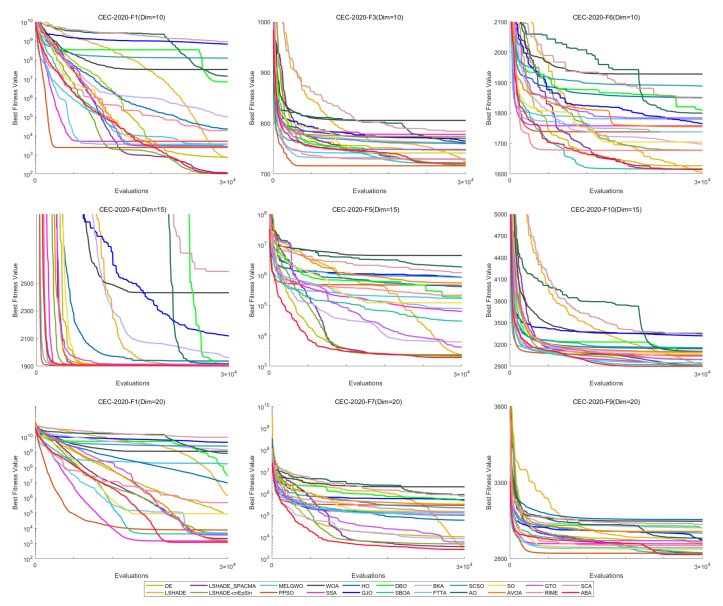
CEC-2020 test function convergence curve.

Figure 18 presents the convergence curves of selected functions with varying dimensions from the CEC-2020 test suite. Among the nine functions illustrated, HTSO achieves the highest convergence accuracy and maintains a relatively fast convergence speed on F1 in 10 dimensions; on F5, F10, and F14 in 15 dimensions; and on F1, F7, and F9 in 20 dimensions. Notably, for hybrid functions such as F5 in 15 dimensions and F7 in 20 dimensions, HTSO rapidly converges to the vicinity of the global optimum from the early stages of the iteration process.

#### 4.5.5. Boxplot of the CEC-2020 Test Set

**Figure 19:**
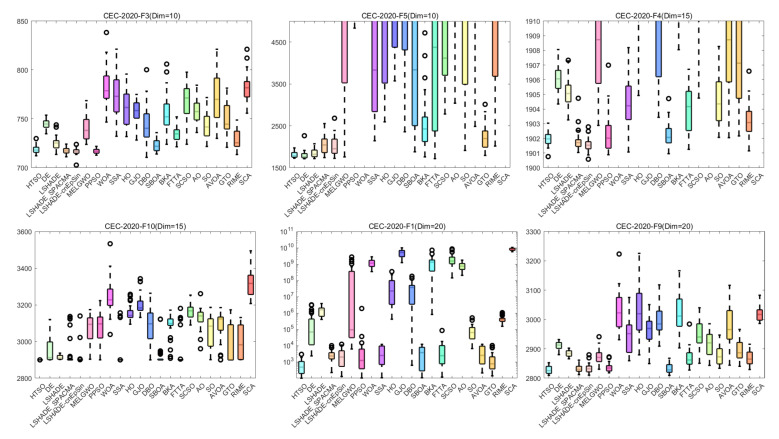
CEC-2020 test function boxplots.

Figure 19 presents boxplots of various types of functions with different dimensions from the CEC-2020 test suite. HTSO performs on par with the CEC competition-winning algorithms across the six functions, but displays a significantly narrower box than traditional optimization algorithms. In particular, for the 15-dimensional composition function F10, the other 21 algorithms generally suffer from low convergence accuracy, overly wide boxes, and outlying values with poor performance. In contrast, HTSO achieves high convergence accuracy, extremely narrow boxplots, and outliers with relatively good performance, indicating its strong potential for certain application scenarios.

### 4.6. Performance Comparison on the CEC-2022 Test Suite

#### 4.6.1. CEC-2022 Test Benchmark Functions Experimental Results

In this section, we evaluate how HTSO performs on the CEC-2022 test set. Detailed results under 10- and 20-dimensional settings can be found in Table S1. As shown in the last row, HTSO outperformed 21 algorithms in 252 comparisons, winning 219 times in 10 dimensions and 238 times in 20 dimensions, which corresponds to a success rate of 86.90% and 94.44%, respectively. HTSO achieved the highest average ranking 4 times in 10 dimensions and 6 times in 20 dimensions.

#### 4.6.2. Ranking of the CEC-2022 Test Set

**Figure 20:**
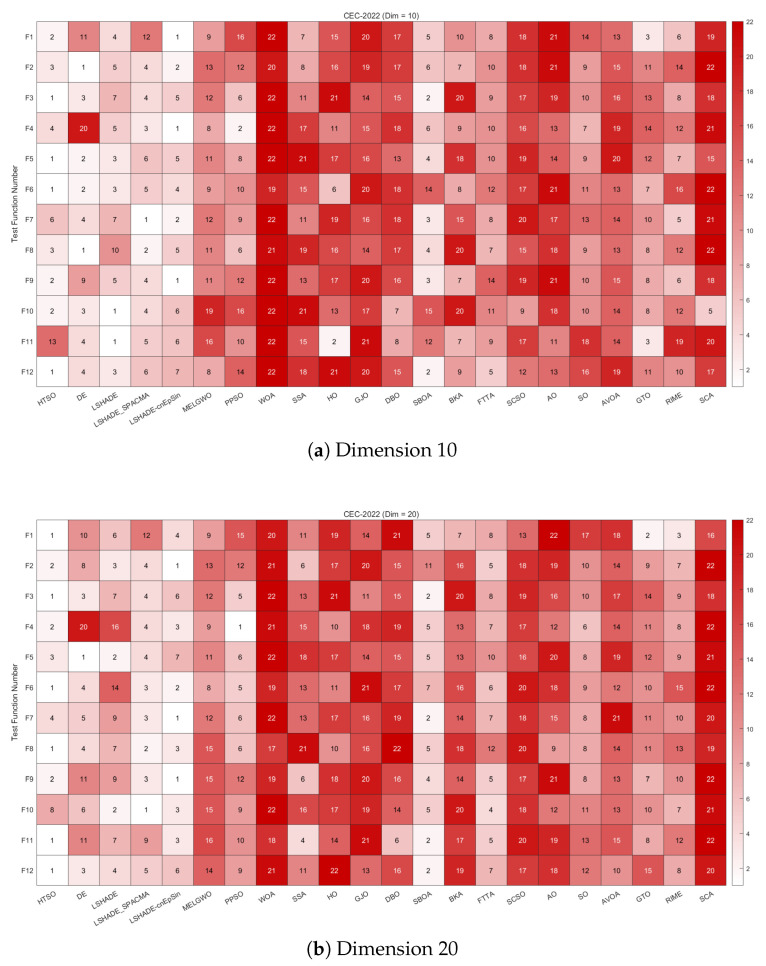
The Heat Map of the algorithm ranking of the CEC-2022.

Figure 20 shows a heatmap of the rankings of 22 algorithms on the CEC-2022 test set for both 10-dimensional and 20-dimensional problems. The results indicate that HTSO achieves outstanding performance across various types of functions. Specifically, HTSO ranks first on F3, F5, F6, and F12 in the 10-dimensional case, as well as on F1, F3, F6, F8, F11, and F12 in the 20-dimensional case. Furthermore, HTSO consistently ranks within the top three on all functions except F4, F7, and F11 for 10 dimensions, and F7 and F8 for 20 dimensions.

**Figure 21:**
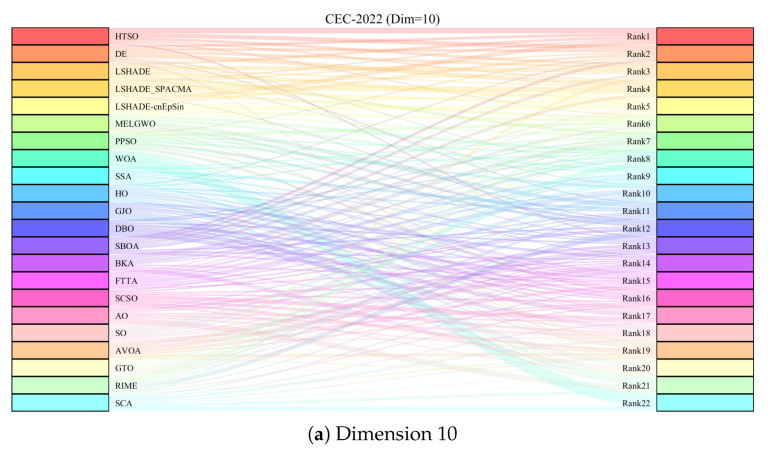

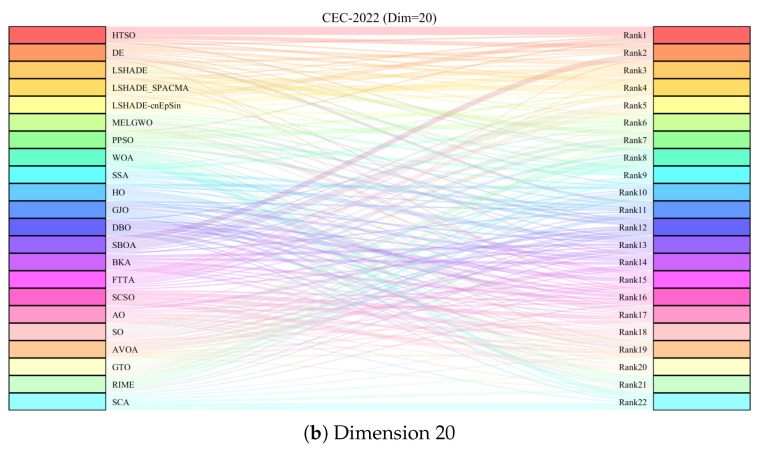
The Sankey diagrams of the algorithm ranking of the CEC-2022.

**Figure 22:**
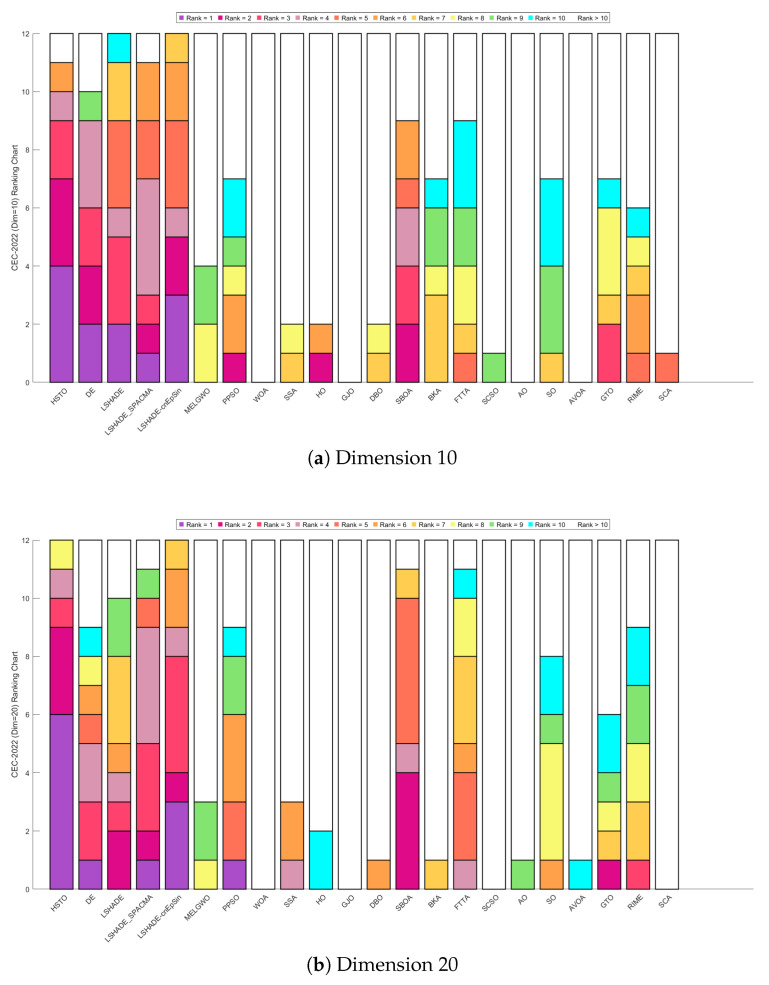
The Stacked bar charts of the algorithm ranking of the CEC-2022.

Figure 21 and Figure 22 present the Sankey diagrams and stacked bar charts for the rankings of the 22 algorithms in 10 and 20 dimensions, respectively. As observed, HTSO obtains the most first-place rankings in both scenarios. Notably, in the 20-dimensional case, HTSO ranks within the top ten for all twelve functions.

**Figure 23:**
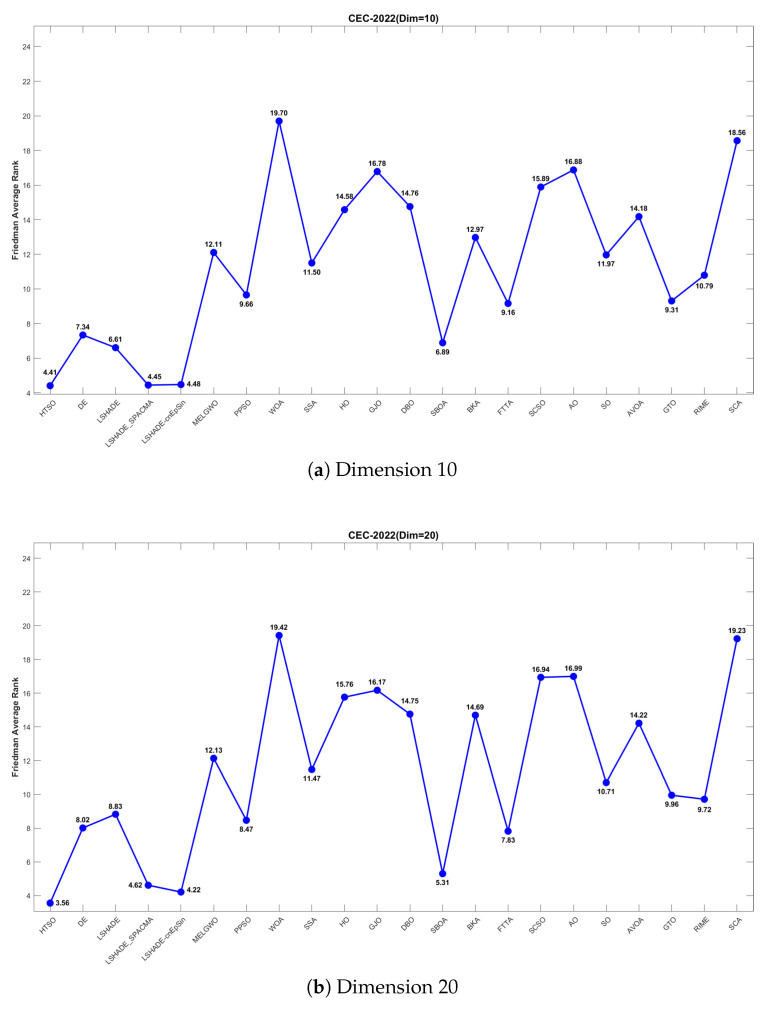
Friedman average ranking line charts of the CEC-2022.

The Friedman mean rank plots for both the 10-dimensional and 20-dimensional scenarios are illustrated in Figure 23. For the 10-dimensional problems, HTSO ranks first with an average rank of 4.41, followed by LSHADE_SPACMA (4.45) and LSHADE-cnEpSin (4.48). For the 20-dimensional problems, HTSO maintains the leading position with an average rank of 3.56, while LSHADE-cnEpSin and LSHADE_SPACMA achieve average ranks of 4.22 and 4.62, respectively. These results show that the advantage of HTSO over the CEC competition-winning algorithms (LSHADE_SPACMA and LSHADE-cnEpSin) is not significant at 10 dimensions, but this advantage becomes more pronounced at 20 dimensions, demonstrating the strong competitiveness of HTSO in high-dimensional optimization problems.

#### 4.6.3. Wilcoxon Rank Sum Test of the CEC-2022 Test Set

Table S2 summarizes the outcomes obtained from the Wilcoxon test. In the Wilcoxon tests for 10 and 20 dimensions, HTSO showed statistically significant results 193 and 220 times, respectively, corresponding to success rates of 76.59% and 87.30%. Overall, HTSO demonstrated excellent performance on the CEC-2022 test set. However, when compared with CEC award-winning algorithms in low-dimensional functions, HTSO’s performance was less favorable. According to the statistical results in Appendix A, HTSO’s comparison with DE in 10 dimensions showed ‘W=L’, while the comparison with LSHADE-cnEpSin indicated that ‘*W*’ was slightly smaller than ‘*L*’. The Wilcoxon test results also showed that in 10 dimensions, HTSO underperformed in comparisons with LSHADE_SPACMA and LSHADE-cnEpSin. In contrast, in 20 dimensions, HTSO significantly surpassed four CEC award-winning algorithms, demonstrating its ability to adapt to the complexity and uncertainty of high-dimensional optimization problems.

#### 4.6.4. Convergence Curve of the CEC-2022 Test Set

**Figure 24:**
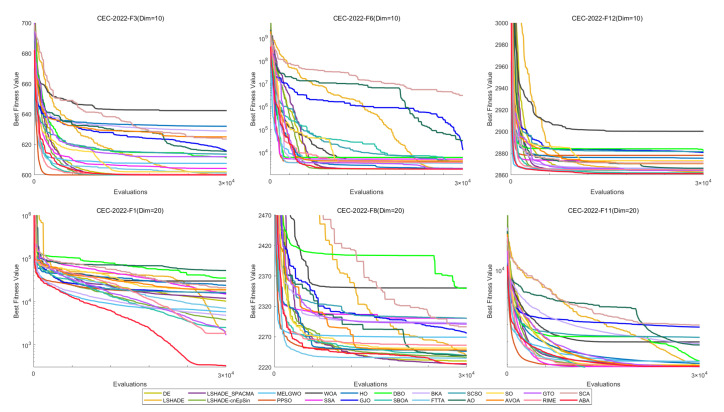
CEC-2022 test function convergence curve.

Figure 24 presents the convergence curves for selected functions in both 10-dimensional and 20-dimensional cases. These functions were chosen to represent different categories, and across all the functions shown, HTSO not only demonstrates superior convergence precision but also exhibits advantageous convergence speed. In particular, for the 20-dimensional F1 function, HTSO demonstrates a consistently faster convergence rate than all other algorithms throughout the entire iteration process. Additionally, on the 10-dimensional F12 function, HTSO outperforms the majority of algorithms in terms of convergence speed.

#### 4.6.5. Boxplot of the CEC-2022 Test Set

**Figure 25:**
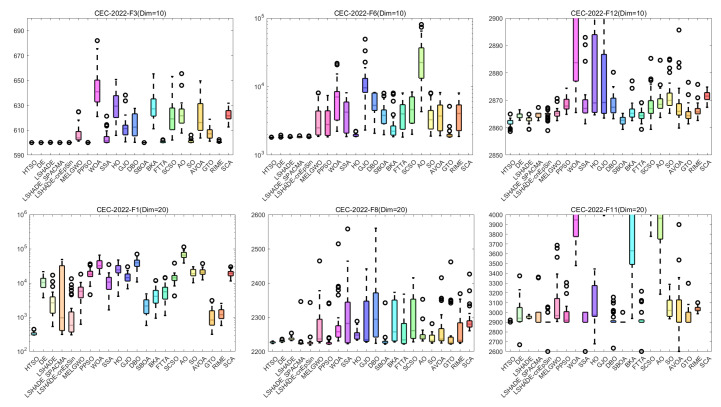
CEC-2022 test function boxplots.

Boxplots illustrating representative functions under both 10-dimensional and 20-dimensional scenarios are displayed in Figure 25. For all functions shown, HTSO exhibits narrower boxes and fewer outliers, indicating higher stability. Specifically, for the 10-dimensional F3 and F6 functions, both HTSO and the CEC competition-winning algorithms demonstrate greater stability compared to other traditional optimization algorithms. However, for the other functions displayed, HTSO clearly demonstrates superior stability. Additionally, although HTSO does not achieve the best solution for the 20-dimensional F11 function when compared to DE, LSHADE-cnEpSin, SSA, HO, DBO, FTTA, and AVOA, it has a significantly narrower box. This indicates that HTSO offers better stability than these algorithms.

### 4.7. Summary of the CEC Test Set Experiment

In this section, we first present three-dimensional illustrations of selected functions from the CEC-2017, CEC-2020, and CEC-2022 test sets, the population dynamics during the global optimal search process of HTSO, and the balance between the exploration and exploitation phases throughout the iterative process. The results demonstrate that HTSO not only converges effectively in the later stages of iteration but also maintains an optimal balance between exploration and exploitation, ensuring excellent performance.

Subsequently, we evaluate 22 algorithms, including HTSO, using various metrics: mean, standard deviation, best value, worst value, ranking heatmaps, Sankey diagrams, stacked bar charts, Friedman average ranking line charts, Wilcoxon test, convergence curves and boxplots. The evaluation is conducted on the CEC-2017 test set at 30, 50, and 100 dimensions; the CEC-2020 test set at 10, 15, and 20 dimensions; and the CEC-2022 test set at 10 and 20 dimensions. The results demonstrate that HTSO exhibits rapid convergence, exceptional stability, and significant potential for solving high-dimensional optimization problems, outperforming the other 21 algorithms. Most notably, HTSO’s superior performance on the 100-dimensional problems distinguishes it from many peer algorithms that tend to degrade significantly in high dimensions.

However, despite the overall superior performance, it is necessary to explicitly acknowledge the limitations of the proposed algorithm. As observed in the convergence analysis, the convergence speed of HTSO is sometimes moderate, specifically when compared to highly specialized competition winners in lower-dimensional scenarios. Furthermore, a convergence lag was observed on specific function types, notably the multimodal function F10 and composition function F22 in the CEC-2017 suite. In these specific cases, HTSO showed a tendency to converge slower or struggle to escape local optima compared to the top-performing competitors. This suggests that while the algorithm excels in high-dimensional and complex landscapes, further improvements could be made to enhance its rapid convergence capability in low-dimensional or highly deceptive environments.

## 5. Real-World Constrained Optimization Problems

This section compares the HTSO algorithm with 14 competing algorithms on 12 classic real-world constrained optimization problems(COPs) to evaluate its applicability and effectiveness. The 14 competing algorithms are GWO [28], BKA [46], AVOA [35], HO [49], GTO [36], RIME [74], SCA [78], FTTA [103], WOA [30], SO [39], GJO [38], DBO [43], SCSO [45], and GBO [80]. These include both widely used algorithms and recent high-performance algorithms, with their hyperparameters listed in Table 2. The population size was set to 30, and the maximum number of fitness evaluations to 30,000. Each algorithm is independently run 10 times, and the mean, standard deviation, best value, and worst value are computed as statistical metrics, followed by a Wilcoxon rank-sum test.

The 12 COPs are as follows:

- Three-bar Truss Design Problem (TBTD) [135];
- Tension/Compression Spring Design (Case 1) (TCPD (case 1)) [136];
- Pressure Vessel Design (PVD) [137];
- Gear Train Design (GTD) [138];
- Hydro-Static Thrust Bearing design (HSTB) [139];
- Multiple Disk Clutch Brake Design Problem(MDCB) [140];
- Planetary Gear Train Design(PGTD) [141];
- Gas Transmission Compressor Design (GTCD) [140];
- Rolling Element Bearing Design (REBD) [142];
- Tension/Compression String Design Problem (Case 2) (TCPD (case 2)) [143];
- Weight Minimization of a Speed Reducer (WMSR) [144];
- 10-bar Truss Design Problem (10-BTD) [145].

### 5.1. Three-Bar Truss Design Problem (TBTD)

Minimizing the overall weight of a truss while ensuring compliance with mechanical constraints is the central objective of the well-known Three-bar Truss Design Problem in structural optimization. The optimization objective is to enhance material efficiency, ensuring that the structure can support the design load with minimal mass. This problem is primarily related to the optimization of strength and stiffness, ensuring that the structure is both safe and efficient under load. In practice, similar optimization techniques are widely applied in fields such as bridge design, construction, and aerospace, effectively reducing material costs, minimizing environmental impact, and improving structural reliability and durability. Equation (19) provides the formulation of the mathematical model corresponding to this problem.

(19) $$\begin{array}{cc} \mathrm{Minimize}: & f\left(\vec{x}\right)=l\left(x_{2}+2\sqrt{2}x_{1}\right)\\ \mathrm{subject}\;\mathrm{to}: & g_{1}\left(\vec{x}\right)=\frac{x_{2}}{2x_{2}x_{1}+\sqrt{2}x_{1}^{2}}P-\sigma \leq 0,\\ \; & g_{2}\left(\vec{x}\right)=\frac{x_{2}+\sqrt{2}x_{1}}{2x_{2}x_{1}+\sqrt{2}x_{1}^{2}}P-\sigma \leq 0,\\ \; & g_{3}\left(\vec{x}\right)=\frac{1}{x_{1}+\sqrt{2}x_{2}}P-\sigma \leq 0.\\ \mathrm{where}: & l=100,\;P=2,\;\mathrm{and}\;\sigma =2.\\ \mathrm{with}\;\mathrm{bounds}: & 0\leq x_{1},x_{2}\leq 1. \end{array}$$

**Figure 26:**
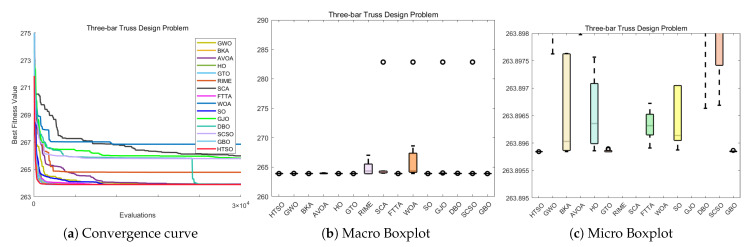
The convergence curve and boxplot of TBTD.

This subsection presents the experimental results of 15 algorithms on the TBTD problem (Table 6). HTSO achieved both the optimal and mean value of 263.8958. It ranked first in both best and mean metrics, and the Wilcoxon rank-sum test indicates its performance is significantly superior to that of the other 14 algorithms. Figure 26a shows the convergence curves of all algorithms; HTSO clearly converges first, demonstrating its superior convergence capability on the TBTD problem.

Figure 26b provides a macro-level view of the stability of 15 algorithms on the TBTD problem. It shows that SCA, WOA, GJO, and SCSO exhibit outliers, indicating poor stability. Among them, WOA not only shows outliers but also has a larger interquartile range, suggesting that its stability on the TBTD problem is the weakest among the 15 algorithms. Additionally, while RIME does not present outliers, its larger interquartile range indicates relatively poor performance on the TBTD problem. Figure 26c offers a micro-level view of the same data. Although the results of the remaining 10 algorithms are quite similar in Figure 26b, Figure 26c reveals subtle differences among them. Clearly, HTSO and GBO exhibit the best performance on the TBTD problem, with GTO also performing very similarly to HTSO and GBO.

### 5.2. Tension/Compression Spring Design (Case 1) (TCPD (Case 1))

TCPD (case 1) represents a well-known problem in structural optimization, where the primary goal is to reduce the weight of the spring while adhering to specified mechanical performance requirements. The parameters to be determined are the wire diameter, the mean coil diameter, and the number of active coils. Optimization enhances the spring’s strength, stiffness, and service life, ensuring stability and reliability under various loads while enabling a more compact, lightweight, and efficient structure. Springs are widely employed in automotive, machinery, and electronic applications; optimized design can substantially reduce material costs, improve performance, and decrease energy consumption, delivering significant engineering and economic benefits. The formulation of the mathematical model corresponding to TCPD (Case 1) can be found in Equation (20).

(20) $$\begin{array}{cc} \mathrm{Minimize}: & f\left(\vec{x}\right)=x_{1}^{2}x_{2}\left(2+x_{3}\right)\\ \mathrm{subject}\;\mathrm{to}: & g_{1}\left(\vec{x}\right)=1-\frac{x_{2}^{2}x_{3}}{71785x_{1}^{4}}\leq 0,\\ \; & g_{2}\left(\vec{x}\right)=\frac{4x_{2}^{2}-x_{1}x_{2}}{12566(x_{2}x_{1}^{3}-x_{1}^{4})}+\frac{1}{5108x_{1}^{2}}-1\leq 0,\\ \; & g_{3}\left(\vec{x}\right)=1-\frac{140.45x_{1}}{x_{2}^{2}x_{3}}\leq 0,\\ \; & g_{4}\left(\vec{x}\right)=\frac{x_{1}+x_{2}}{1.5}-1\leq 0.\\ \mathrm{with}\;\mathrm{bounds}: & 0.05\leq x_{1}\leq 2.00,\\ \; & 0.25\leq x_{2}\leq 1.30,\\ \; & 2.00\leq x_{3}\leq 15.0. \end{array}$$

Table 7 presents the results of 15 algorithms on the TCPD (Case 1) problem. HTSO attained the highest mean rank and best rank, achieving a global optimum of $1.267\times 10^{-2}$ and an average result of $1.267\times 10^{-2}$. According to the results of the Wilcoxon test, HTSO demonstrates a statistically significant advantage over the other fourteen algorithms when applied to the TCPD (Case 1) problem.

**Figure 27:**
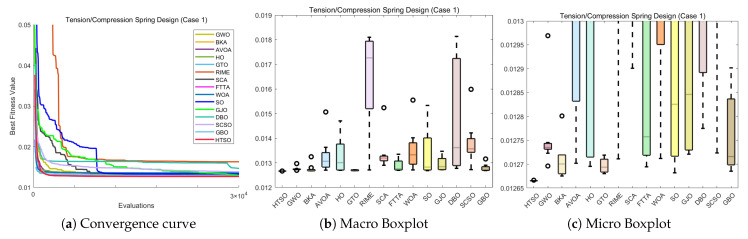
The convergence curve and boxplot of TCPD (case 1).

Figure 27a illustrates the convergence curve for the TCPD (Case 1) problem. As shown in the figure, HTSO is not the fastest converging algorithm for this problem but outperforms most other algorithms. Although HTSO converges slightly slower than some algorithms, the rankings in Table 7 reveal that those algorithms with faster convergence ultimately produce weaker results than HTSO. This suggests that, overall, HTSO demonstrates superior performance.

Figure 27b,c show the macro and micro boxplots for the TCPD (Case 1) problem, respectively. It is evident that most algorithms exhibit unstable performance on this problem, with only HTSO, GWO, BKA, and GTO demonstrating relatively optimistic results. Among these, GTO is the only algorithm without any outliers, while HTSO, GWO, and BKA exhibit one or two outliers, indicating that GTO’s performance is particularly stable. However, as shown in Figure 27c, although GTO’s performance is stable, the Best value it achieved is still inferior to HTSO’s Worst value. Despite the presence of outliers, HTSO consistently outperforms the other algorithms.

### 5.3. Pressure Vessel Design (PVD)

The objective of Pressure Vessel Design (PVD) is to reduce manufacturing expenses for a vessel while adhering to limitations related to structural integrity and strength. Common design variables encompass the thicknesses of the shell and head, as well as the length and radius of the vessel. The problem must satisfy pressure constraints to ensure the vessel does not yield, fracture, or lose stability under high internal pressure. By optimizing the design, material usage is reduced and manufacturing and transportation costs are lowered without compromising safety. Pressure vessels are widely used in the chemical, energy, and aerospace industries; their structural optimization is essential for enhancing equipment safety, extending service life, and conserving resources, thereby driving the development of more efficient and reliable engineering designs. Equation (21) provides the mathematical formulation used for Pressure Vessel Design (PVD).

(21) $$\begin{array}{cc} \mathrm{Minimize}: & f\left(\vec{x}\right)=1.7781z_{2}x_{3}^{2}+0.6224z_{1}x_{3}x_{4}+3.1661z_{1}^{2}x_{4}+19.84z_{1}^{2}x_{3}\\ \mathrm{subject}\;\mathrm{to}: & g_{1}\left(\vec{x}\right)=0.00954x_{3}\leq z_{2},\\ \; & g_{2}\left(\vec{x}\right)=0.0193x_{3}\leq z_{1},\\ \; & g_{3}\left(\vec{x}\right)=x_{4}\leq 240,\\ \; & g_{4}\left(\vec{x}\right)=-\pi x_{3}^{2}x_{4}-\frac{4}{3}\pi x_{3}^{3}\leq -1,296,000.\\ \mathrm{where}: & z_{1}=0.0625x_{1},\\ \; & z_{2}=0.0625x_{2}.\\ \mathrm{with}\;\mathrm{bounds}: & 0.05\leq x_{1}\leq 2.00,\\ \; & 0.25\leq x_{2}\leq 1.30,\\ \; & 2.00\leq x_{3}\leq 15.0. \end{array}$$

Table 8 presents the experimental results for the PVD problem, with HTSO achieving the first place in Mean rank and second place in Best rank. Meanwhile, as indicated by the standard deviation (Std), the standard deviations of the other algorithms are in the hundreds or even thousands, whereas HTSO’s Std is only $7.9\times 10^{-10}$. This demonstrates that HTSO’s performance is exceptionally stable for this problem.

**Figure 28:**
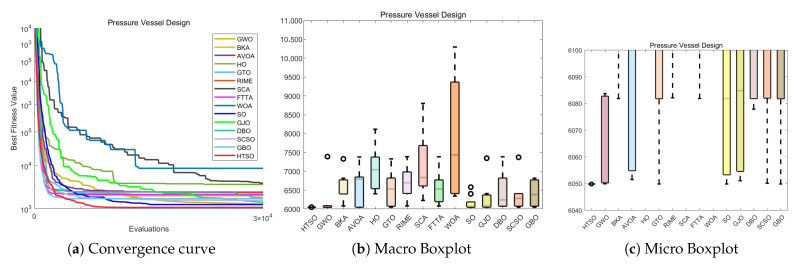
The convergence curve and boxplot of PVD.

The convergence curve associated with the Pressure Vessel Design (PVD) problem is depicted in Figure 28a. As shown in the figure, although HTSO’s convergence speed is slower than that of some algorithms in the early iterations, the fitness values of other algorithms stop decreasing after a certain point, indicating that they have become trapped in local optima and are unable to escape. In contrast, HTSO’s fitness continues to decrease, suggesting that HTSO retains a strong ability to escape local optima even after convergence.

Figure 28b,c present the macro and micro boxplots for the PVD problem, respectively. From Figure 28b, it is evident that all 13 algorithms, except for GWO, perform significantly worse than HTSO. In Figure 28b, the performance gap between HTSO and GWO appears relatively small, except for a single poor outlier value from GWO. However, Figure 28c clearly demonstrates that HTSO consistently achieves a fitness value equivalent to the optimal value obtained by GWO in every run, providing strong evidence of a substantial performance gap between HTSO and GWO.

### 5.4. Gear Train Design (GTD)

The optimization of gear train design aims to minimize the cost of the gear system while ensuring transmission efficiency and power transfer stability. Through optimization, transmission efficiency can be improved, energy loss reduced, and a more compact and durable gear system achieved. In real-world applications, gear transmissions are widely used in industries such as automotive, machinery, and aerospace. Optimized designs contribute to enhanced performance, reduced energy consumption, extended service life, and lower maintenance costs. Therefore, optimizing gear systems is of significant economic and technical importance for improving the overall efficiency and reliability of industrial equipment. The mathematical formulation of the GTD problem is presented in Equation (22).

(22) $$\begin{array}{cc} \mathrm{Minimize}: & f\left(\vec{x}\right)={\left(\frac{1}{6.931}-\frac{x_{1}x_{2}}{x_{3}x_{4}}\right)}^{2}\\ \mathrm{with}\;\mathrm{bounds}: & 12\leq x_{1},x_{2},x_{3},x_{4}\leq 60. \end{array}$$

The experimental findings related to the GTD problem are shown in Table 9. In this comparison, HTSO obtains a Best value of 2.7009 × 10^−12^, as well as a Mean value of 1.0852 × 10^−11^. Notably, HTSO secures the top position in both Best and Mean rankings. Furthermore, according to the Wilcoxon test, there exists a statistically significant difference in performance between HTSO and the remaining fourteen algorithms.

**Figure 29:**
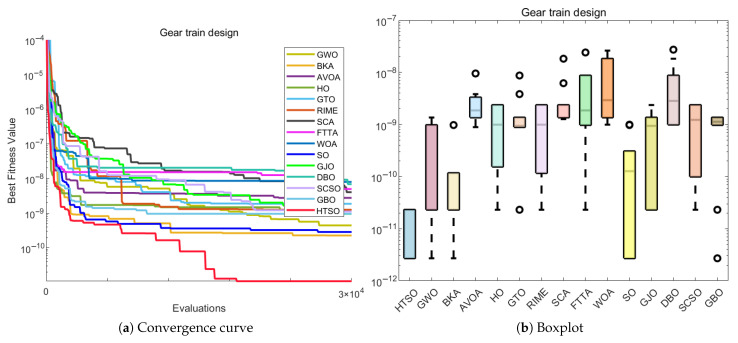
The convergence curve and boxplot of GTD.

The convergence curve for the GTD problem is depicted in Figure 29a. It is clearly observed that after converging to approximately $10^{-9}$, only HTSO’s fitness continues to decrease, further demonstrating its exceptional ability to escape local optima.

Figure 29b presents a boxplot for the GTD problem. It is evident that most algorithms exhibit outliers or whiskers, while HTSO shows neither outliers nor visible whiskers, with all results falling within the box. This indicates that all data points are within the interquartile range, demonstrating HTSO’s consistent performance on the GTD problem. Although the box for HTSO appears larger in the plot, the vertical axis is presented in logarithmic scale, indicating that the actual box size is relatively small.

### 5.5. Hydro-Static Thrust Bearing Design Problem (HSTB)

The optimization of the hydro-static thrust bearing (HSTB) design aims to minimize system energy consumption while ensuring the bearing’s load capacity and operational stability. The HSTB design process incorporates four key variables-oil viscosity, bearing radius, flow velocity, and recess radius—while also considering seven nonlinear constraints associated with factors such as inlet oil pressure, load-carrying capacity, oil film thickness, and related inlet pressure parameters. The objective of the optimization is to ensure that the bearing can effectively bear the load, reduce friction losses, and extend service life. In practical applications, static liquid pressure thrust bearings are widely used in high-speed rotating machinery, aerospace, precision instruments, and other fields, particularly in applications requiring low friction and high load-bearing capacity. Optimization of the design can significantly improve equipment efficiency and reliability, reduce energy consumption, and decrease maintenance frequency. This is of critical importance for enhancing the performance of high-end equipment, energy conservation, emission reduction, and ensuring long-term reliable operation, especially in modern manufacturing and high-precision industries. Equation (23) presents the mathematical formulation for the HSTB problem.

(23) $$\begin{array}{cc} \mathrm{Minimize}: & f\left(\vec{x}\right)=\frac{QP_{0}}{0.7}+E_{f}\\ \mathrm{subject}\;\mathrm{to}: & g_{1}\left(\vec{x}\right)=1000-P_{0}\leq 0,\\ \; & g_{2}\left(\vec{x}\right)=W-101,000\leq 0,\\ \; & g_{3}\left(\vec{x}\right)=5000-\frac{W}{\pi (R^{2}-R_{0}^{2})}\leq 0,\\ \; & g_{4}\left(\vec{x}\right)=50-P_{0}\leq 0,\\ \; & g_{5}\left(\vec{x}\right)=0.001-\frac{0.0307}{386.4P_{0}}\left(\frac{Q}{2\pi Rh}\right)\leq 0,\\ \; & g_{6}\left(\vec{x}\right)=R-R_{0}\leq 0,\\ \; & g_{7}\left(\vec{x}\right)=h-0.001\leq 0.\\ \mathrm{where}: & W=\frac{\pi P_{0}}{2}\frac{R^{2}-R_{0}^{2}}{ln\left(\frac{R}{R_{0}}\right)},P_{0}=\frac{6\mu Q}{\pi h^{3}}ln\left(\frac{R}{R_{0}}\right),\\ \; & E_{f}=9336Q\times 0.0307\times 0.5△T,△T=2\left(10^{p}-559.7\right),\\ \; & P=\frac{log_{10}log_{10}\left(8.122\times 10^{6}\mu +0.8\right)+3.55}{10.04},\\ \; & h={\left(\frac{2\pi \times 750}{60}\right)}^{2}\frac{2\pi \mu }{E_{f}}\left(\frac{R^{4}}{4}-\frac{R_{0}^{4}}{4}\right).\\ \mathrm{with}\;\mathrm{bounds}: & 1\leq R\leq 16,\\ \; & 1\leq R_{0}\leq 16,\\ \; & 1\times 10^{-6}\leq \mu \leq 16\times 10^{-6},\\ \; & 1\leq Q\leq 16. \end{array}$$

**Figure 30:**
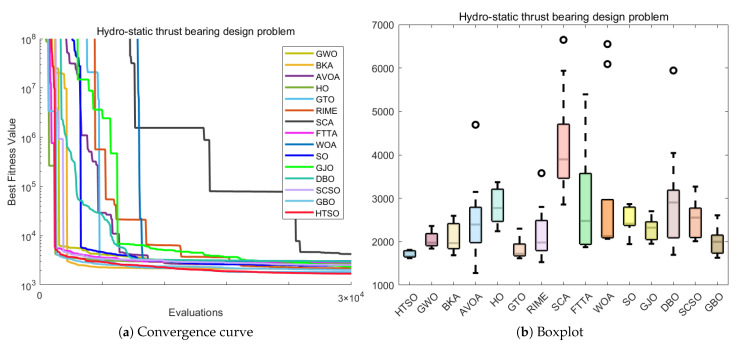
The convergence curve and boxplot of HSTB.

Table 10 presents the experimental results. HTSO achieved an average result of 1698.4286 and an optimal result of 1621.4566, with a mean rank of first place. However, the best rank for HTSO was fourth, falling short of the optimal solution. The top three rankings were occupied by AVOA, RIME, and GTO, respectively. According to the boxplot in Figure 30b, AVOA and RIME exhibited larger boxes and outliers approaching 5000 and 4000, respectively. Furthermore, AVOA and RIME had standard deviations of 916 and 608, while HTSO had a standard deviation of 74. The boxplot also reveals that HTSO has the narrowest box, indicating that, although HTSO did not achieve the optimal solution in the HSTB problem, it still demonstrated superior stability. Additionally, the Wilcoxon test shows that HTSO significantly outperforms the other 13 algorithms, though the performance gap with GTO is not significant.

The Figure 30a illustrates the convergence curve of the HSTB problem. It demonstrates that, even in the later stages of iteration, when the fitness of other algorithms shows little improvement, HTSO continues to search for new global optima. This further confirms that HTSO possesses a stronger ability to escape local optima.

### 5.6. Multiple Disk Clutch Brake Design Problem (MDCB)

The multiple disk clutch brake (MDCB) is widely used in automotive, industrial machinery, and aerospace applications, particularly in scenarios requiring high load, high torque transmission, and precise control. The objective of optimizing the MDCB design is to reduce the mass of the multi-disk clutch brake. The parameters considered encompass inner and outer radii, disk thickness, actuator force, and the total number of friction surfaces, alongside factors such as frictional behavior, pressure distribution, structural stiffness, and resistance to wear. The optimization design must ensure that the clutch and brake system maintain stable performance during prolonged operation, preventing overheating, rapid wear, or excessive heat generation. Equation (24) provides the mathematical representation of this problem.

(24) $$\begin{array}{cc} \mathrm{Minimize}: & f\left(\vec{x}\right)=\pi \left(x_{2}^{2}-x_{1}^{2}\right)x_{3}\left(x_{5}+1\right)\rho \\ \mathrm{subject}\;\mathrm{to}: & g_{1}\left(\vec{x}\right)=-p_{max}+p_{rz}\leq 0,\\ \; & g_{2}\left(\vec{x}\right)=p_{rz}V_{sr}-V_{sr,max}p_{max}\leq 0,\\ \; & g_{3}\left(\vec{x}\right)=▵R+x_{1}-x_{2}\leq 0,\\ \; & g_{4}\left(\vec{x}\right)=-L_{max}+\left(x_{5}+1\right)\left(x_{3}+\delta \right)\leq 0,\\ \; & g_{5}\left(\vec{x}\right)=sM_{s}-M_{h}\leq 0,\\ \; & g_{6}\left(\vec{x}\right)=-T\leq 0,\\ \; & g_{7}\left(\vec{x}\right)=-V_{s\tau ,max}+V_{s\tau }\leq 0,\\ \; & g_{8}\left(\vec{x}\right)=T-T_{max}\leq 0.\\ \mathrm{where}: & M_{h}=\frac{2}{3}\mu x_{4}x_{5}\frac{x_{2}^{3}-x_{1}^{3}}{x_{2}^{2}-x_{1}^{2}}N.\mathrm{mm},\\ \; & \omega =\frac{\pi n}{30}\mathrm{rad}/s,\\ \; & A=\pi \left(x_{2}^{2}-x_{1}^{2}\right){\mathrm{mm}}^{2},\\ \; & p_{rz}=\frac{x_{4}}{A}N/{\mathrm{mm}}^{2},\\ \; & V_{sr}=\frac{\pi R_{sr}n}{30}\mathrm{mm}/s,\\ \; & R_{sr}=\frac{2}{3}\frac{x_{2}^{3}-x_{1}^{3}}{x_{2}^{2}x_{1}^{2}}\mathrm{mm},\\ \; & T=\frac{I_{2}\omega }{M_{h}+M_{f}},\\ \; & R_{sr}=\frac{2}{3}\frac{x_{2}^{3}-x_{1}^{3}}{x_{2}^{2}x_{1}^{2}}\mathrm{mm},\\ \; & T=\frac{I_{2}\omega }{M_{h}+M_{f}},\\ \; & ▵R=20\mathrm{mm},L_{max}=30\mathrm{mm},\mu =0.6,\\ \; & V_{sr,max}=10m/s,\delta =0.5\mathrm{mm},s=1.5,\\ \; & T_{max}=15s,n=250\mathrm{rpm},I_{z}=55\mathrm{Kg}.m^{2},\\ \; & M_{s}=40\mathrm{Nm},M_{f}=3\mathrm{Nm},\mathrm{and}\;p_{max}=1.\\ \mathrm{with}\;\mathrm{bounds}: & 60\leq x_{1}\leq 80,\\ \; & 90\leq x_{2}\leq 110,\\ \; & 1\leq x_{3}\leq 3,\\ \; & 0\leq x_{4}\leq 1000,\\ \; & 2\leq x_{5}\leq 9. \end{array}$$

**Figure 31:**
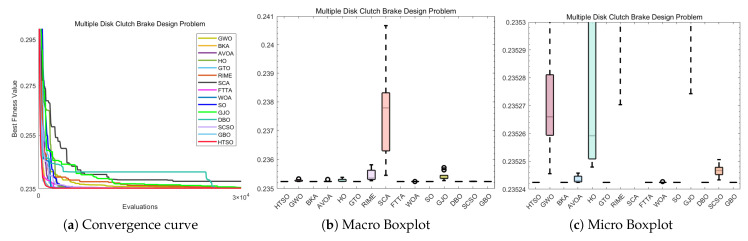
The convergence curve and boxplot of MDCB.

Table 11 presents the experimental results for the MDCB problem. The HTSO algorithm achieved both the best and average fitness values of $2.352\times 10^{-1}$ and tied for first place in both best and mean rank alongside six other algorithms (BKA, GTO, FTTA, SO, DBO, and GBO). The Wilcoxon test results indicate no statistically significant performance difference between HTSO and these six algorithms on the MDCB problem. Furthermore, Figure 31b,c display boxplots for fifteen algorithms, clearly illustrating the substantial performance gap between these seven top performers and the remaining algorithms. These findings confirm the stability and superiority of the seven algorithms on the MDCB problem.

Subsequently, the convergence curve for the MDCB problem is presented in Figure 31a, where it is evident that HTSO converges more rapidly compared to the other six algorithms. This demonstrates that, although the final results of the seven algorithms are identical, HTSO retains an irreplaceable advantage in terms of convergence speed.

### 5.7. Planetary Gear Train Design (PGTD)

For the planetary gear train design (PGTD) problem, the main optimization goal is to reduce the largest deviation found in the gear ratios within an automotive transmission system. The PGTD problem is complex, with constraints primarily involving the geometric structure and assembly conditions of the gears. Gear ratio errors affect transmission accuracy, system stability, and energy transfer efficiency. By optimizing the combination of gear teeth, the gear ratio can be made closer to the target value, helping to reduce power loss and gear wear, thereby enhancing the efficiency and durability of the entire vehicle transmission system. Planetary gears are crucial components in automotive automatic transmissions, and their design accuracy directly impacts transmission performance. Optimizing gear ratio errors not only improves shifting smoothness and response time but also extends system life and reduces maintenance costs. Therefore, this optimization problem holds significant engineering value in enhancing vehicle performance, reducing energy consumption, and advancing smart manufacturing. The PGTD problem is defined as follows:

(25) $$\begin{array}{cc} \mathrm{Minimize}: & f\left(\vec{x}\right)=\max\left|i_{k}-i_{0k}\right|,k=\left\{1,2,\ldots ,R\right\}\\ \mathrm{subject}\;\mathrm{to}: & g_{1}\left(\vec{x}\right)=m_{3}\left(N_{6}+2.5\right)-D_{max}\leq 0,\\ \; & g_{2}\left(\vec{x}\right)=m_{1}\left(N_{1}+N_{2}\right)+m_{1}\left(N_{2}+2\right)-D_{\max}\leq 0,\\ \; & g_{3}\left(\vec{x}\right)=m_{3}\left(N_{4}+N_{5}\right)+m_{3}\left(N_{5}+2\right)-D_{max}\leq 0,\\ \; & g_{4}\left(\vec{x}\right)=\left|m_{1}\left(N_{1}+N_{2}\right)-m_{3}\left(N_{6}-N_{3}\right)\right|-m_{1}-m_{3}\leq 0,\\ \; & g_{5}\left(\vec{x}\right)=-\left(N_{1}+N_{2}\right)\sin\left(\pi /p\right)+N_{2}+2+\delta_{22}\leq 0,\\ \; & g_{6}\left(\vec{x}\right)=-\left(N_{6}-N_{3}\right)\sin\left(\pi /p\right)+N_{3}+2+\delta_{33}\leq 0,\\ \; & g_{7}\left(\vec{x}\right)=-\left(N_{4}+N_{5}\right)\sin\left(\pi /p\right)+N_{5}+2+\delta_{55}\leq 0,\\ \; & g_{8}\left(\vec{x}\right)={\left(N_{3}+N_{5}+2+\delta_{35}\right)}^{2}-{\left(N_{6}-N_{3}\right)}^{2}-{\left(N_{4}+N_{5}\right)}^{2}\\ \; & +2\left(N_{6}-N_{3}\right)\left(N_{4}+N_{5}\right)\cos\left(\frac{2\pi }{p}-\beta \right)\leq 0,\\ \; & g_{9}\left(\vec{x}\right)=N_{4}-N_{6}+2N_{5}+2\delta_{56}+4\leq 0,\\ \; & g_{10}\left(\vec{x}\right)=2N_{3}-N_{6}+N_{4}+2\delta_{34}+4\leq 0,\\ \; & h_{1}\left(\vec{x}\right)=\frac{N_{6}-N_{4}}{p}=\mathrm{integer}.\\ \mathrm{where}: & i_{1}=\frac{N_{6}}{N_{4}},i_{01}=3.11,i_{2}=\frac{N_{6}\left(N_{1}N_{3}+N_{2}N_{4}\right)}{N_{1}N_{3}\left(N_{6}-N_{4}\right)},\;i_{0R}=-3.11,\\ \; & I_{R}=-\frac{N_{2}N_{6}}{N_{1}N_{3}},i_{02}=1.84,\;\vec{x}=\left(p,N_{6},N_{5},N_{4},N_{3},N_{2},N_{1},m_{2},m_{1}\right)\\ \; & \delta_{22}=\delta_{33}=\delta_{55}=\delta_{35}=\delta_{56}=0.5.\\ \; & \beta =\frac{\cos^{-1}\left({\left(N_{4}+N_{5}\right)}^{2}+{\left(N_{6}-N_{3}\right)}^{2}-{\left(N_{3}+N_{5}\right)}^{2}\right)}{2\left(N_{6}-N_{3}\right)\left(N_{4}+N_{5}\right)},D_{max}=220.\\ \mathrm{with}\;\mathrm{bounds}: & p=(3,4,5),\\ \; & m_{1}=\left(1.75,2.0,2.25,2.5,2.75,3.0\right),\\ \; & m_{3}=\left(1.75,2.0,2.25,2.5,2.75,3.0\right),\\ \; & 17\leq N_{1}\leq 96,\;14\leq N_{2}\leq 54,\;14\leq N_{3}\leq 51\\ \; & 17\leq N_{4}\leq 46,\;14\leq N_{5}\leq 51,\;48\leq N_{6}\leq 124,\\ \; & and\;N_{i}=integer. \end{array}$$

Table 12 presents the experimental results for the PGTD problem, where HTSO achieves an average optimization result of $5.299\times 10^{-1}$, ranking first. The best optimization result for HTSO is $5.263\times 10^{-1}$, which ranks second, just behind RIME’s $5.260\times 10^{-1}$. However, HTSO’s standard deviation of $4.198\times 10^{-3}$ is the smallest among the 15 algorithms, indicating superior stability compared to RIME. According to the Wilcoxon test results, there is no significant difference in performance between HTSO and either RIME or SO, whereas HTSO demonstrates markedly superior outcomes compared to the remaining twelve algorithms.

**Figure 32:**
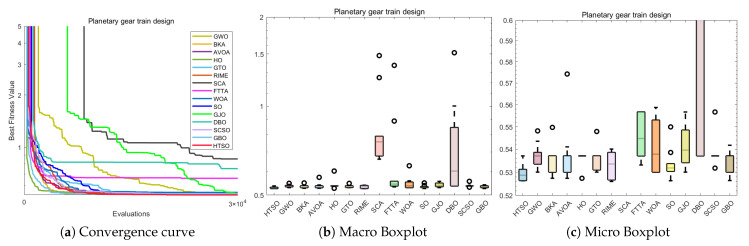
The convergence curve and boxplot of PGTD.

Figure 32a illustrates the convergence speed, while Figure 32b and Figure 32c present the macro- and micro-level boxplots, respectively. In Figure 32b, only HTSO, RIME, GJO, and GBO exhibit no outliers. Although the Wilcoxon test shows no significant performance difference between SO and HTSO, SO displays outliers in the boxplot, indicating that SO is less stable than HTSO. In Figure 32c, SCA is omitted because its convergence precision is too low to render a box. Among the remaining 14 algorithms, HO and SCSO yield the smallest boxes—suggesting relatively high stability—but Table 12 reveals their convergence precision is poor, so their overall performance falls short of HTSO.

### 5.8. Gas Transmission Compressor Design (GTCD)

In mechanical engineering, optimizing the design of gas transmission compressors is a frequent and well-recognized challenge. In practice, gas transmission compressors are widely used in industries such as natural gas transportation, petrochemicals, and air conditioning and cooling systems. Optimizing the design of these compressors can enhance energy efficiency, reduce operational costs, and minimize environmental impact. As global energy demand rises and interest in green technologies grows, optimizing gas compressor design becomes increasingly significant from both economic and environmental perspectives. The mathematical expression for GTCD is as follows:

(26) $$\begin{array}{cc} \mathrm{Minimize}: & f\left(\vec{x}\right)=8.61\times 10^{5}x_{1}^{1/2}x_{2}x_{3}^{-2/3}x_{4}^{-1/2}+3.69\times 10^{4}x_{3}+7.72\\ \; & \;\times 10^{8}x_{1}^{-1}x_{2}^{0.219}-765.43\times 10^{6}x_{1}^{-1}\\ \mathrm{subject}\;\mathrm{to}: & x_{4}x_{2}^{-2}+x_{2}^{-2}-1\leq 0.\\ \mathrm{with}\;\mathrm{bounds}: & 20\leq x_{1}\leq 50,\\ \; & 1\leq x_{2}\leq 10,\\ \; & 20\leq x_{3}\leq 50,\\ \; & 0.1\leq x_{4}\leq 60. \end{array}$$

Table 13 displays the experimental findings obtained for the GTCD problem. HTSO achieved the lowest mean and best values (2,963,417.47 each) with a remarkably low standard deviation of $4.910\times 10^{-10}$, indicating exceptional stability. According to Wilcoxon test, HTSO’s performance on the GTCD problem did not differ significantly from that of GTO and GBO, and also showed no significant difference compared to FTTA. Furthermore, HTSO significantly outperformed the other eleven competing algorithms.

**Figure 33:**
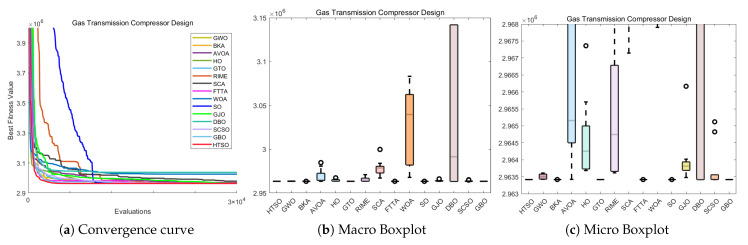
The convergence curve and boxplot of GTCD.

Figure 33a illustrates that HTSO quickly converges to the global optimum. The macro-level boxplot in Figure 33b shows that DBO and WOA perform poorly on the GTCD problem, while AVOA and SCA also demonstrate subpar performance. The micro-level boxplot in Figure 33c provides more detailed information, revealing that only HTSO, GTO, and GBO exhibit both compact boxplots and the absence of outliers, whereas FTTA displays outliers. These results confirm that HTSO, GTO, and GBO outperform the other twelve algorithms in terms of stability and convergence accuracy.

### 5.9. Rolling Element Bearing Design (REBD)

Optimizing the design of rolling element bearings focuses on increasing dynamic load capacity, meeting required load-bearing and service-life criteria, and ultimately improving the efficiency and overall performance of mechanical systems. Optimization outcomes directly influence critical performance metrics such as contact-force distribution, stress concentration, vibration behavior, and heat generation. Appropriate design can reduce contact stress and friction, extend bearing life, prevent premature fatigue failure, and improve system stability and reliability. Optimization is particularly crucial under high-speed or heavy-load conditions for minimizing mechanical energy loss and enhancing motion accuracy. In practical applications, rolling element bearings are widely used in automotive, aerospace, and industrial machinery. Effective optimization not only improves operational efficiency and stability but also reduces energy consumption and maintenance costs, thus supporting the development of high-performance machinery and energy-saving technologies. The REBD problem is characterized by five key design variables: pitch diameter, ball diameter, curvature coefficients for both the outer and inner raceways, and the total quantity of balls. Additionally, it incorporates nine nonlinear constraints, which originate from both manufacturing limitations and kinematic requirements (refer to Equation (27)).

(27) $$\begin{array}{cc} \mathrm{Minimize}: & f\left(\vec{x}\right)=\left\{\begin{array}{cc} f_{c}Z^{2/3}D_{b}^{1.8} & ,if\;D_{b}\leq 25.4\mathrm{mm}\\ 3.647f_{c}Z^{2/3}D_{b}^{1.4} & ,\mathrm{otherwise} \end{array}\right.\\ \mathrm{subject}\;\mathrm{to}: & g_{1}\left(\vec{x}\right)=Z-\frac{\phi_{0}}{2\sin^{-1}\left(D_{b}/D_{m}\right)}-1\leq 0,\\ \; & g_{2}\left(\vec{x}\right)=K_{D\min}\left(D-d\right)-2D_{b}\leq 0.\\ \; & g_{3}\left(\vec{x}\right)=2D_{b}-K_{Dmax}\left(D-d\right)\leq 0,\\ \; & g_{4}\left(\vec{x}\right)=D_{b}-\zeta B_{w}\leq 0,\\ \; & g_{5}\left(\vec{x}\right)=0.5\left(D+d\right)-D_{m}\leq 0,\\ \; & g_{6}\left(\vec{x}\right)=D_{m}-\left(0.5+e\right)\left(D+d\right)\leq 0,\\ \; & g_{7}\left(\vec{x}\right)=\epsilon D_{b}-0.5\left(D-D_{m}-D_{b}\right)\leq 0,\\ \; & g_{8}\left(\vec{x}\right)=0.515-f_{i}\leq 0,\\ \; & g_{9}\left(\vec{x}\right)=0.515-f_{0}\leq 0.\\ \mathrm{where}: & f_{c}=37.91{\left\{1+{\left\{1.04{\left(\frac{1-\gamma }{1+\gamma }\right)}^{1.72}{\left(\frac{f_{i}\left(2f_{0}-1\right)}{f_{0}\left(2f_{1}-1\right)}\right)}^{0.41}\right\}}^{10/3}\right\}}^{-0.3}.\\ \; & \gamma =\frac{D_{b}\cos\left(a\right)}{D_{m}},f_{i}=\frac{r_{i}}{D_{b}},f_{0}=\frac{r_{0}}{D_{b}},\\ \; & \phi_{0}=2\pi -2\cos^{-1}\\ \; & \left(\frac{{\left\{\left(D-d\right)/2-3\left(T/4\right)\right\}}^{2}+{\left(D/2-\left(T/4\right)-D_{b}\right\}}^{2}-{\left\{d/2+\left(T/4\right)\right\}}^{2}}{2\left\{\left(D-d\right)/2-3\left(T/4\right)\right\}\left\{D/2-\left(T/4\right)-D_{b}\right\}}\right)\\ \; & T=D-d-2D_{h},D=160,d=90,B_{w}=30.\\ \mathrm{with}\;\mathrm{bounds}: & 0.5\left(D+d\right)\leq D_{m}\leq 0.6\left(D+d\right),\\ \; & 0.15\left(D-d\right)\leq D_{b}\leq 0.45\left(D-d\right),\\ \; & 4\leq Z\leq 50,\\ \; & 0.515\leq f_{i}\leq 0.6,\\ \; & 0.515\leq f_{0}\leq 0.6,\\ \; & 0.4\leq K_{Dmin}\leq 0.5,\\ \; & 0.6\leq K_{Dmax}\leq 0.7,\\ \; & 0.3\leq \epsilon \leq 0.4,\\ \; & 0.02\leq e\leq 0.1,\\ \; & 0.6\leq \zeta \leq 0.85. \end{array}$$

Table 14 presents the REBD experimental results. HTSO attained both a best and mean value of $1.461\times 10^{4}$, tying for first place with SO and GBO. The performance differences among these three algorithms on the REBD problem are negligible, such that the Wilcoxon test cannot distinguish them. Moreover, although HO, FTTA, and DBO exhibit greater variability—reflected in mean values lower than those of the top three—they nonetheless achieved the same best value.

**Figure 34:**
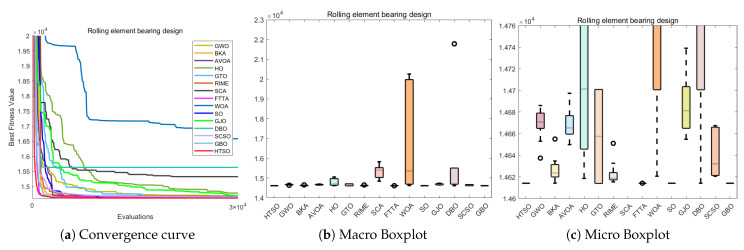
The convergence curve and boxplot of REBD.

From the macro-level boxplot (Figure 34b), it is evident that the box for WOA is significantly wider than those of the other algorithms, with DBO exhibiting the largest outlier. While SCA and HO do not show any outliers, their boxes are relatively wide, indicating higher fitness values. These four algorithms perform poorly on the REBD problem. An analysis of Figure 34c reveals that only HTSO, SO, and GBO demonstrate stable performance, while FTTA presents an outlier, slightly reducing its overall performance compared to the top three algorithms. Figure 34a illustrates the convergence curve for the REBD problem, clearly showing that among the 15 algorithms, HTSO converges the fastest, achieving the highest convergence speed.

### 5.10. Tension/Compression String Design Problem (Case 2) (TCPD (Case 2))

The objective of the Tension/Compression Spring Design Problem (Case 2) is to minimize the volume of steel wire required to manufacture a helical compression spring, thereby reducing both material consumption and cost. Case 2 of the TCPD problem involves three decision variables—a continuous variable (spring outer diameter), an integer variable (number of coils), and a discrete variable (wire diameter)—and is subject to eight nonlinear constraints. Compared to Case 1, Case 2 of the TCPD is substantially more complex and thus presents a greater optimization challenge. The corresponding mathematical representation is outlined below:

(28) $$\begin{array}{cc} \mathrm{Minimize}: & f\left(\vec{x}\right)=\frac{\pi^{2}x_{2}x_{3}^{2}\left(x_{1}+2\right)}{4}\\ \mathrm{subject}\;\mathrm{to}: & g_{1}\left(\vec{x}\right)=\frac{8000C_{f}x_{2}}{\pi x_{3}^{3}}-189,000\leq 0,\\ \; & g_{2}\left(\vec{x}\right)=l_{f}-14\leq 0.\\ \; & g_{3}\left(\vec{x}\right)=0.2-x_{3}\leq 0,\\ \; & g_{4}\left(\vec{x}\right)=x_{2}-3\leq 0,\\ \; & g_{5}\left(\vec{x}\right)=3-\frac{x_{2}}{x_{3}}\leq 0,\\ \; & g_{6}\left(\vec{x}\right)=\sigma_{p}-6\leq 0,\\ \; & g_{7}\left(\vec{x}\right)=\sigma_{p}+\frac{700}{K}+1.05\left(x_{1}+2\right)x_{3}-l_{f}\leq 0,\\ \; & g_{8}\left(\vec{x}\right)=1.25-\frac{700}{K}\leq 0,\\ \mathrm{where}: & C_{f}=\frac{4\frac{x_{2}}{x_{3}}-1}{4\frac{x_{2}}{x_{3}}-4}+\frac{0.615x_{3}}{x_{2}},K=\frac{11.5\times 10^{6}x_{3}^{4}}{8x_{1}x_{2}^{3}},\sigma_{p}=\frac{300}{K},\\ \; & l_{f}=\frac{1000}{K}+1.05\left(x_{1}+2\right)x_{3}.\\ \mathrm{with}\;\mathrm{bounds}: & 1\leq x_{1}\left(integer\right)\leq 70,\\ \; & x_{3}\left(discreate\right)\in \{0.009,0.0095,0.0104,0.0118,0.0128,\\ \; & 0.0132,0.014,0.015,0.0162,0.0173,0.018,0.020,0.023,\\ \; & 0.025,0.028,0.032,0.035,0.041,0.047,0.054,0.063,0.072,\\ \; & 0.080,0.092,0.0105,0.120,0.135,0.148,0.162,0.177,0.192,\\ \; & 0.207,0.225,0.244,0.263,0.283,0.307,0.0331,0.362,0.394,\\ \; & 0.4375,0.500\}\\ \; & 0.6\leq x_{2}\left(continuous\right)\leq 3. \end{array}$$

Table 15 presents the experimental results for the TCPD (Case 2). In this case, HTSO achieves the lowest Mean and Best values, and the Wilcoxon test results demonstrate that HTSO significantly outperforms all other algorithms, exhibiting superior performance.

**Figure 35:**
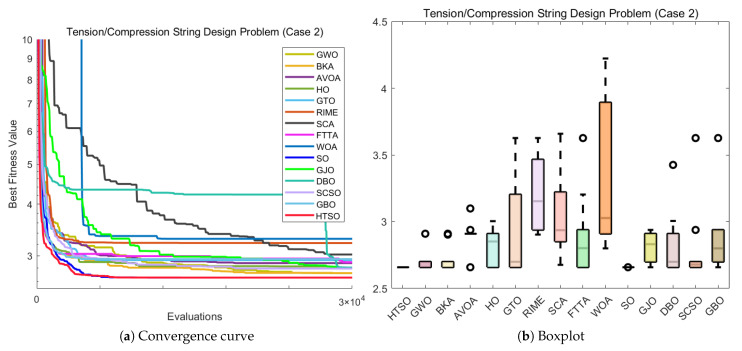
The convergence curve and boxplot of TCPD (case 2).

The boxplot in Figure 35b clearly illustrates that HTSO is the only algorithm with a narrow box and no outliers.

As illustrated in Figure 35a, the convergence curve indicates that HTSO achieves the most rapid convergence rate compared to the other algorithms assessed. Altogether, these findings underscore the superior effectiveness of HTSO when addressing the TCPD (Case 2) problem.

### 5.11. Weight Minimization of a Speed Reducer (WMSR)

The Weight Minimization of a Speed Reducer (WMSR) problem focuses on reducing the mass of a compact aero-engine gearbox, subject to various mechanical and structural limitations. The optimization considers seven design variables—gear width, number of teeth, and shaft length and diameter, among others—and is governed by eleven inequality constraints. Owing to the problem’s high complexity and narrow feasible region, the optimization is particularly challenging. Proper dimensioning of components not only reduces overall weight but also mitigates vibration, improves transmission efficiency, and enhances operational stability. Weight reduction of the speed reducer is critical in aerospace, automotive, and related industries, as it improves fuel efficiency, reduces emissions, and elevates overall equipment performance. Therefore, optimizing this problem is essential for advancing engineering design and promoting green manufacturing. Equation (29) provides the mathematical formulation for the WMSR problem.

(29) $$\begin{array}{cc} \mathrm{Minimize}: & f\left(\vec{x}\right)=0.7854x_{2}^{2}x_{1}\left(14.9334x_{3}-43.0934+3.3333x_{3}^{2}\right)\\ \; & +0.7854\left(x_{5}x_{7}^{2}+x_{4}x_{6}^{2}\right)-1.508x_{1}\left(x_{7}^{2}+x_{6}^{2}\right)+7.477\left(x_{7}^{3}+x_{6}^{3}\right)\\ \mathrm{subject}\;\mathrm{to}: & g_{1}\left(\vec{x}\right)=-x_{1}x_{2}^{2}x_{3}+27\leq 0,\\ \; & g_{2}\left(\vec{x}\right)=-x_{1}x_{2}^{2}x_{3}^{2}+397.5\leq 0,\\ \; & g_{3}\left(\vec{x}\right)=-x_{2}x_{6}^{4}x_{3}x_{4}^{-3}+1.93\leq 0,\\ \; & g_{4}\left(\vec{x}\right)=-x_{2}x_{7}^{4}x_{3}x_{5}^{-3}+1.93\leq 0.\\ \; & g_{5}\left(\vec{x}\right)=10x_{6}^{-3}\sqrt{16.91\times 10^{6}+{\left(745x_{4}x_{2}^{-1}x_{3}^{-1}\right)}^{2}}-1100\leq 0,\\ \; & g_{6}\left(\vec{x}\right)=10x_{7}^{-3}\sqrt{157.5\times 10^{6}+{\left(745x_{5}x_{2}^{-1}x_{3}^{-1}\right)}^{2}}-850\leq 0,\\ \; & g_{7}\left(\vec{x}\right)=x_{2}x_{3}-40\leq 0,\\ \; & g_{8}\left(\vec{x}\right)=-x_{1}x_{2}^{-1}+5\leq 0,\\ \; & g_{9}\left(\vec{x}\right)=x_{1}x_{2}^{-1}-12\leq 0,\\ \; & g_{10}\left(\vec{x}\right)=1.5x_{6}-x_{4}+1.9\leq 0,\\ \; & g_{11}\left(\vec{x}\right)=1.1x_{7}-x_{5}+1.9\leq 0.\\ \mathrm{with}\;\mathrm{bounds}: & 2.6\leq x_{1}\leq 3.6,0.7\leq x_{2}\leq 0.8,17\leq x_{3}\leq 28,\\ \; & 7.3\leq x_{4},x_{5}\leq 8.3,2.9\leq x_{6}\leq 3.9,5\leq x_{7}\leq 5.5. \end{array}$$

**Figure 36:**
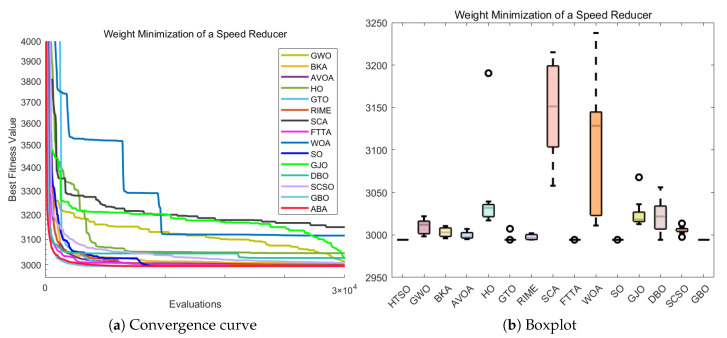
The convergence curve and boxplot of WMSR.

Table 16 presents the experimental results for the Weight Minimization of a Speed Reducer (WMSR) problem. The best value achieved by HTSO was 2994.2342, which is tied for first place with GTO, FTTA, SO, DBO, and GBO. The mean value obtained was also 2994.2342, again tied for first place with GBO. Results from the Wilcoxon test indicate that the performance of HTSO is nearly identical to that of GBO, with no significant performance difference when compared to FTTA and GTO, and it outperforms the other 11 algorithms. The boxplot (Figure 36b) shows that the boxes for GWO, BKA, AVOA, HO, RIME, SCA, WOA, GJO, DBO, and SCSO are excessively wide, and that HO, GTO, FTTA, SO, GJO, and SCSO display outliers, indicating lower stability for these algorithms. In contrast, HTSO and GBO exhibit both narrow boxes and no outliers, demonstrating their superior stability in solving the WMSR problem. Furthermore, the convergence curve (Figure 36b) clearly shows that HTSO achieves the fastest convergence speed and accuracy, further validating its strong stability and effectiveness in addressing the WMSR problem. This exceptional performance specifically highlights the algorithm’s ability to handle narrow feasible regions, distinguishing it as a robust tool for complex engineering applications.

### 5.12. The 10-Bar Truss Design Problem (10-BTD)

Compared to TBTD, 10-BTD is more representative of real-world engineering applications, involving 10 variables and three constraints. It serves as an extension and refinement of the TBTD model, while also presenting a higher level of optimization difficulty. The expression for 10-BTD is as follows:

(30) $$\begin{array}{cc} \mathrm{Minimize}: & f\left(\vec{x}\right)=\sum_{i=1}^{10}L_{i}\left(x_{i}\right)\rho_{i}A_{i}\\ \mathrm{subject}\;\mathrm{to}: & g_{1}\left(\vec{x}\right)=\frac{7}{\omega_{1}\left(\vec{x}\right)}-1\leq 0,\\ \; & g_{2}\left(\vec{x}\right)=\frac{15}{\omega_{2}\left(\vec{x}\right)}-1\leq 0,\\ \; & g_{3}\left(\vec{x}\right)=\frac{20}{\omega_{3}\left(\vec{x}\right)}-1\leq 0.\\ \mathrm{with}\;\mathrm{bounds}: & 6.45\times 10^{-5}\leq A_{i}\leq 5\times 10^{-3},i=1,2,\ldots ,10.\\ \mathrm{where}: & \vec{x}=\left\{A_{1},A_{2},\ldots ,A_{10}\right\},\rho =2770. \end{array}$$

**Figure 37:**
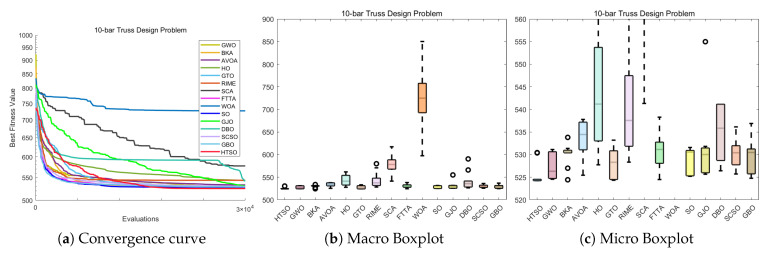
The convergence curve and boxplot of 10-BTD.

The experimental results presented in Table 17 show that HTSO achieved a mean value of 525.5231 and a best value of 524.2426. HTSO demonstrates strong performance on the 10-BTD problem, as it ranks first in both the mean value and best value. Moreover, according to the Wilcoxon test, HTSO demonstrates superior performance compared to the other algorithms. Both the macro boxplot (Figure 37b) and the micro boxplot (Figure 37c) reveal that, although HTSO displays one outlier, it consistently finds better solutions than other algorithms, with the best value being superior in most cases. Moreover, HTSO has the smallest standard deviation (Std). These results collectively confirm the outstanding performance of HTSO on the 10-BTD problem.

The convergence curve shown in Figure 37a demonstrates that although HTSO does not converge as quickly as other algorithms on the 10-BTD problem, it consistently escapes local optima when other algorithms become trapped, continuously finding better solutions. As a result, HTSO eventually outperforms all other algorithms, achieving the first rank in terms of the mean value. This further confirms HTSO’s exceptional ability to escape local optima.

### 5.13. Summary of the COPs

**Figure 38:**
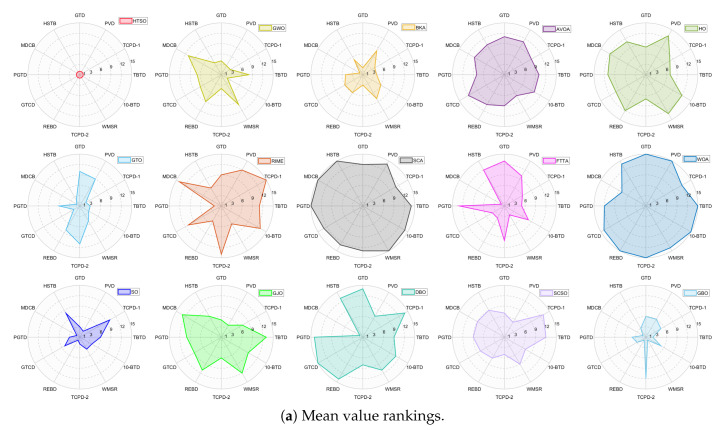

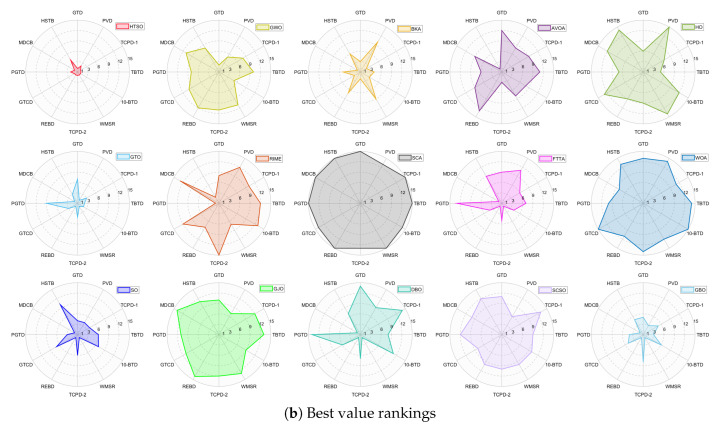
Comparison chart of the ranking of COPs.

In this section, we apply 15 advanced algorithms, including HTSO, to 12 classic mechanical engineering problems and perform a comparative performance analysis. Among the 168 Wilcoxon tests conducted across 12 COPs and compared with 14 other algorithms, 17 tests showed no significant difference, accounting for 10.11% of the total comparisons. The remaining 151 comparisons were all superior to the competing algorithms, representing 89.89% of the total comparisons. HTSO did not lose to any of the comparison algorithms. To visually highlight the performance advantages of the HTSO algorithm, we present radar charts showing the mean rank and best rank of the 15 algorithms across the 12 mechanical engineering COPs. The radar chart for the mean rank (Figure 38a) shows that HTSO consistently achieved the first rank in all 12 COPs. The radar chart for the best rank (Figure 38b) reveals that HTSO secured nine first-place finishes, two second-place finishes, and one fourth-place finish. Although HTSO did not achieve the best value first place in all problems, its radar chart is clearly smaller than those of the other 14 algorithms. This strongly demonstrates HTSO’s robust, stable performance, broad applicability, and significant potential in solving mechanical engineering COPs, providing a solid foundation for its application in practical engineering fields.

## 6. 3D Trajectory Planning for UAVs

With the widespread use of unmanned aerial vehicles (UAVs) in both civilian and military domains, their significance and convenience in various tasks have become increasingly apparent [146]. Path planning and design represent a fundamental aspect of UAV autonomous control systems. This process involves addressing a challenging optimization problem with multiple constraints, namely determining a secure and dependable route that leads from the origin to the intended destination while adhering to all relevant limitations [147]. In recent years, the increasing variety of UAV models and their expanding range of application environments have made path planning an important focus of research. 3D UAV path planning is not only a technical challenge but also a resource-optimization problem. In practical applications—especially for prolonged flights in complex environments—it is essential to conserve energy and minimize flight time. From a resource-optimization perspective, effective path planning ensures successful mission completion while significantly enhancing flight efficiency, extending battery endurance, and reducing energy consumption. This capability is critical for extended UAV operations, particularly in scenarios requiring large-area patrol or long-distance flights, such as military reconnaissance and logistics delivery. By optimizing flight trajectories, avoiding unnecessary obstacles, and minimizing detours, UAVs can markedly improve operational efficiency under constrained energy resources.

In conclusion, with the continuous advancement of UAV technology, research on path planning has become increasingly important. By applying meta-heuristic algorithms to 3D UAV path planning, it is possible to address flight challenges in real-world environments, enabling more efficient and safer missions. This holds significant practical implications for the widespread use of UAVs across various application scenarios [148].

### 6.1. UAV 3D Path Planning Modeling

This study develops a mountainous terrain model to simulate the 3D path planning of UAVs. In mountainous regions, the flight routes of UAVs are mainly shaped by elements such as towering peaks, challenging weather conditions, and restricted airspaces. Typically, to guarantee safe operation, UAVs must plan their paths to avoid these hazardous zones. Concerning UAV path planning in such environments, this paper thoroughly considers multiple factors, including mountain summits, meteorological hazards, and no-fly areas, and develops an appropriate path-planning model. The mathematical model employed to represent the terrain and obstacles is outlined in Equation (31).

(31) $$z=\sin\left(y+1\right)+\sin\left(x\right)+\cos\left(x^{2}+y^{2}\right)+2\times \cos\left(y\right)+\sin\left(x^{2}+y^{2}\right)$$

When operating UAVs, certain trajectory limitations must be met. These limitations mainly involve aspects like the total path length, flight altitude and maximum allowable turning angle.

- Path Length: Generally, the main objective of UAV operations is to optimize time efficiency and minimize costs, all while maintaining safety. Consequently, the trajectory length is a key factor in path planning. This constraint is mathematically represented in Equation (32).
  (32) $$PL=\sum_{m=1}^{g-1}{\left\|\left(x_{m+1},y_{m+1},z_{m+1}\right)-\left(x_{m},y_{m},z_{m}\right)\right\|}_{2}$$
- Flight Altitude: The operational altitude of a UAV plays a crucial role in influencing its control system and ensuring flight safety. Equation (33) presents the mathematical formulation of this constraint.
  (33) $$FA=\sqrt{\sum_{m=1}^{g}{\left(z_{m}-\frac{1}{n}\sum_{k=1}^{g}z_{m}\right)}^{2}}$$
- Maximum Turning Angle: The UAV’s turning angle must stay within the specified maximum limit. This constraint on the maximum turning angle is represented as:
  (34) $$MA=\sum_{m=1}^{g-2}\left(\arccos\left(\frac{\varphi_{m+1}\times \varphi_{m}}{|\varphi_{m+1}\left|\times \right|\varphi_{m}|}\right)\right)$$
  Here, $\varphi_{m}$ represents $x_{m+1}-x_{m},y_{m+1}-y_{m},z_{m+1}-z_{m}$.

Under the constraints mentioned above, we can derive the objective function for the 3D UAV path planning problem, as shown in Equation (35). The variables $w_{i}wherei=\left(1,2,3\right)$ represent the weighting factors. The limitations on these coefficients are outlined in Equation (36). Adjusting these weight values allows for the modification of each factor’s influence on the trajectory.

(35) $$min\;P=w_{1}\times PL+w_{2}\times FA+w_{3}\times MA$$

(36) $$\left\{\begin{array}{c} w_{i}\geq 0\\ \sum_{i=1}^{3}w_{i}=1 \end{array}\right.$$

### 6.2. Experiment on 3D Path Planning for UAVs

To validate the effectiveness of the HTSO algorithm in solving the 3D UAV path planning problem, this section compares HTSO with 14 other algorithms under seven distinct mountainous terrain models. The algorithms compared include GJO [38], DBO [43], FTTA [103], BKA [46], WOA [30], HHO [31], SMA [34], SSA [32], SCA [78], SCSO [45], MFO [29], GWO [28], SO [39], and RIME [74], with their respective hyperparameters listed in Table 2. In the models, the parameters $w_{1}$, $w_{2}$, and $w_{3}$ are set to 0.4, 0.4, and 0.2, respectively, with the start and end points set at (0,0,20) and (200,200,30), respectively. The population size is set to 30, and the maximum number of evaluations is 30,000. If a valid path is not found, the fitness value is considered anomalous. Each algorithm is independently run 10 times, and if any run fails to find a valid path, the mean, standard deviation, and worst value are considered anomalous. A cubic spline interpolation is applied three times to generate a path, and the average, standard deviation, best value, and worst value of the 10 runs are computed. The 14 comparison algorithms are then subjected to a Wilcoxon rank-sum test at a significance level of p=0.05. Finally, the results are presented in terms of the Friedman average ranking line chart and the convergence curves of the optimal solutions for each algorithm.

#### 6.2.1. Model of Mountain No. 1

**Figure 39:**
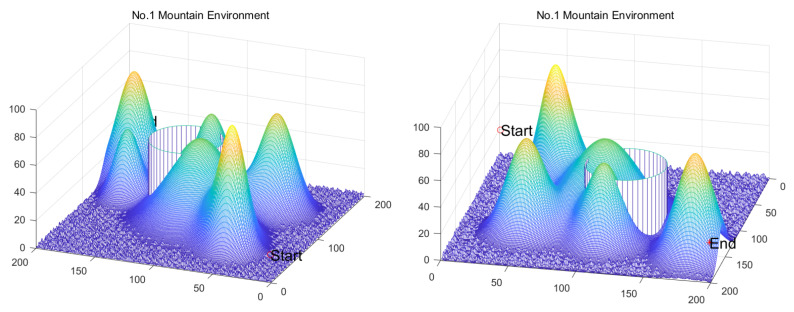
Mountain No. 1 Environment Simulation.

The environmental map model of Mountain No. 1 is shown in Figure 39. This model contains seven peaks, with their respective coordinates and elevations listed as follows: (60, 60, 50), (100, 100, 60), (180, 160, 80), (50, 140, 70), (50, 45, 65), (110, 150, 54), and (170, 120, 50).

**Figure 40:**
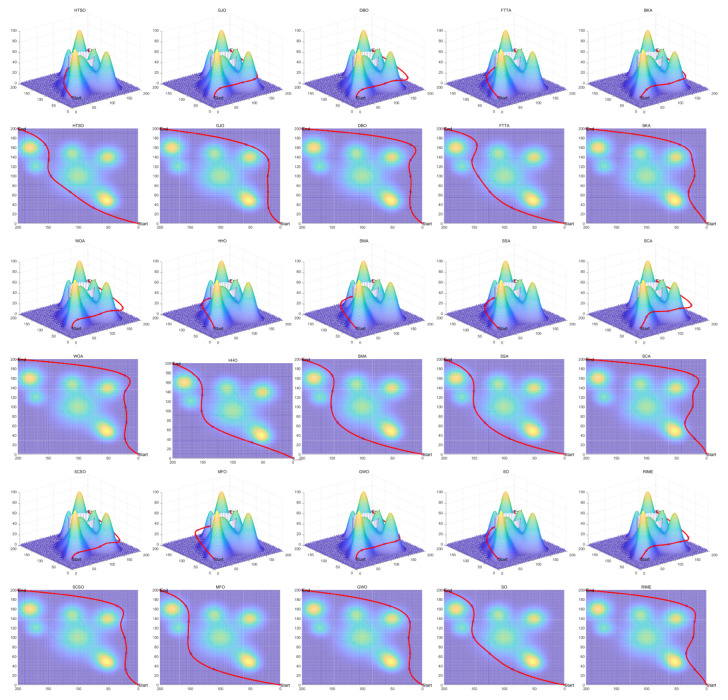
The generated UAV paths from fifteen algorithms of Mountain No. 1.

**Figure 41:**
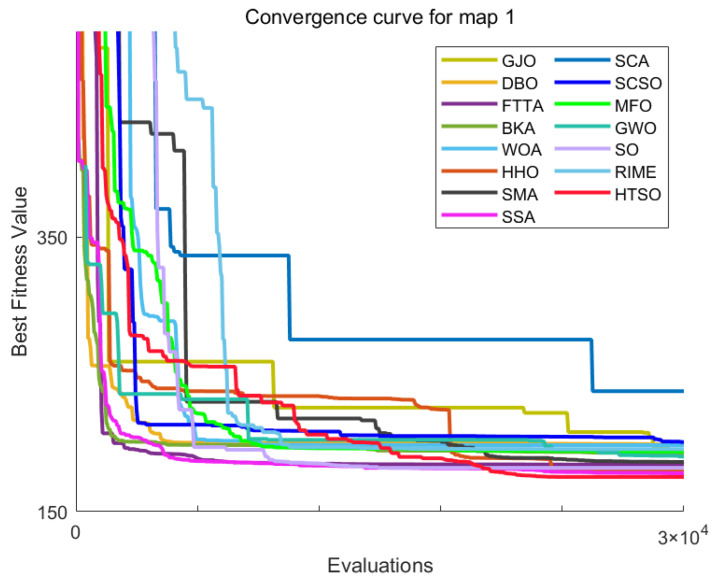
Convergence curve for Mountain No. 1.

The experimental results for Map 1 are presented in Table 18. Figure 40 illustrates the optimal feasible paths—derived by fifteen algorithms—in both 3D and 2D. Figure 41 shows the convergence curves for all fifteen algorithms. From these experiments, HTSO achieved the best solution of 175.2654 and an average result of 190.8603. On this model, HTSO ranked first among all algorithms in worst-case, best-case, standard deviation, and mean performance, demonstrating superior overall performance. Wilcoxon tests indicate that HTSO’s results do not differ significantly from FTTA, SMA, or SSA, but are significantly better than those of the other eleven algorithms. Moreover, the anomalous average results of HHO, SCSO, and GWO reveal that these methods occasionally failed to find a feasible path.

#### 6.2.2. Model of Mountain No. 2

**Figure 42:**
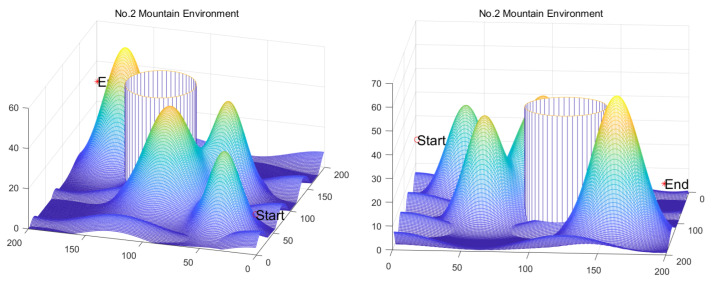
Mountain No. 2 environment simulation.

Figure 42 presents the model of Mountain No. 2. This mountain is relatively simple, consisting of only four peaks. The coordinates and heights of these peaks are (40, 40, 40), (100, 80, 50), (160, 150, 60), and (60, 100, 45), respectively.

**Figure 43:**
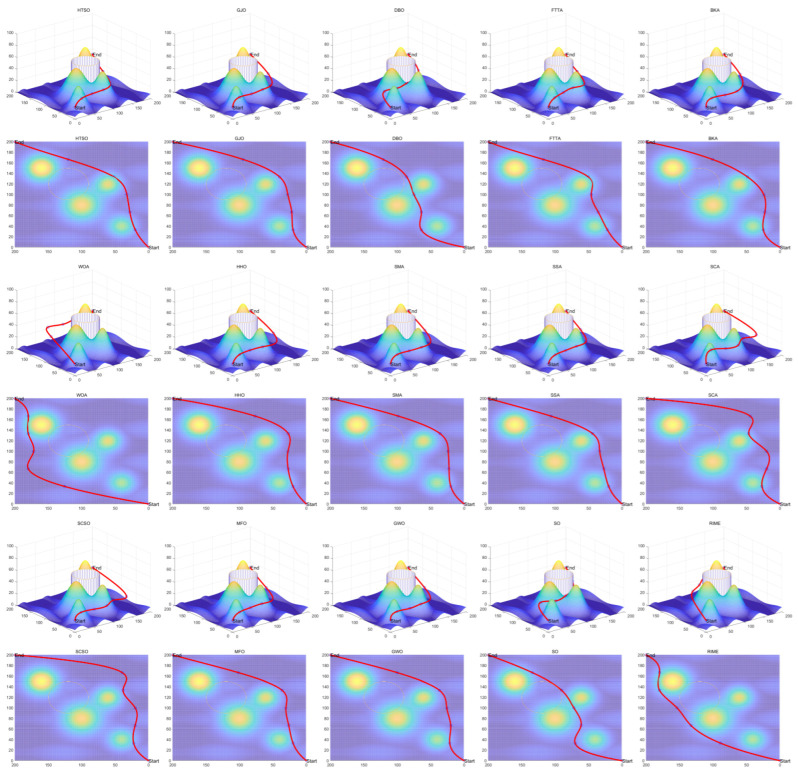
The generated UAV paths from fifteen algorithms of Mountain No. 2.

**Figure 44:**
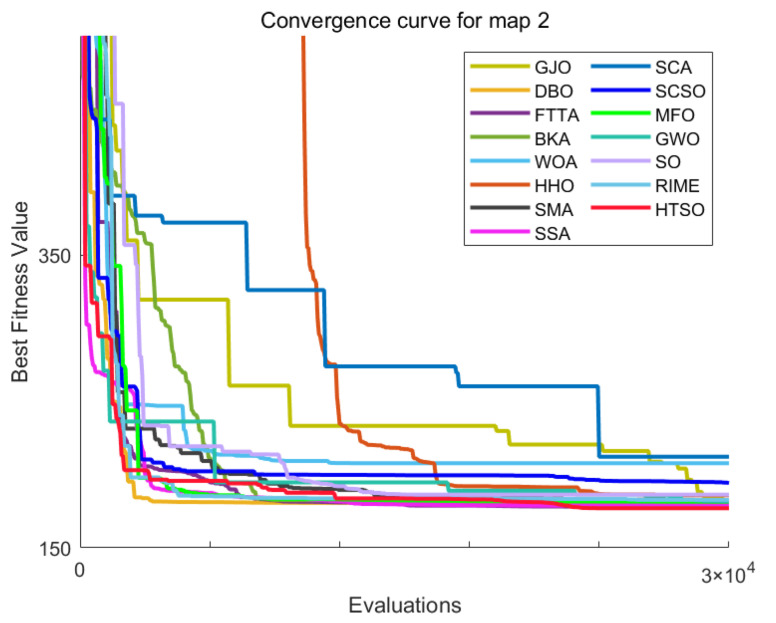
Convergence curve for Mountain No. 2.

Figure 43 illustrates both the 3D and 2D representations of the optimal paths obtained by the 15 algorithms, while Table 19 presents the corresponding experimental results. It can be observed that HTSO achieves the best solution of 177.0526 and an average result of 180.7923; moreover, it ranks first across all four evaluation metrics. The Wilcoxon test indicates that HTSO significantly outperforms all other algorithms. Figure 44 shows the convergence curves of the 15 algorithms, where HTSO is observed to converge rapidly in the early stages of iteration and is still able to discover new local optima in later stages, demonstrating both strong convergence speed and high population diversity.

#### 6.2.3. Model of Mountain No. 3

**Figure 45:**
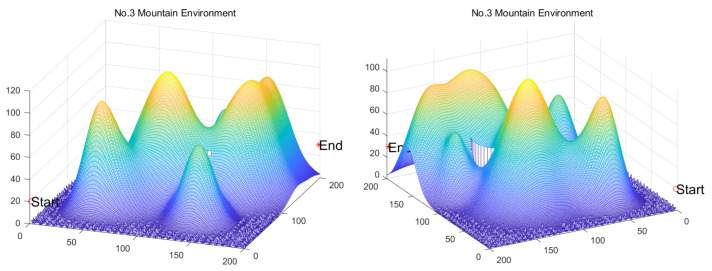
Mountain No. 3 environment simulation.

Mountain No. 3 contains nine peaks, located at (60, 60, 50), (100, 100, 60), (180, 160, 80), (50, 140, 70), (50, 45, 65), (110, 150, 54), (170, 120, 50), (120, 80, 75), and (140, 160, 85). The model is illustrated in Figure 45.

**Figure 46:**
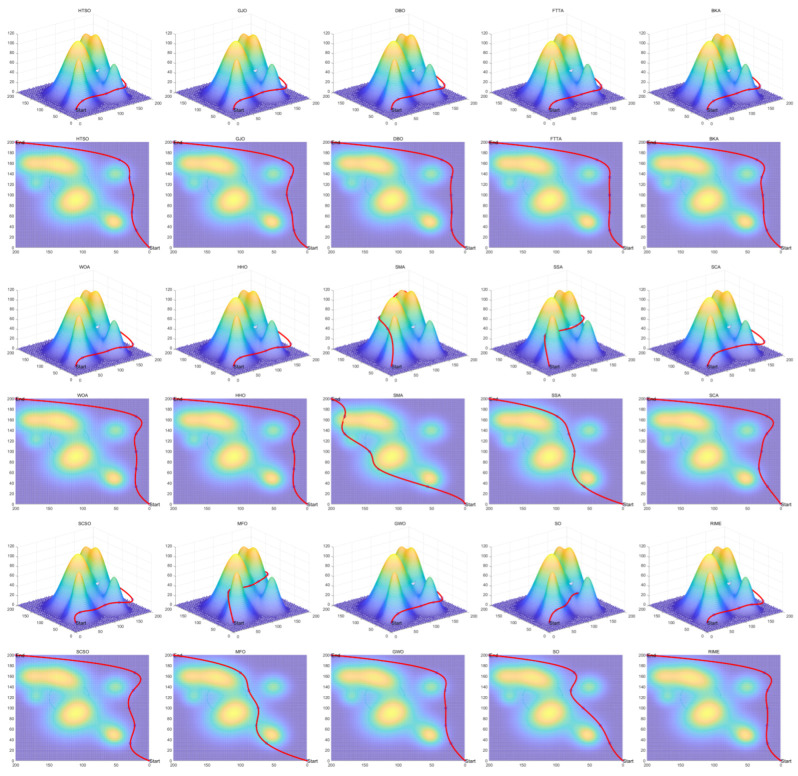
The generated UAV paths from fifteen algorithms of Mountain No. 3.

**Figure 47:**
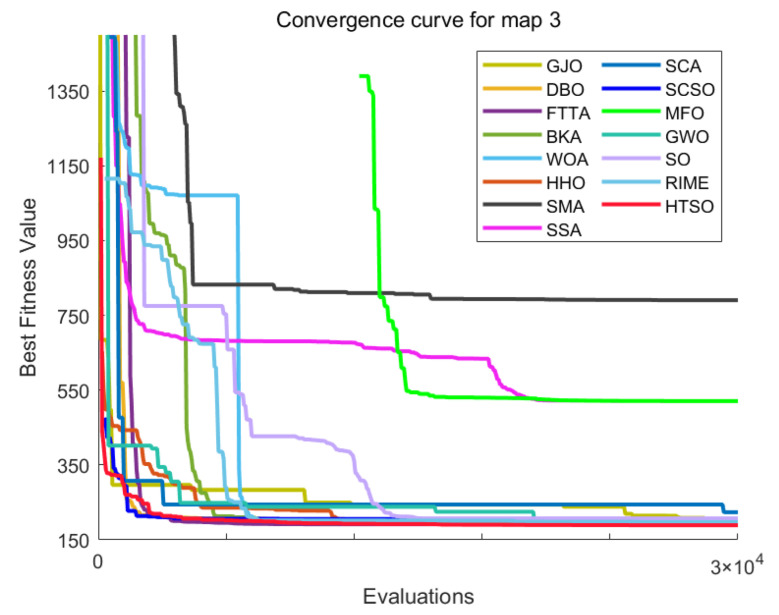
Convergence curve for Mountain No. 3.

The experimental results for Map 3 are presented in Table 20. HTSO achieved the best solution of 189.1797 and an average result of 250.6396; both values rank first among all algorithms. Furthermore, results from the Wilcoxon test demonstrate that HTSO achieves substantially superior performance compared to the other fourteen algorithms. Figure 46 displays the optimal 2D and 3D paths identified by the 15 algorithms. As shown in the convergence curves in Figure 47, HTSO is among the fastest algorithms to converge. Furthermore, there were cases in which GJO, WOA, HHO, SCSO, and GWO were unable to find a valid path.

#### 6.2.4. Model of Mountain No. 4

**Figure 48:**
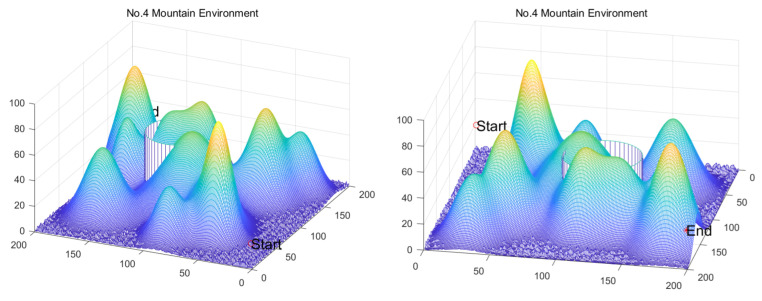
Mountain No. 4 environment simulation.

Figure 48 presents a schematic model of Mountain No. 4, which contains eleven peaks located at (60, 60, 50), (100, 100, 60), (180, 160, 80), (50, 140, 70), (50, 45, 65), (110, 150, 54), (170, 120, 50), (30, 170, 45), (160, 50, 55), (90, 30, 40), and (140, 140, 48).

**Figure 49:**
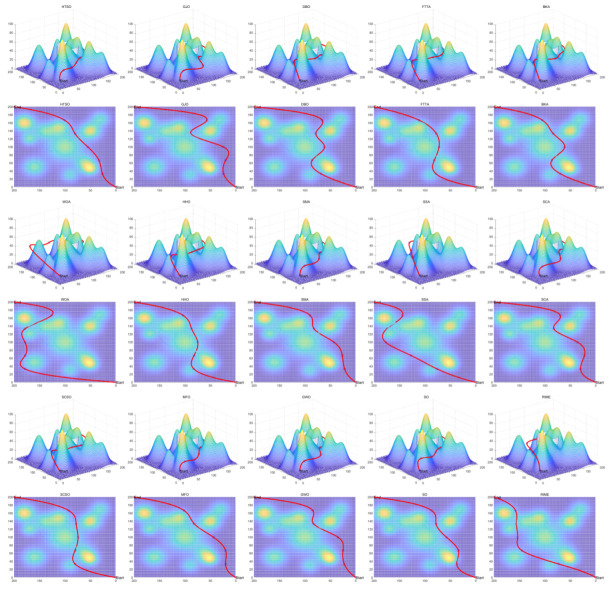
The generated UAV paths from fifteen algorithms of Mountain No. 4.

**Figure 50:**
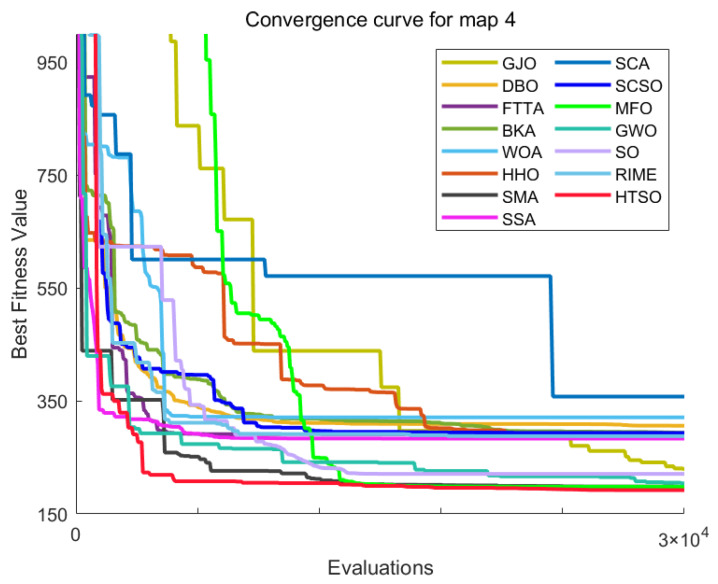
Convergence curve for Mountain No. 4.

The experimental results for this model are presented in Table 21. HTSO ranked first in both the best and average results, with a best value of 192.2244 and an average value of 267.3787. The results of the Wilcoxon test indicate that HTSO exhibits significant performance differences compared to GJO, DBO, WOA, HHO, SCA, SCSO, and GWO. Figure 49 displays the optimal paths obtained by the 15 algorithms. Figure 50 illustrates the convergence curves of the 15 algorithms, showing that HTSO achieves the fastest convergence and highest solution accuracy. In addition, HHO, SCSO, and GWO again failed to find valid paths.

#### 6.2.5. Model of Mountain No. 5

**Figure 51:**
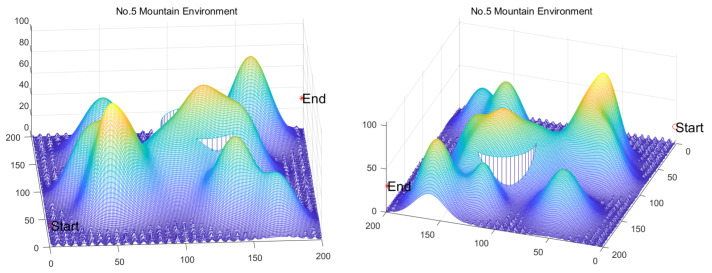
Mountain No. 5 environment simulation.

A schematic representation of Mountain No. 5 is shown in Figure 51. This mountain contains 13 peaks and is relatively complex. The coordinates of the peaks are as follows: (60, 60, 50), (100, 100, 60), (180, 160, 80), (50, 140, 70), (50, 45, 65), (110, 150, 54), (170, 120, 50), (30, 170, 45), (160, 50, 55), (90, 30, 40), (140, 140, 48), (120, 120, 45), and (80, 50, 52).

**Figure 52:**
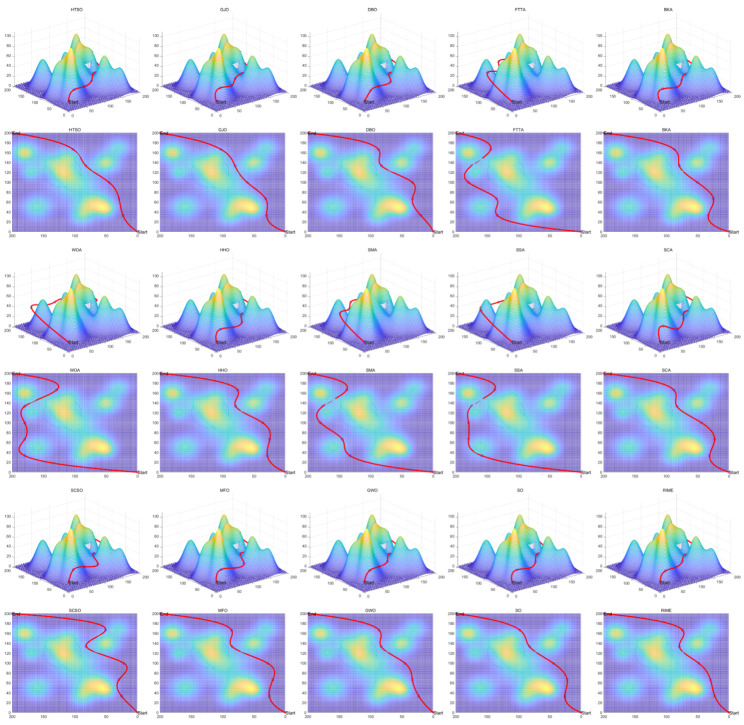
The generated UAV paths from fifteen algorithms of Mountain No. 5.

**Figure 53:**
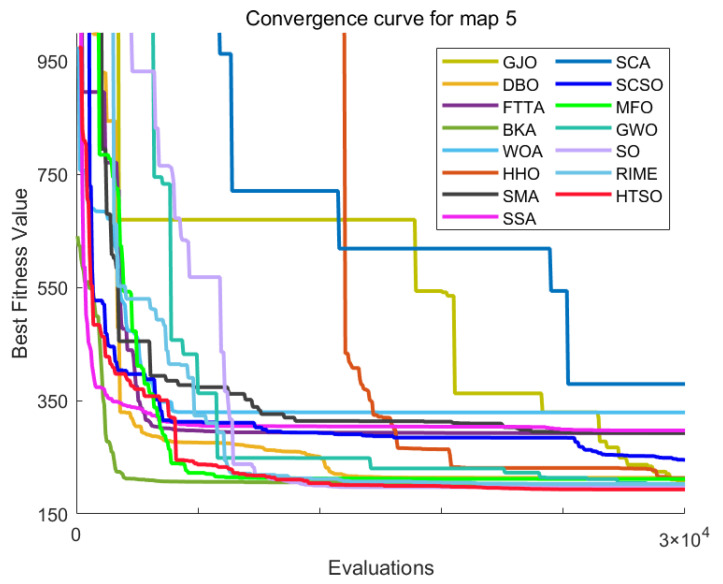
Convergence curve for Mountain No. 5.

As shown in Table 22, HTSO achieved the best result of 193.2201 and an average result of 295.4708, ranking first in both metrics. According to the Wilcoxon test, the performance difference between HTSO and BKA, SMA, SSA, and RIME is not significant, while significant differences exist between HTSO and the other ten algorithms. Figure 52 shows the 3D and 2D schematics of the best paths generated by the fifteen algorithms. Figure 53 displays the convergence curves corresponding to the optimal outcomes achieved by the fifteen different algorithms. Although BKA converges more rapidly to a near-optimal solution in the early iterations, it suffers from severe premature convergence, resulting in lower final accuracy compared to HTSO. In addition, HHO and GWO failed to find feasible paths for this problem.

#### 6.2.6. Model of Mountain No. 6

**Figure 54:**
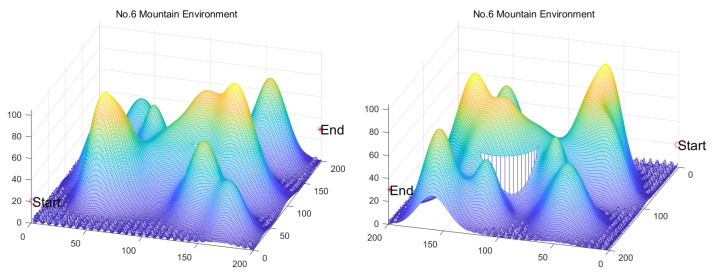
Mountain No. 6 environment simulation.

Figure 54 illustrates the schematic diagram of Mountain No. 6. This model is more complex, featuring 15 peaks located at the coordinates (60, 60, 50), (100, 100, 60), (180, 160, 80), (50, 140, 70), (50, 45, 65), (110, 150, 54), (170, 120, 50), (30, 170, 45), (160, 50, 55), (90, 30, 40), (140, 140, 48), (120, 120, 45), (80, 50, 52), (100, 160, 50), and (130, 70, 47).

**Figure 55:**
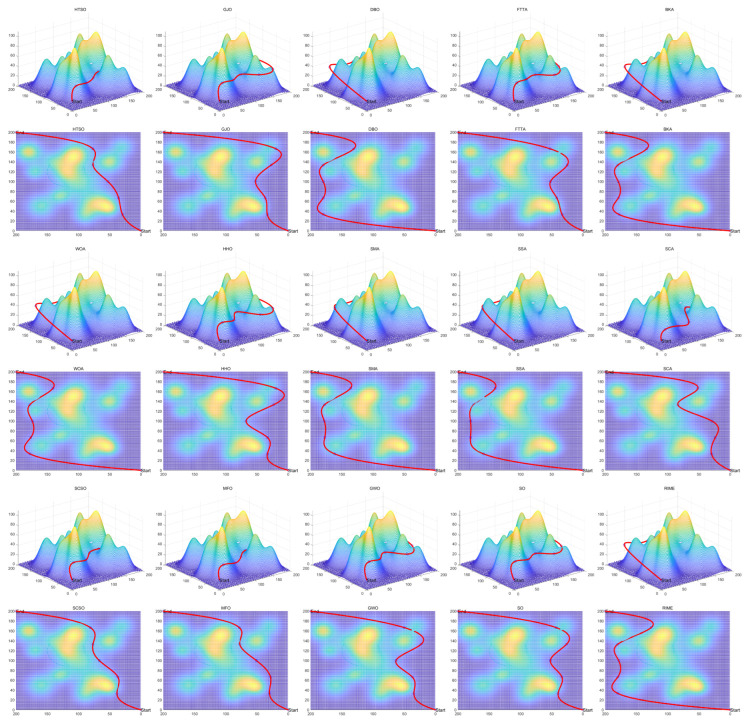
The generated UAV paths from fifteen algorithms of Mountain No. 6.

**Figure 56:**
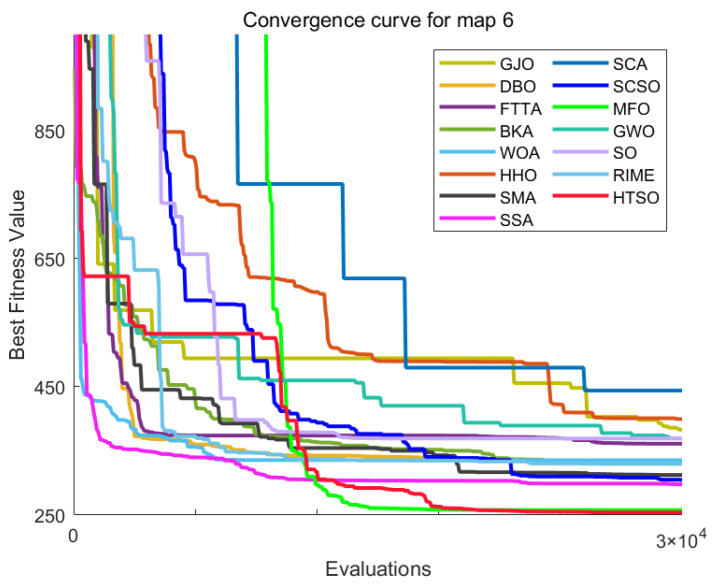
Convergence curve for Mountain No. 6.

As shown in Table 23, HTSO ranked first in both the best and average results. Figure 55 presents the optimal paths obtained by each of the 15 algorithms. Specifically, HTSO achieved a best result of 254.0815 and an average result of 330.6272. According to the Wilcoxon test, the performance differences between HTSO and DBO, BKA, SMA, and SSA are not significant, while HTSO demonstrates a significant performance advantage over the other 10 algorithms. Figure 56 displays the convergence curves for all 15 algorithms. It can be observed that although HTSO’s convergence rate in the early iterations is relatively unremarkable, its superior ability to escape local optima allows it to continually find new local optima, ultimately outperforming MFO in terms of convergence accuracy. Additionally, HHO, SCSO, and GWO exhibited abnormal convergence behavior.

#### 6.2.7. Model of Mountain No. 7

**Figure 57:**
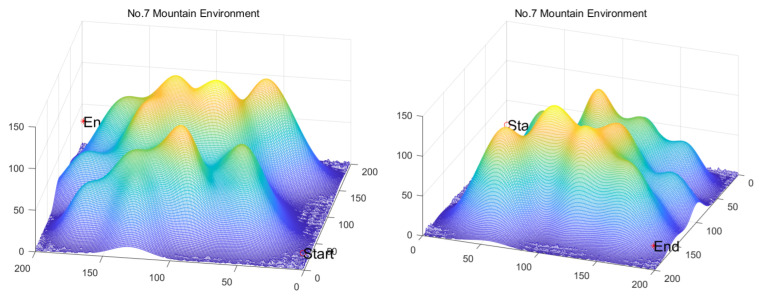
Mountain No. 7 environment simulation.

Mountain No. 7 is the most complex terrain among those studied, as illustrated in Figure 57. Its peaks are located at (50, 50, 60), (80, 120, 70), (120, 150, 85), (60, 80, 75), (100, 60, 80), (130, 120, 70), (140, 100, 65), (160, 150, 90), (170, 40, 50), (90, 40, 55), (110, 80, 60), (140, 60, 72), (60, 160, 80), (130, 30, 65), (90, 130, 70), (180, 100, 55), and (40, 160, 60). The terrain of this problem is extremely complex, making the optimization task highly challenging.

**Figure 58:**
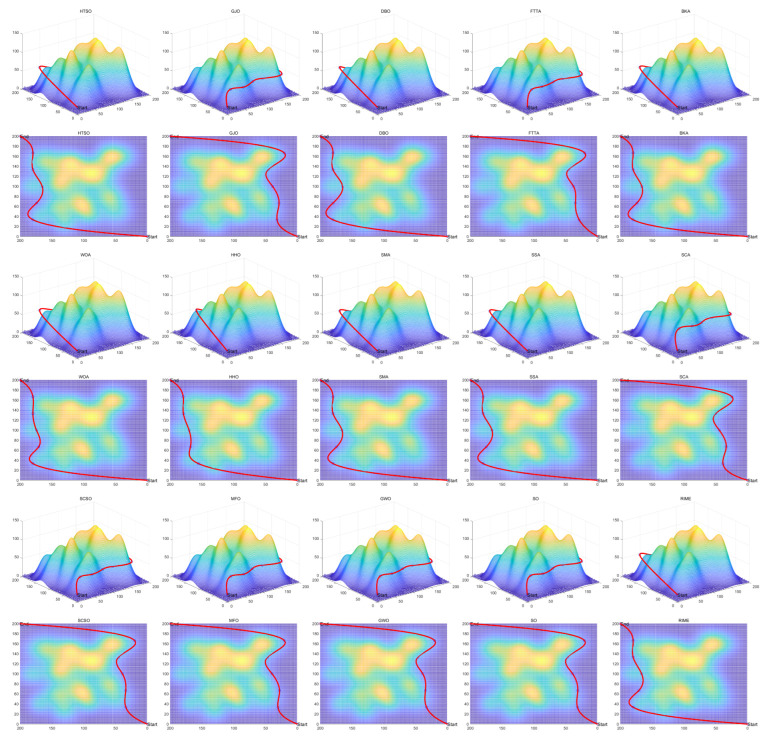
The generated UAV paths from fifteen algorithms of Mountain No. 7.

**Figure 59:**
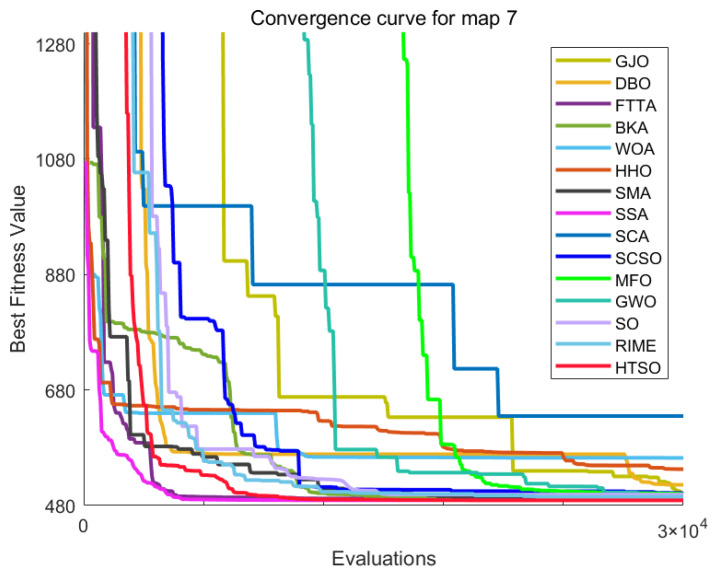
Convergence curve for Mountain No. 7.

The experimental results for Map 7 are presented in Table 24. HTSO demonstrated outstanding performance on this problem, achieving first place across all four evaluation metrics. The best obtained result was 488.9512, with an average result of 491.9125 and a standard deviation of only 4.5542, further highlighting HTSO’s stability and robustness in complex problems. According to the Wilcoxon test, HTSO demonstrates statistically significant differences in performance relative to every algorithm except for SMA and SSA. Figure 58 illustrates the best path trajectories generated by all algorithms. As shown in the convergence curve in Figure 59, HTSO’s superior population diversity and ability to escape local optima allowed it to consistently find new local optima during later iterations, ultimately surpassing competing methods in convergence accuracy. Additionally, WOA, HHO, and GWO still experienced convergence failures in this problem.

### 6.3. Experimental Summary of 3D Path Planning for UAV

**Figure 60:**
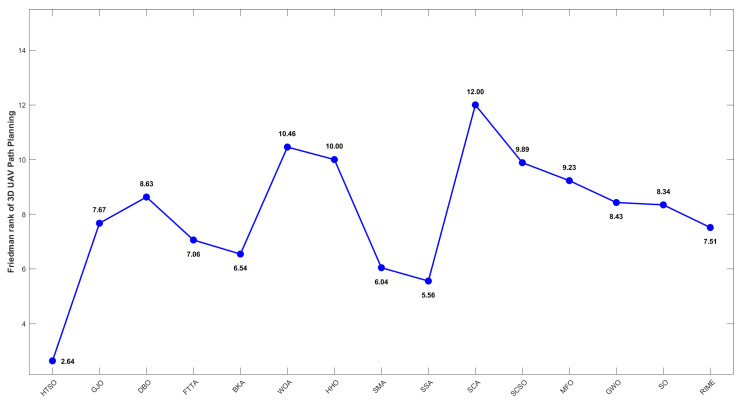
The average Friedman ranking for 3D path planning of UAVs.

In this section, we compare the performance of 15 different algorithms, including HTSO, on the 3D UAV path planning problem across seven distinct mountainous models. The evaluation criteria consist of the best solution, worst solution, mean result, standard deviation, and the Wilcoxon rank-sum test with a significance level of p=5%. Experimental results indicate that HTSO achieved first place in both the best and mean results across all seven mountainous models. Of the 98 Wilcoxon rank-sum tests performed across the seven problems, HTSO achieved statistically significant superiority over the comparison algorithms in 78 cases, accounting for 79.59% of all tests. In the remaining 20 cases (20.40%), there was no statistically significant difference between HTSO and the comparison algorithms. Importantly, there was no test in which HTSO was significantly outperformed by any comparison algorithm. Figure 60 presents a Friedman mean ranking plot of the 15 algorithms over the seven mountainous models; HTSO achieved an average rank of 2.64, securing first place overall and outperforming the second-place algorithm (SSA, with a mean rank of 5.56) by 2.92 ranks. Clearly, HTSO’s average rank far surpasses the other 14 algorithms. These results demonstrate that HTSO exhibits outstanding stability, robustness, superiority, reliability, and versatility in 3D mountainous UAV path planning problems, indicating stronger applicability compared to the other 14 algorithms.

## 7. Conclusions and Future Work

This paper introduces the Higher-order Thinking Skills Optimizer (HTSO), a novel meta-heuristic algorithm that bridges concepts from educational psychology with computational intelligence. It simulates principles such as metacognition, creativity, critical thinking, problem solving, and dynamic decision making. Each individual in HTSO is treated as an independent thinker who dynamically switches among three innovative search strategies—expert’s breakthrough, collaborative exploration, and paradigm shift—via a critical thinking (CT). This enables multi-level solution space exploration, ranging from expert to novice searches. During the problem-solving phase, thinkers focus on the region near the current optimal solution and employ the Lévy flight mechanism to emulate both inspirational leaps and gradual improvements. This effectively balances local exploitation with the ability to escape local optima. In the decision-making phase, the retention of superior individuals ensures overall solution quality. To validate the effectiveness of HTSO, it was compared with 21 algorithms (including CEC award-winning algorithms, widely applied algorithms or their improved versions, and recently proposed high-performance algorithms with high citation counts) across famous benchmark sets: CEC-2017 at dimensions of 30, 50, and 100, CEC-2020 at dimensions of 10, 15, and 20, and CEC-2022 at dimensions of 10 and 20. The results of the Wilcoxon rank-sum test indicate that, in 2961 comparisons across the dimensions of three CEC benchmark sets, HTSO significantly outperforms the comparison algorithms 2591 times, accounting for 87.50% of the total comparisons. Experimental results of HTSO are presented through various visualizations, which show that HTSO demonstrates superior convergence speed, accuracy, stability, effectiveness, robustness, and population vitality when compared to competing algorithms on the CEC test sets. In particular, the experimental data from the 100-dimensional CEC-2017 suite underscores HTSO’s unique ability to handle high-dimensional landscapes without the performance deterioration commonly seen in other metaheuristics.

To evaluate the capability of HTSO in solving real-world engineering problems, 15 algorithms, including HTSO, were tested on 12 constraint optimization problems related to mechanics. The results show that HTSO achieved the first place in the ranking based on the average results for each of the 12 problems. In terms of best performance, HTSO achieved first place in nine problems and second place in two problems.

Finally, to evaluate the effectiveness of HTSO in 3D unmanned aerial vehicle (UAV) path planning problems, a comparative analysis was conducted among 15 algorithms, including HTSO, on seven different mountainous terrain models. The experimental results demonstrate that HTSO achieved the best and average performance ranks in all seven terrain cases. These findings indicate that HTSO possesses strong applicability and generalizability for 3D mountainous UAV path planning problems.

All the aforementioned experiments demonstrate that HTSO is a highly effective novel meta-heuristic algorithm, suitable for a wide range of real-world constrained optimization problems as well as 3D UAV path planning problems, thereby providing a new and valuable approach to address optimization tasks. Furthermore, HTSO stands out as a user-friendly and low-parameter algorithm: the CT threshold is fixed at 0.5 throughout all experiments across different benchmark suites, engineering problems, and UAV path planning scenarios, without requiring any problem-specific adjustment. This parameter stability significantly reduces the burden of parameter tuning for practitioners and enhances the practical applicability of HTSO in real-world applications. However, the experimental results on the CEC benchmark set indicate that HTSO did not perform well on certain test functions. In addition, although the convergence accuracy of HTSO is outstanding, its convergence speed is relatively moderate on some problems. These observations suggest that there is still significant potential for further improvement of HTSO.

Future research could focus on several promising directions. First, enhancing the update mechanisms of the exploration and exploitation phases of HTSO deserves attention, for example, by integrating HTSO with other meta-heuristic algorithms or update strategies to achieve higher performance levels. Second, incorporating reinforcement learning techniques into HTSO represents a particularly compelling avenue. Recent advances have demonstrated the significant benefits of sparse update techniques in reinforcement learning for handling complex action space environments [149]. By leveraging such adaptive learning mechanisms, HTSO could potentially develop self-tuning capabilities that dynamically adjust search strategies based on the problem landscape, further reducing the need for manual parameter configuration while improving optimization performance. Third, HTSO could be extended to address large-scale real-world engineering challenges beyond the scope of current experiments. For instance, the coordinated space heating optimization of large building clusters presents a highly relevant application domain, where computational trade-offs in thermal energy aggregation and flexibility enhancement pose significant challenges [150]. Applying HTSO to such urban energy system optimization problems would not only validate its scalability but also contribute to sustainable energy management practices. Additionally, HTSO can be applied to multi-objective optimization problems or engineering problems with complex constraints, thereby expanding its application domains. The integration of HTSO with deep learning frameworks for feature extraction in high-dimensional optimization landscapes also presents an interesting research direction.

## Figures and Tables

**Table 1:** Overview of different categories of meta-heuristic algorithms.

Variety	Algorithm	Inspirational Origin	References	Year
Swarm	Ant Colony Algorithm (ACA)	Ant foraging and pheromones	[24]	1992
	Particle Swarm Optimization (PSO)	Foraging strategies of bird flocks	[25]	1995
	Cuckoo Search (CS)	Brood parasitism of cuckoo birds	[26]	2009
	Bat-inspired Algorithm (BA)	Prey-capture and obstacle-avoidance behaviors of bats	[27]	2010
	Gray Wolf Optimizer (GWO)	Hunting & hierarchy of wolves	[28]	2014
	Moth–Flame Optimization (MFO)	Transverse orientation strategy of moths	[29]	2015
	Whale Optimization Algorithm (WOA)	Bubble-net hunting of humpbacks	[30]	2016
	Harris Hawk Optimization (HHO)	Surprise attacks of Harris’ hawks	[31]	2019
	Sparrow Search Algorithm (SSA)	Foraging behavior of sparrows	[32]	2020
	Marine Predator Algorithm (MPA)	Foraging strategies of marine predators	[33]	2020
	Slime Mould Algorithm (SMA)	Foraging behavior of slime molds	[34]	2020
	African Vulture Optimization Algorithm (AVOA)	Foraging and navigational behaviors of African vultures	[35]	2021
	Artificial Gorilla Troops Optimizer (GTO)	The social behaviors of gorilla groups	[36]	2021
	Aquila Optimizer (AO)	Prey-capture strategies of aquila	[37]	2021
	Golden Jackal Optimization (GJO)	Golden jackals’ hunting teamwork	[38]	2022
	Snake Optimizer (SO)	Snake predation and reproductive behavior	[39]	2022
	Artificial Rabbits Optimization (ARO)	Survival strategies of the rabbit	[40]	2022
	Honey Badger Algorithm (HBA)	Digging and honey-foraging behavior of the honey badger	[41]	2022
	Zebra Optimization Algorithm (ZOA)	Foraging behavior and anti-predator strategies of zebras	[42]	2022
	Dung Beetle Optimizer (DBO)	Dung beetles	[43]	2023
	Nutcracker Optimizer (NOA)	Foraging, caching, search and recovery behaviors of the nutcracker	[44]	2023
	Sand Cat Swarm Optimization (SCSO)	Foraging, predatory, detection behaviors of sand cat	[45]	2023
	Black-winged Kite Algorithm (BKA)	Predatory strategies and migratory patterns of the black-winged kite	[46]	2024
	Secretary Bird Optimization algorithm (SBOA)	Survival strategies of the secretary bird	[47]	2024
	Crested Porcupine Optimizer (CPO)	Foraging and survival strategies of crested porcupine	[48]	2024
	Hippopotamus Optimization Algorithm (HO)	Movement, defense and evasion strategies of the hippopotamus	[49]	2024
Evolution	Evolutionary Programming (EP)	Evolutionary changes in group behavior	[50]	1965
	Genetic Algorithm (GA)	Charles Darwin’s theory of evolution	[51]	1975
	Genetic Programming (GP)	Biological evolution	[52]	1992
	Differential Evolution (DE)	Natural evolution and biological principles of mutation	[53]	1995
	Biogeography-Based Optimization (BBO)	Species distribution and migration patterns	[54]	2008
	Forest Optimization Algorithm (FOA)	Growth, competition, and cooperation among forest plants	[55]	2014
	Tree–Seed Algorithm (TSA)	Seed-based reproductive processes in trees	[56]	2015
	Artificial Infectious Disease (AID)	SEIQR epidemic model	[57]	2016
	Black Widow Optimization (BWO)	Mating behavior of the black widow spider	[58]	2020
	Fungi Kingdom Expansion (FKE)	Expansion behavior of fungi	[59]	2021
	Human Felicity Algorithm (HFA)	Human behavior in the pursuit of happiness	[60]	2022
	Fungal Growth Optimizer (FGO)	Fungal growth and modes of reproduction	[61]	2025
Physics	Simulated Annealing (SA)	Metal cooling	[62]	1983
	Big Bang–Big Crunch Algorithm (BBBC)	Cosmological theories of the Big Bang and the Big Crunch	[63]	2006
	Central Force Optimization (CFO)	Gravitational kinematics	[64]	2007
	Intelligent Water Drops Algorithm (IWDA)	The dynamics and morphological evolution of water droplets	[65]	2009
	Water Cycle Algorithm (WCA)	The natural water cycle	[66]	2012
	Multi-Verse Optimization Algorithm (MVO)	Multiverse theory	[67]	2016
	Turbulent Flow of Water Optimization (TFWO)	The natural motion of oceanic vortices	[68]	2020
	Equilibrium Optimizer (EO)	Control volume models	[69]	2020
	Elastic Deformation Optimization Algorithm (EDOA)	Hooke’s law of elasticity and Newton’s second law of motion	[70]	2022
	Homonuclear Molecules Optimization (HMO)	Electronic configuration of an atom	[71]	2022
	Kepler Optimization Algorithm (KOA)	Kepler’s Laws of Planetary Motion	[72]	2023
	Fick’s Law Algorithm (FLA)	Fick’s law of diffusion	[73]	2023
	Rime Optimizer (RIME)	Rime formation and growth in natural environments	[74]	2023
	Great Wall Construction Algorithm (GWCA)	Labor competition in the Great Wall construction	[75]	2023
	Geyser-inspired Algorithm (GEA)	Geysers as a geological phenomenon in nature	[76]	2024
	Newton–Raphson-Based Optimizer (NRBO)	Newton–Raphson method	[77]	2024
Mathematics	Sine Cosine Algorithm (SCA)	Periodic properties of sine and cosine functions	[78]	2016
	Golden Sine algorithm (Gold-SA)	Sine function & Golden Ratio	[79]	2017
	Gradient-Based Optimizer (GBO)	Gradient-based Newton’s method	[80]	2020
	Arithmetic Optimization Algorithm (AOA)	Basic operations of arithmetic	[81]	2021
	Circle Search Algorithm (CSA)	The geometrical features of circles	[82]	2022
	Quadratic Interpolation Optimization (QIO)	Generalized quadratic interpolation	[83]	2023
	Subtraction–Average-Based Optimizer (SABO)	The subtraction average of searcher agents	[84]	2023
	Sinh Cosh Optimizer (SCHO)	The sine and cosine functions	[85]	2023
Human	Tabu Search (TS)	Cognitive strategies for preventing redundant attempts in human problem solving	[86]	1986
	Teaching–Learning Based Optimization (TLBO)	Teacher instruction and student learning processes	[87]	2011
	Social Learning Optimization (SLO)	Observational learning in humans	[88]	2011
	Brain Storm Optimization (BSO)	Multiple individuals generating novel perspectives on one subject	[89]	2011
	Exchange Market Algorithm (EMA)	The procedure of trading the shares on stock market	[90]	2014
	Soccer League Competition (SLC)	Club- and player-level competition within association football leagues	[91]	2014
	Social Evolution and Learning Optimization (SELO)	Family-based social learning behavior	[92]	2018
	Volleyball Premier League (VPL)	The competition and interaction among volleyball teams	[93]	2018
	Political Optimizer (PO)	Multiphase political process	[94]	2020
	Hunger Games Search (HGS)	Animal foraging behavior	[95]	2021
	Puzzle Optimization Algorithm (POA)	The process of solving a puzzle	[96]	2022
	Hunter Prey Optimization (HPO)	Predation processes among wild animals	[97]	2022
	Driving Training-Based Optimization (DTBO)	The behavior of learning to drive an automobile	[98]	2022
	War Strategy Optimization (WSO)	Reconnaissance, offense, defense, and alliance behaviors in warfare	[99]	2022
	Mountaineering Team-Based Optimization (MTBO)	Coordinated actions of mountaineers in response to environmental challenges	[100]	2023
	City Councils Evolution (CCE)	The city council’s evolution	[101]	2023
	Kids Learning Optimizer (KLO)	Modeled on children’s early family-based social learning	[102]	2024
	Football Team Training Algorithm (FTTA)	Training models in football teams	[103]	2024
	Information Acquisition Optimizer (IAO)	Human information acquisition behavior	[104]	2024
	Hiking Optimization Algorithm (HOA)	The experience of hikers attempting to summit peaks	[105]	2024
	Dream Optimization Algorithm (DOA)	Human dreams	[106]	2025

**Table 2:** The parameter settings for compared algorithms.

Algorithms	Parameter	Value	Algorithms	Parameter	Value
DE	F;CR	0.8;0.1	BKA	P;r	0.9;[0,1]
LSHADE	Pb;Arc_rate	0.1;2	FTTA	tp	tdistribution
LSHADE_SPACMA	Pb	0.1	SCSO	SM	2
	Arc_rate	2		RouletteWheel	[0,360]
	L_rate	0.8	AO	α; δ	0.1;0.1
LSHADE-cnEpSin	pb;ps	0.4;0.5	SO	$c_{1}$; $c_{2}$; $c_{3}$	0.5;0.05;0.2
	freq_init	0.5		Threshold	0.25
MELGWO	*a*	[0,2]		Threshold2	0.6
	Crossover	0.6	AVOA	$L_{1}$	[0.7,0.9]
	SL_Search	0.5		$L_{2}$	[0.1,0.3]
PPSO	*p*	0.02		*w*	[2,3]
	structure	{2,4,10,14}		$P_{1}$	[0.4,0.6]
WOA	*a*	Linearly2to0		$P_{2}$	[0.4,0.6]
	*b*	1		$P_{3}$	[0.4,0.6]
SSA	$c_{1}$	Linearly2to0	GTO	p;β;w	0.03;3;8
HO	-	-	RIME	*W*	5
GJO	$c_{1}$	1.5	SCA	*a*	2
DBO	k;b;s	0.1;0.3;0.5	GWO	*a*	Linearly2to0
SBOA	CF	[0,1]	GBO	$pr;\beta_{min};\beta_{max}$	0.5;0.2;1.2
	*K*	1,2	HHO	$E_{0}$	(−1,1)
	$R_{1}$	[0,1]	SMA	*z*	0.03
	$R_{2}$	[0,1]	MFO	*a*	Linearly−1to−2
HTSO	CT	0.5			

**Table 3:** Description of the CEC-2017 test set.

Type	No.	CEC-2017 Function Name	Range	Dimension	$f_{\min}$
Unimodal	F1	Shifted and rotated bent cigar function	[−100, 100]	30/50/100	100
	F3	Shifted and rotated Zakharov function	[−100, 100]	30/50/100	300
Multimodal	F4	Shifted and rotated Rosenbrock’s function	[−100, 100]	30/50/100	400
	F5	Shifted and rotated Rastrigin’s Function	[−100, 100]	30/50/100	500
	F6	Shifted and rotated expanded Scaffer’s F6 Function	[−100, 100]	30/50/100	600
	F7	Shifted and ROTATED Lunacek Bi-Rastrigin function	[−100, 100]	30/50/100	700
	F8	Shifted and rotated non-continuous Rastrigin’s function	[−100, 100]	30/50/100	800
	F9	Shifted and rotated lévy function	[−100, 100]	30/50/100	900
	F10	Shifted and rotated Schwefel’s function	[−100, 100]	30/50/100	1000
Hybrid	F11	Hybrid function 1 (N = 3)	[−100, 100]	30/50/100	1100
	F12	Hybrid function 2 (N = 3)	[−100, 100]	30/50/100	1200
	F13	Hybrid function 3 (N = 3)	[−100, 100]	30/50/100	1300
	F14	Hybrid function 4 (N = 4)	[−100, 100]	30/50/100	1400
	F15	Hybrid function 5 (N = 4)	[−100, 100]	30/50/100	1500
	F16	Hybrid function 6 (N = 4)	[−100, 100]	30/50/100	1600
	F17	Hybrid function 6 (N = 5)	[−100, 100]	30/50/100	1700
	F18	Hybrid function 6 (N = 5)	[−100, 100]	30/50/100	1800
	F19	Hybrid function 6 (N = 5)	[−100, 100]	30/50/100	1900
	F20	Hybrid function 6 (N = 6)	[−100, 100]	30/50/100	2000
Composition	F21	Composition function 1 (N = 3)	[−100, 100]	30/50/100	2100
	F22	Composition function 2 (N = 3)	[−100, 100]	30/50/100	2200
	F23	Composition function 3 (N = 4)	[−100, 100]	30/50/100	2300
	F24	Composition function 4 (N = 4)	[−100, 100]	30/50/100	2400
	F25	Composition function 5 (N = 5)	[−100, 100]	30/50/100	2500
	F26	Composition function 6 (N = 5)	[−100, 100]	30/50/100	2600
	F27	Composition function 7 (N = 6)	[−100, 100]	30/50/100	2700
	F28	Composition function 8 (N = 6)	[−100, 100]	30/50/100	2800
	F29	Composition function 9 (N = 3)	[−100, 100]	30/50/100	2900
	F30	Composition function 10 (N = 3)	[−100, 100]	30/50/100	3000

**Table 4:** Description of the CEC-2020 test set.

Type	No.	CEC-2020 Function Name	Range	Dimension	$f_{\min}$
Unimodal	F1	Shifted and Rotated Bent Cigar Function (CEC-2017 F1)	[−100, 100]	10/15/20	100
Multimodal	F2	Shifted and Rotated Schwefel’s Function (CEC-2014 [134] F11)	[−100, 100]	10/15/20	1100
	F3	Rotated Lunacek Bi-Rastrigin Function (CEC-2017 F7)	[−100, 100]	10/15/20	700
	F4	Expanded Rosenbrock’s plus Griewangk’s Function (CEC-2017 F19)	[−100, 100]	10/15/20	1900
Hybrid	F5	Hybrid Function 1 (N = 3) (CEC-2014 F17)	[−100, 100]	10/15/20	1700
	F6	Hybrid Function 2 (N = 4) (CEC-2017 F16)	[−100, 100]	10/15/20	1600
	F7	Hybrid Function 3 (N = 5) (CEC-2014 F21)	[−100, 100]	10/15/20	2100
Composition	F8	Composition Function 1 (N = 3) (CEC-2017 F22)	[−100, 100]	10/15/20	2200
	F9	Composition Function 2 (N = 4) (CEC-2017 F24)	[−100, 100]	10/15/20	2400
	F10	Composition Function 3 (N = 5) (CEC-2017 F25)	[−100, 100]	10/15/20	2500

**Table 5:** Description of the CEC-2022 test set.

Type	No.	CEC-2022 Function Name	Range	Dimension	$f_{\min}$
Unimodal	F1	Shifted and full rotated Zakharov function	[−100, 100]	10/20	300
Multimodal	F2	Shifted and full rotated Rosenbrock’s function	[−100, 100]	10/20	400
	F3	Shifted and full rotated Rastrigin’s function	[−100, 100]	10/20	600
	F4	Shifted and full rotated non-continuous Rastrigin’s function	[−100, 100]	10/20	800
	F5	Shifted and full rotated lévy function	[−100, 100]	10/20	900
Hybrid	F6	Hybrid function 1 (N = 3)	[−100, 100]	10/20	1800
	F7	Hybrid function 2 (N = 6)	[−100, 100]	10/20	2000
	F8	Hybrid function 3 (N = 5)	[−100, 100]	10/20	2200
Composition	F9	Composition function 1 (N = 5)	[−100, 100]	10/20	2300
	F10	Composition function 2 (N = 4)	[−100, 100]	10/20	2400
	F11	Composition function 3 (N = 5)	[−100, 100]	10/20	2600
	F12	Composition function 4 (N = 6)	[−100, 100]	10/20	2700

**Table 6:** The optimal statistical results for TBTD.

Algorithm	Worst	Best	Std	Mean	Best Rank	Mean Rank	Wilcoxon
HTSO	263.8958	263.8958	0.0000	263.8958	1	1	
GWO	263.9259	263.8976	0.0081	263.9054	10	8	0.0002 (+)
BKA	263.9008	263.8958	0.0016	263.8968	4	4	0.0002 (+)
AVOA	264.0184	263.8980	0.0385	263.9334	11	10	0.0002 (+)
HO	263.9031	263.8959	0.0022	263.8971	5	7	0.0002 (+)
GTO	263.8959	263.8958	0.0000	263.8959	2	3	0.0002 (+)
RIME	267.0086	263.8992	1.1064	264.7942	12	11	0.0002 (+)
SCA	282.8427	263.9770	5.9200	265.9971	15	14	0.0002 (+)
FTTA	263.9022	263.8959	0.0019	263.8969	7	5	0.0002 (+)
WOA	282.8427	263.9206	5.8404	266.8529	14	15	0.0002 (+)
SO	263.9030	263.8959	0.0022	263.8971	6	6	0.0002 (+)
GJO	282.8427	263.9027	5.9787	265.8274	13	13	0.0002 (+)
DBO	263.9401	263.8966	0.0129	263.9081	8	9	0.0002 (+)
SCSO	282.8427	263.8967	5.9903	265.7940	9	12	0.0002 (+)
GBO	263.8959	263.8958	0.0000	263.8958	3	2	0.0002 (+)

**Table 7:** The optimal statistical results for TCPD (Case 1).

Algorithm	Worst	Best	Std	Mean	Best Rank	Mean Rank	Wilcoxon
HTSO	$1.267\times 10^{-2}$	$1.267\times 10^{-2}$	$3.942\times 10^{-7}$	$1.267\times 10^{-2}$	1	1	
GWO	$1.297\times 10^{-2}$	$1.270\times 10^{-2}$	$7.657\times 10^{-5}$	$1.276\times 10^{-2}$	8	3	0.0002 (+)
BKA	$1.325\times 10^{-2}$	$1.268\times 10^{-2}$	$1.743\times 10^{-4}$	$1.276\times 10^{-2}$	2	4	0.0002 (+)
AVOA	$1.507\times 10^{-2}$	$1.270\times 10^{-2}$	$7.097\times 10^{-4}$	$1.326\times 10^{-2}$	9	9	0.0002 (+)
HO	$1.470\times 10^{-2}$	$1.270\times 10^{-2}$	$6.827\times 10^{-4}$	$1.323\times 10^{-2}$	7	8	0.0002 (+)
GTO	$1.272\times 10^{-2}$	$1.268\times 10^{-2}$	$1.535\times 10^{-5}$	$1.270\times 10^{-2}$	3	2	0.0002 (+)
RIME	$1.810\times 10^{-2}$	$1.271\times 10^{-2}$	$2.091\times 10^{-3}$	$1.628\times 10^{-2}$	10	15	0.0002 (+)
SCA	$1.524\times 10^{-2}$	$1.290\times 10^{-2}$	$6.785\times 10^{-4}$	$1.335\times 10^{-2}$	15	11	0.0002 (+)
FTTA	$1.335\times 10^{-2}$	$1.270\times 10^{-2}$	$2.372\times 10^{-4}$	$1.289\times 10^{-2}$	6	6	0.0002 (+)
WOA	$1.555\times 10^{-2}$	$1.271\times 10^{-2}$	$8.159\times 10^{-4}$	$1.355\times 10^{-2}$	11	12	0.0002 (+)
SO	$1.533\times 10^{-2}$	$1.268\times 10^{-2}$	$9.028\times 10^{-4}$	$1.330\times 10^{-2}$	4	10	0.0002 (+)
GJO	$1.347\times 10^{-2}$	$1.272\times 10^{-2}$	$2.735\times 10^{-4}$	$1.297\times 10^{-2}$	12	7	0.0002 (+)
DBO	$1.814\times 10^{-2}$	$1.278\times 10^{-2}$	$2.141\times 10^{-3}$	$1.464\times 10^{-2}$	14	14	0.0002 (+)
SCSO	$1.599\times 10^{-2}$	$1.272\times 10^{-2}$	$8.630\times 10^{-4}$	$1.382\times 10^{-2}$	13	13	0.0002 (+)
GBO	$1.316\times 10^{-2}$	$1.269\times 10^{-2}$	$1.478\times 10^{-4}$	$1.279\times 10^{-2}$	5	5	0.0002 (+)

**Table 8:** The optimal statistical results for PVD.

Algorithm	Worst	Best	Std	Mean	Best Rank	Mean Rank	Wilcoxon
HTSO	6049.8580	6049.8580	0.0000	6049.8580	2	1	
GWO	7390.8896	6049.9269	421.8297	6190.9717	5	3	0.0002 (+)
BKA	7330.6289	6081.8604	347.3981	6538.3208	10	8	0.0002 (+)
AVOA	7378.6147	6051.5064	491.2082	6681.4138	8	11	0.0002 (+)
HO	8117.2102	6404.3931	566.6917	7049.1166	15	13	0.0002 (+)
GTO	7330.6282	6049.8580	508.3019	6578.9587	1	9	0.0253 (+)
RIME	7388.8849	6082.1600	441.7484	6702.3466	12	12	0.0002 (+)
SCA	8799.8575	6228.8329	782.2082	7112.6452	13	14	0.0002 (+)
FTTA	7385.0652	6081.8714	467.4194	6595.2928	11	10	0.0002 (+)
WOA	10,294.8386	6343.5948	1607.3886	7851.6055	14	15	0.0002 (+)
SO	6581.3982	6049.8635	181.6834	6165.3420	4	2	0.0002 (+)
GJO	7347.6444	6051.0773	404.8686	6260.8323	7	4	0.0002 (+)
DBO	7385.0652	6077.9134	450.1727	6441.9363	9	7	0.0002 (+)
SCSO	7372.1631	6050.1210	391.1721	6340.9311	6	5	0.0002 (+)
GBO	6816.7365	6049.8580	299.5867	6415.2300	3	6	0.0002 (+)

**Table 9:** The optimal statistical results for GTD.

Algorithm	Worst	Best	Std	Mean	Best Rank	Mean Rank	Wilcoxon
HTSO	$2.308\times 10^{-11}$	$2.701\times 10^{-12}$	$1.052\times 10^{-11}$	$1.085\times 10^{-11}$	1	1	
GWO	$1.362\times 10^{-9}$	$2.701\times 10^{-12}$	$5.564\times 10^{-10}$	$4.421\times 10^{-10}$	2	4	0.0083 (+)
BKA	$9.746\times 10^{-10}$	$2.701\times 10^{-12}$	$3.963\times 10^{-10}$	$2.263\times 10^{-10}$	3	2	0.0261 (+)
AVOA	$9.521\times 10^{-9}$	$8.888\times 10^{-10}$	$2.562\times 10^{-9}$	$2.760\times 10^{-9}$	12	11	0.0001 (+)
HO	$2.358\times 10^{-9}$	$2.308\times 10^{-11}$	$9.440\times 10^{-10}$	$1.124\times 10^{-9}$	6	8	0.0004 (+)
GTO	$8.701\times 10^{-9}$	$2.308\times 10^{-11}$	$2.625\times 10^{-9}$	$1.858\times 10^{-9}$	7	10	0.0004 (+)
RIME	$2.358\times 10^{-9}$	$2.308\times 10^{-11}$	$1.023\times 10^{-9}$	$1.255\times 10^{-9}$	8	9	0.0004 (+)
SCA	$1.827\times 10^{-8}$	$1.263\times 10^{-9}$	$5.201\times 10^{-9}$	$4.024\times 10^{-9}$	15	12	0.0001 (+)
FTTA	$2.407\times 10^{-8}$	$2.308\times 10^{-11}$	$7.449\times 10^{-9}$	$4.964\times 10^{-9}$	9	13	0.0002 (+)
WOA	$2.614\times 10^{-8}$	$9.922\times 10^{-10}$	$9.339\times 10^{-9}$	$7.977\times 10^{-9}$	13	15	0.0001 (+)
SO	$9.922\times 10^{-10}$	$2.701\times 10^{-12}$	$3.852\times 10^{-10}$	$2.865\times 10^{-10}$	4	3	0.0341 (+)
GJO	$2.358\times 10^{-9}$	$2.308\times 10^{-11}$	$9.177\times 10^{-10}$	$9.505\times 10^{-10}$	10	5	0.0007 (+)
DBO	$2.727\times 10^{-8}$	$9.922\times 10^{-10}$	$8.939\times 10^{-9}$	$6.853\times 10^{-9}$	14	14	0.0001 (+)
SCSO	$2.358\times 10^{-9}$	$2.308\times 10^{-11}$	$1.011\times 10^{-9}$	$1.114\times 10^{-9}$	11	7	0.0004 (+)
GBO	$1.362\times 10^{-9}$	$2.701\times 10^{-12}$	$5.233\times 10^{-10}$	$9.596\times 10^{-10}$	5	6	0.0016 (+)

**Table 10:** The optimal statistical results for HSTB.

Algorithm	Worst	Best	Std	Mean	Best Rank	Mean Rank	Wilcoxon
HTSO	1811.2674	1621.4567	74.0114	1698.4287	4	1	
GWO	2361.8151	1841.2334	171.7079	2049.5066	8	4	0.0002 (+)
BKA	2596.7070	1686.5263	312.2410	2061.4991	6	5	0.0010 (+)
AVOA	4692.9580	1279.4001	916.3346	2553.6956	1	10	0.0028 (+)
HO	3371.6043	2241.4522	400.8256	2791.0719	14	11	0.0002 (+)
GTO	2300.3973	1618.8234	220.8118	1817.5060	3	2	0.1405 (=)
RIME	3577.9556	1529.5782	608.5448	2192.2035	2	6	0.0091 (+)
SCA	6648.3360	2855.4202	1200.0877	4242.6925	15	15	0.0002 (+)
FTTA	5393.4931	1874.2496	1160.2564	2912.9844	9	12	0.0002 (+)
WOA	6551.1213	2067.1989	1749.4340	3046.3244	13	14	0.0002 (+)
SO	2866.8789	1944.6880	294.3821	2465.6009	10	8	0.0002 (+)
GJO	2701.0796	1952.5138	253.3734	2314.6624	11	7	0.0002 (+)
DBO	5942.9622	1697.0351	1244.9994	2991.5032	7	13	0.0004 (+)
SCSO	3269.5897	2010.6339	413.1019	2533.3918	12	9	0.0002 (+)
GBO	2607.3399	1630.2429	347.3057	2022.7236	5	3	0.0211 (+)

**Table 11:** The optimal statistical results for MDCB.

Algorithm	Worst	Best	Std	Mean	Best Rank	Mean Rank	Wilcoxon
HTSO	$2.352\times 10^{-1}$	$2.352\times 10^{-1}$	$2.926\times 10^{-17}$	$2.352\times 10^{-1}$	1	1	
GWO	$2.353\times 10^{-1}$	$2.353\times 10^{-1}$	$2.660\times 10^{-5}$	$2.353\times 10^{-1}$	11	11	0.0001 (+)
BKA	$2.352\times 10^{-1}$	$2.352\times 10^{-1}$	$3.336\times 10^{-17}$	$2.352\times 10^{-1}$	1	1	NaN (=)
AVOA	$2.353\times 10^{-1}$	$2.352\times 10^{-1}$	$2.248\times 10^{-5}$	$2.353\times 10^{-1}$	9	10	0.0001 (+)
HO	$2.354\times 10^{-1}$	$2.353\times 10^{-1}$	$4.837\times 10^{-5}$	$2.353\times 10^{-1}$	12	12	0.0001 (+)
GTO	$2.352\times 10^{-1}$	$2.352\times 10^{-1}$	$2.926\times 10^{-17}$	$2.352\times 10^{-1}$	1	1	NaN (=)
RIME	$2.358\times 10^{-1}$	$2.353\times 10^{-1}$	$1.893\times 10^{-4}$	$2.355\times 10^{-1}$	13	14	0.0001 (+)
SCA	$2.407\times 10^{-1}$	$2.355\times 10^{-1}$	$1.601\times 10^{-3}$	$2.377\times 10^{-1}$	15	15	0.0001 (+)
FTTA	$2.352\times 10^{-1}$	$2.352\times 10^{-1}$	$3.336\times 10^{-17}$	$2.352\times 10^{-1}$	1	1	NaN (=)
WOA	$2.352\times 10^{-1}$	$2.352\times 10^{-1}$	$9.108\times 10^{-8}$	$2.352\times 10^{-1}$	8	8	0.0001 (+)
SO	$2.352\times 10^{-1}$	$2.352\times 10^{-1}$	$4.340\times 10^{-17}$	$2.352\times 10^{-1}$	1	1	NaN (=)
GJO	$2.357\times 10^{-1}$	$2.353\times 10^{-1}$	$1.428\times 10^{-4}$	$2.354\times 10^{-1}$	14	13	0.0001 (+)
DBO	$2.352\times 10^{-1}$	$2.352\times 10^{-1}$	$3.701\times 10^{-17}$	$2.352\times 10^{-1}$	1	1	NaN (=)
SCSO	$2.353\times 10^{-1}$	$2.352\times 10^{-1}$	$2.014\times 10^{-6}$	$2.353\times 10^{-1}$	10	9	0.0001 (+)
GBO	$2.352\times 10^{-1}$	$2.352\times 10^{-1}$	$2.926\times 10^{-17}$	$2.352\times 10^{-1}$	1	1	NaN (=)

**Table 12:** The optimal statistical results for PGTD.

Algorithm	Worst	Best	Std	Mean	Best Rank	Mean Rank	Wilcoxon
HSTO	$5.371\times 10^{-1}$	$5.263\times 10^{-1}$	$4.198\times 10^{-3}$	$5.299\times 10^{-1}$	2	1	
GWO	$5.482\times 10^{-1}$	$5.300\times 10^{-1}$	$5.486\times 10^{-3}$	$5.370\times 10^{-1}$	8	7	0.0061 (+)
BKA	$5.497\times 10^{-1}$	$5.274\times 10^{-1}$	$6.416\times 10^{-3}$	$5.352\times 10^{-1}$	5	5	0.0478 (+)
AVOA	$5.742\times 10^{-1}$	$5.274\times 10^{-1}$	$1.353\times 10^{-2}$	$5.381\times 10^{-1}$	6	8	0.0316 (+)
HO	$6.031\times 10^{-1}$	$5.274\times 10^{-1}$	$2.143\times 10^{-2}$	$5.427\times 10^{-1}$	7	11	0.0036 (+)
GTO	$5.479\times 10^{-1}$	$5.300\times 10^{-1}$	$5.222\times 10^{-3}$	$5.361\times 10^{-1}$	9	6	0.0137 (+)
RIME	$5.401\times 10^{-1}$	$5.260\times 10^{-1}$	$5.782\times 10^{-3}$	$5.331\times 10^{-1}$	1	2	0.2550 (=)
SCA	$1.481\times 10^{0}$	$6.613\times 10^{-1}$	$2.775\times 10^{-1}$	$8.553\times 10^{-1}$	15	15	0.0002 (+)
FTTA	$1.373\times 10^{0}$	$5.331\times 10^{-1}$	$2.731\times 10^{-1}$	$6.608\times 10^{-1}$	13	13	0.0009 (+)
WOA	$6.284\times 10^{-1}$	$5.300\times 10^{-1}$	$2.992\times 10^{-2}$	$5.481\times 10^{-1}$	10	12	0.0121 (+)
SO	$5.499\times 10^{-1}$	$5.263\times 10^{-1}$	$6.817\times 10^{-3}$	$5.332\times 10^{-1}$	3	3	0.1500 (=)
GJO	$5.567\times 10^{-1}$	$5.300\times 10^{-1}$	$8.969\times 10^{-3}$	$5.414\times 10^{-1}$	11	10	0.0031 (+)
DBO	$1.515\times 10^{0}$	$5.371\times 10^{-1}$	$3.151\times 10^{-1}$	$7.521\times 10^{-1}$	14	14	0.0006 (+)
SCSO	$5.567\times 10^{-1}$	$5.317\times 10^{-1}$	$6.602\times 10^{-3}$	$5.385\times 10^{-1}$	12	9	0.0014 (+)
GBO	$5.418\times 10^{-1}$	$5.264\times 10^{-1}$	$5.043\times 10^{-3}$	$5.348\times 10^{-1}$	4	4	0.0420 (+)

**Table 13:** The optimal statistical results for GTCD.

Algorithm	Worst	Best	Std	Mean	Best Rank	Mean Rank	Wilcoxon
HTSO	2,963,417.47	2,963,417.47	$4.910\times 10^{-10}$	2,963,417.47	1	1	
GWO	2,963,610.71	2,963,426.36	$6.930\times 10^{1}$	2,963,510.19	10	7	0.0001 (+)
BKA	2,963,418.35	2,963,417.47	$2.790\times 10^{-1}$	2,963,417.56	2	6	0.0350 (+)
AVOA	2,984,702.07	2,963,423.59	$7.670\times 10^{3}$	2,969,084.94	9	12	0.0001 (+)
HO	2,967,359.64	2,963,683.26	$1.170\times 10^{3}$	2,964,626.59	13	10	0.0001 (+)
GTO	2,963,417.47	2,963,417.47	$4.660\times 10^{-10}$	2,963,417.47	3	2	NaN (=)
RIME	2,971,031.42	2,963,615.25	$2.330\times 10^{3}$	2,965,436.04	12	11	0.0001 (+)
SCA	2,999,760.32	2,967,143.72	$8.750\times 10^{3}$	2,978,838.20	14	13	0.0001 (+)
FTTA	2,963,417.47	2,963,417.47	$4.890\times 10^{-8}$	2,963,417.47	4	4	0.0779 (=)
WOA	3,083,150.65	2,967,901.48	$4.520\times 10^{4}$	3,025,568.03	15	14	0.0001 (+)
SO	2,963,417.49	2,963,417.47	$5.870\times 10^{-3}$	2,963,417.47	7	5	0.0001 (+)
GJO	2,966,165.60	2,963,477.73	$7.780\times 10^{2}$	2,964,006.75	11	9	0.0001 (+)
DBO	3,141,721.09	2,963,417.47	$8.000\times 10^{4}$	3,037,310.29	6	15	0.0001 (+)
SCSO	2,965,118.75	2,963,417.73	$6.470\times 10^{2}$	2,963,752.56	8	8	0.0001 (+)
GBO	2,963,417.47	2,963,417.47	$7.910\times 10^{-10}$	2,963,417.47	5	3	NaN (=)

**Table 14:** The optimal statistical results for REBD.

Algorithm	Worst	Best	Std	Mean	Best Rank	Mean Rank	Wilcoxon
HTSO	$1.461\times 10^{4}$	$1.461\times 10^{4}$	$1.819\times 10^{-12}$	$1.461\times 10^{4}$	1	1	
GWO	$1.469\times 10^{4}$	$1.464\times 10^{4}$	$1.465\times 10^{1}$	$1.467\times 10^{4}$	12	9	0.0001 (+)
BKA	$1.466\times 10^{4}$	$1.461\times 10^{4}$	$1.152\times 10^{1}$	$1.463\times 10^{4}$	7	6	0.0001 (+)
AVOA	$1.470\times 10^{4}$	$1.465\times 10^{4}$	$1.558\times 10^{1}$	$1.467\times 10^{4}$	13	10	0.0001 (+)
HO	$1.506\times 10^{4}$	$1.462\times 10^{4}$	$1.741\times 10^{2}$	$1.478\times 10^{4}$	9	12	0.0001 (+)
GTO	$1.470\times 10^{4}$	$1.461\times 10^{4}$	$4.568\times 10^{1}$	$1.466\times 10^{4}$	1	8	0.0137 (+)
RIME	$1.465\times 10^{4}$	$1.462\times 10^{4}$	$1.102\times 10^{1}$	$1.462\times 10^{4}$	8	5	0.0001 (+)
SCA	$1.582\times 10^{4}$	$1.485\times 10^{4}$	$3.205\times 10^{2}$	$1.532\times 10^{4}$	15	13	0.0001 (+)
FTTA	$1.461\times 10^{4}$	$1.461\times 10^{4}$	$2.868\times 10^{-3}$	$1.461\times 10^{4}$	1	4	0.0350 (+)
WOA	$2.026\times 10^{4}$	$1.462\times 10^{4}$	$2.465\times 10^{3}$	$1.658\times 10^{4}$	11	15	0.0001 (+)
SO	$1.461\times 10^{4}$	$1.461\times 10^{4}$	$2.186\times 10^{-12}$	$1.461\times 10^{4}$	1	1	NaN (=)
GJO	$1.474\times 10^{4}$	$1.466\times 10^{4}$	$2.658\times 10^{1}$	$1.469\times 10^{4}$	14	11	0.0001 (+)
DBO	$2.178\times 10^{4}$	$1.461\times 10^{4}$	$2.197\times 10^{3}$	$1.563\times 10^{4}$	1	14	0.0007 (+)
SCSO	$1.467\times 10^{4}$	$1.462\times 10^{4}$	$2.028\times 10^{1}$	$1.464\times 10^{4}$	10	7	0.0001 (+)
GBO	$1.461\times 10^{4}$	$1.461\times 10^{4}$	$1.050\times 10^{-12}$	$1.461\times 10^{4}$	1	1	NaN (=)

**Table 15:** The optimal statistical results for TCPD (case 2).

Algorithm	Worst	Best	Std	Mean	Best Rank	Mean Rank	Wilcoxon
HTSO	2.6586	2.6586	0.0000	2.6586	1	1	
GWO	2.9096	2.6586	0.0991	2.7252	11	4	0.0001 (+)
BKA	2.9096	2.6586	0.0978	2.7245	2	3	0.0021 (+)
AVOA	3.0988	2.6586	0.1311	2.8812	3	9	0.0002 (+)
HO	3.0038	2.6586	0.1387	2.8092	9	7	0.0001 (+)
GTO	3.6263	2.6586	0.4031	2.9289	4	11	0.0059 (+)
RIME	3.6265	2.9032	0.2813	3.2210	15	14	0.0001 (+)
SCA	3.6586	2.6765	0.2842	3.0227	13	13	0.0001 (+)
FTTA	3.6263	2.6586	0.3120	2.8956	5	10	0.0022 (+)
WOA	4.2225	2.8002	0.5263	3.2979	14	15	0.0001 (+)
SO	2.6588	2.6586	0.0001	2.6586	6	2	0.0350 (+)
GJO	2.9383	2.6586	0.1174	2.8076	12	6	0.0001 (+)
DBO	3.4251	2.6586	0.2459	2.8114	7	8	0.0021 (+)
SCSO	3.6263	2.6586	0.3040	2.7956	10	5	0.0001 (+)
GBO	3.6263	2.6586	0.3703	2.9458	8	12	0.0002 (+)

**Table 16:** The optimal statistical results for WMSR.

Algorithm	Worst	Best	Std	Mean	Best Rank	Mean Rank	Wilcoxon
HTSO	2994.2343	2994.2343	$4.287\times 10^{-13}$	2994.2343	1	1	
GWO	3021.9185	2998.1989	$7.949\times 10^{0}$	3009.9435	11	10	0.0001 (+)
BKA	3010.4438	2996.0342	$4.798\times 10^{0}$	3003.1826	9	8	0.0001 (+)
AVOA	3006.9749	2994.9841	$4.116\times 10^{0}$	2999.6140	8	7	0.0001 (+)
HO	3190.4758	3017.0226	$5.179\times 10^{1}$	3044.6753	14	13	0.0001 (+)
GTO	3007.2012	2994.2343	$4.100\times 10^{0}$	2995.5312	1	5	0.1681 (=)
RIME	3001.8952	2994.5126	$2.716\times 10^{0}$	2997.4379	7	6	0.0001 (+)
SCA	3215.1454	3057.9125	$5.431\times 10^{1}$	3148.4351	15	15	0.0001 (+)
FTTA	2994.2343	2994.2343	$9.601\times 10^{-12}$	2994.2343	1	3	0.3681 (=)
WOA	3237.7881	3011.0992	$7.571\times 10^{1}$	3114.3360	12	14	0.0001 (+)
SO	2994.2343	2994.2343	$2.382\times 10^{-9}$	2994.2343	1	4	0.0149 (+)
GJO	3067.8792	3012.7117	$1.673\times 10^{1}$	3024.5075	13	12	0.0001 (+)
DBO	3056.0001	2994.2343	$2.031\times 10^{1}$	3022.1867	1	11	0.0002 (+)
SCSO	3013.4992	2997.5293	$4.174\times 10^{0}$	3005.3711	10	9	0.0001 (+)
GBO	2994.2343	2994.2343	$2.144\times 10^{-13}$	2994.2343	1	1	NaN (=)

**Table 17:** The optimal statistical results for 10-BTD.

Algorithm	Worst	Best	Std	Mean	Best Rank	Mean Rank	Wilcoxon
HTSO	530.4449	524.2426	2.5766	525.5232	1	1	
GWO	531.1260	524.5555	2.9399	527.4776	5	2	0.0046 (+)
BKA	533.8079	524.4282	2.5733	530.0889	3	6	0.0010 (+)
AVOA	537.7833	525.4331	4.3211	533.4783	8	10	0.0006 (+)
HO	561.7978	527.7440	12.0732	543.3590	12	12	0.0006 (+)
GTO	533.1952	524.3261	3.4568	528.0310	2	3	0.0073 (+)
RIME	579.8131	528.3345	17.6696	543.9313	13	13	0.0006 (+)
SCA	617.3244	541.3110	20.3069	577.6997	14	14	0.0002 (+)
FTTA	538.2751	524.4825	4.0905	530.7314	4	8	0.0013 (+)
WOA	850.1855	597.4614	74.5843	727.7377	15	15	0.0002 (+)
SO	531.5750	525.2072	2.8163	528.8129	7	4	0.0017 (+)
GJO	554.9732	525.6637	8.7110	531.3003	9	9	0.0046 (+)
DBO	590.3821	526.4208	20.5509	542.0578	11	11	0.0010 (+)
SCSO	536.1271	525.7112	3.0931	530.1389	10	7	0.0028 (+)
GBO	536.8823	524.7621	3.8056	529.3479	6	5	0.0022 (+)

**Table 18:** The optimal statistical results for model of mountain No. 1.

Algorithm	Worst	Best	Std	Mean	Best Rank	Mean Rank	Wilcoxon
HTSO	$2.334\times 10^{2}$	$1.753\times 10^{2}$	$1.587\times 10^{1}$	$1.909\times 10^{2}$	1	1	
GJO	$6.348\times 10^{2}$	$1.916\times 10^{2}$	$1.377\times 10^{2}$	$2.727\times 10^{2}$	9	10	0.0013 (+)
DBO	$3.154\times 10^{2}$	$1.984\times 10^{2}$	$4.563\times 10^{1}$	$2.367\times 10^{2}$	13	12	0.0010 (+)
FTTA	$3.620\times 10^{2}$	$1.838\times 10^{2}$	$5.761\times 10^{1}$	$2.204\times 10^{2}$	5	4	0.1212 (=)
BKA	$2.661\times 10^{2}$	$1.910\times 10^{2}$	$2.844\times 10^{1}$	$2.094\times 10^{2}$	8	5	0.0028 (+)
WOA	$3.608\times 10^{2}$	$1.984\times 10^{2}$	$7.229\times 10^{1}$	$2.821\times 10^{2}$	12	14	0.0004 (+)
HHO	$1.000\times 10^{33}$	$1.799\times 10^{2}$	$4.216\times 10^{32}$	$2.000\times 10^{32}$	3	13	0.0113 (+)
SMA	$2.582\times 10^{2}$	$1.861\times 10^{2}$	$2.994\times 10^{1}$	$2.129\times 10^{2}$	6	3	0.0757 (=)
SSA	$3.245\times 10^{2}$	$1.780\times 10^{2}$	$5.232\times 10^{1}$	$2.261\times 10^{2}$	2	2	0.2730 (=)
SCA	$9.590\times 10^{2}$	$2.377\times 10^{2}$	$2.268\times 10^{2}$	$3.946\times 10^{2}$	15	15	0.0002 (+)
SCSO	$1.000\times 10^{33}$	$2.005\times 10^{2}$	$3.162\times 10^{32}$	$1.000\times 10^{32}$	14	11	0.0017 (+)
MFO	$5.388\times 10^{2}$	$1.936\times 10^{2}$	$1.007\times 10^{2}$	$2.992\times 10^{2}$	10	9	0.0004 (+)
GWO	$1.000\times 10^{33}$	$1.900\times 10^{2}$	$3.162\times 10^{32}$	$1.000\times 10^{32}$	7	6	0.0058 (+)
SO	$3.221\times 10^{2}$	$1.820\times 10^{2}$	$4.710\times 10^{1}$	$2.757\times 10^{2}$	4	8	0.0017 (+)
RIME	$3.325\times 10^{2}$	$1.955\times 10^{2}$	$5.520\times 10^{1}$	$2.523\times 10^{2}$	11	7	0.0008 (+)

**Table 19:** The optimal statistical results for model of Mountain No. 2.

Algorithm	Worst	Best	Std	Mean	Best Rank	Mean Rank	Wilcoxon
HTSO	$1.874\times 10^{2}$	$1.771\times 10^{2}$	$3.686\times 10^{0}$	$1.808\times 10^{2}$	1	1	
GJO	$2.232\times 10^{2}$	$1.827\times 10^{2}$	$1.355\times 10^{1}$	$1.980\times 10^{2}$	9	6	0.0006 (+)
DBO	$2.465\times 10^{2}$	$1.800\times 10^{2}$	$2.199\times 10^{1}$	$2.062\times 10^{2}$	4	9	0.0013 (+)
FTTA	$2.060\times 10^{2}$	$1.778\times 10^{2}$	$7.737\times 10^{0}$	$1.930\times 10^{2}$	2	4	0.0017 (+)
BKA	$2.096\times 10^{2}$	$1.809\times 10^{2}$	$9.362\times 10^{0}$	$1.945\times 10^{2}$	5	5	0.0010 (+)
WOA	$2.799\times 10^{2}$	$2.077\times 10^{2}$	$2.286\times 10^{1}$	$2.292\times 10^{2}$	14	13	0.0002 (+)
HHO	$2.883\times 10^{2}$	$1.859\times 10^{2}$	$3.504\times 10^{1}$	$2.116\times 10^{2}$	11	11	0.0004 (+)
SMA	$2.072\times 10^{2}$	$1.821\times 10^{2}$	$9.512\times 10^{0}$	$1.927\times 10^{2}$	8	3	0.0017 (+)
SSA	$1.979\times 10^{2}$	$1.788\times 10^{2}$	$6.520\times 10^{0}$	$1.899\times 10^{2}$	3	2	0.0028 (+)
SCA	$3.100\times 10^{2}$	$2.122\times 10^{2}$	$3.158\times 10^{1}$	$2.526\times 10^{2}$	15	14	0.0002 (+)
SCSO	$3.072\times 10^{2}$	$1.942\times 10^{2}$	$3.289\times 10^{1}$	$2.208\times 10^{2}$	13	12	0.0002 (+)
MFO	$2.305\times 10^{2}$	$1.815\times 10^{2}$	$1.756\times 10^{1}$	$2.072\times 10^{2}$	6	10	0.0008 (+)
GWO	$1.067\times 10^{3}$	$1.819\times 10^{2}$	$2.783\times 10^{2}$	$2.753\times 10^{2}$	7	15	0.0036 (+)
SO	$2.330\times 10^{2}$	$1.863\times 10^{2}$	$1.246\times 10^{1}$	$2.029\times 10^{2}$	12	8	0.0002 (+)
RIME	$2.109\times 10^{2}$	$1.827\times 10^{2}$	$9.108\times 10^{0}$	$1.988\times 10^{2}$	10	7	0.0003 (+)

**Table 20:** The optimal statistical results for model of Mountain No. 3.

Algorithm	Worst	Best	Std	Mean	Best Rank	Mean Rank	Wilcoxon
HTSO	$7.894\times 10^{2}$	$1.892\times 10^{2}$	$1.893\times 10^{2}$	$2.506\times 10^{2}$	1	1	
GJO	$1.000\times 10^{33}$	$1.950\times 10^{2}$	$3.162\times 10^{32}$	$1.000\times 10^{32}$	5	11	0.0022 (+)
DBO	$1.159\times 10^{3}$	$1.981\times 10^{2}$	$3.598\times 10^{2}$	$4.157\times 10^{2}$	7	4	0.0013 (+)
FTTA	$8.546\times 10^{2}$	$1.918\times 10^{2}$	$3.031\times 10^{2}$	$5.337\times 10^{2}$	3	7	0.0017 (+)
BKA	$8.161\times 10^{2}$	$1.925\times 10^{2}$	$2.129\times 10^{2}$	$4.640\times 10^{2}$	4	6	0.0022 (+)
WOA	$1.000\times 10^{33}$	$1.983\times 10^{2}$	$4.216\times 10^{32}$	$2.000\times 10^{32}$	8	12	0.0004 (+)
HHO	$1.000\times 10^{33}$	$1.985\times 10^{2}$	$5.271\times 10^{32}$	$5.000\times 10^{32}$	9	15	0.0005 (+)
SMA	$7.980\times 10^{2}$	$7.904\times 10^{2}$	$2.510\times 10^{0}$	$7.933\times 10^{2}$	15	9	0.0002 (+)
SSA	$1.504\times 10^{3}$	$5.207\times 10^{2}$	$2.816\times 10^{2}$	$8.858\times 10^{2}$	14	10	0.0003 (+)
SCA	$4.105\times 10^{2}$	$2.240\times 10^{2}$	$5.181\times 10^{1}$	$2.886\times 10^{2}$	12	2	0.0028 (+)
SCSO	$1.000\times 10^{33}$	$2.039\times 10^{2}$	$4.831\times 10^{32}$	$3.000\times 10^{32}$	10	14	0.0013 (+)
MFO	$1.202\times 10^{3}$	$5.206\times 10^{2}$	$2.189\times 10^{2}$	$6.424\times 10^{2}$	13	8	0.0017 (+)
GWO	$1.000\times 10^{33}$	$1.899\times 10^{2}$	$4.216\times 10^{32}$	$2.000\times 10^{32}$	2	13	0.0113 (+)
SO	$6.133\times 10^{2}$	$2.075\times 10^{2}$	$1.394\times 10^{2}$	$4.578\times 10^{2}$	11	5	0.0028 (+)
RIME	$6.061\times 10^{2}$	$1.977\times 10^{2}$	$1.690\times 10^{2}$	$3.081\times 10^{2}$	6	3	0.0028 (+)

**Table 21:** The optimal statistical results for model of Mountain No. 4.

Algorithm	Worst	Best	Std	Mean	Best Rank	Mean Rank	Wilcoxon
HTSO	$3.798\times 10^{2}$	$1.922\times 10^{2}$	$9.318\times 10^{1}$	$2.674\times 10^{2}$	1	1	
GJO	$4.899\times 10^{2}$	$2.295\times 10^{2}$	$7.716\times 10^{1}$	$3.956\times 10^{2}$	6	10	0.0046 (+)
DBO	$5.070\times 10^{2}$	$3.059\times 10^{2}$	$7.551\times 10^{1}$	$4.084\times 10^{2}$	13	11	0.0257 (+)
FTTA	$3.976\times 10^{2}$	$2.889\times 10^{2}$	$3.706\times 10^{1}$	$3.208\times 10^{2}$	9	5	0.2730 (=)
BKA	$3.871\times 10^{2}$	$2.937\times 10^{2}$	$2.881\times 10^{1}$	$3.185\times 10^{2}$	12	4	0.3075 (=)
WOA	$4.603\times 10^{2}$	$3.207\times 10^{2}$	$4.622\times 10^{1}$	$3.903\times 10^{2}$	14	9	0.0058 (+)
HHO	$1.000\times 10^{33}$	$2.900\times 10^{2}$	$4.216\times 10^{32}$	$2.000\times 10^{32}$	10	14	0.0113 (+)
SMA	$4.103\times 10^{2}$	$1.980\times 10^{2}$	$6.192\times 10^{1}$	$2.927\times 10^{2}$	2	2	0.3447 (=)
SSA	$4.007\times 10^{2}$	$2.831\times 10^{2}$	$3.906\times 10^{1}$	$3.222\times 10^{2}$	7	6	0.1859 (=)
SCA	$1.880\times 10^{3}$	$3.576\times 10^{2}$	$4.387\times 10^{2}$	$6.468\times 10^{2}$	15	12	0.0006 (+)
SCSO	$1.000\times 10^{33}$	$2.931\times 10^{2}$	$3.162\times 10^{32}$	$1.000\times 10^{32}$	11	13	0.0017 (+)
MFO	$4.488\times 10^{2}$	$1.982\times 10^{2}$	$9.539\times 10^{1}$	$3.460\times 10^{2}$	3	8	0.0640 (=)
GWO	$1.000\times 10^{33}$	$2.041\times 10^{2}$	$4.216\times 10^{32}$	$2.000\times 10^{32}$	4	15	0.0013 (+)
SO	$3.819\times 10^{2}$	$2.209\times 10^{2}$	$4.483\times 10^{1}$	$3.097\times 10^{2}$	5	3	0.3075 (=)
RIME	$3.851\times 10^{2}$	$2.874\times 10^{2}$	$3.708\times 10^{1}$	$3.310\times 10^{2}$	8	7	0.1620 (=)

**Table 22:** The optimal statistical results for model of Mountain No. 5.

Algorithm	Worst	Best	Std	Mean	Best Rank	Mean Rank	Wilcoxon
HTSO	$4.926\times 10^{2}$	$1.932\times 10^{2}$	$9.271\times 10^{1}$	$2.955\times 10^{2}$	1	1	
GJO	$1.489\times 10^{3}$	$2.070\times 10^{2}$	$3.555\times 10^{2}$	$4.979\times 10^{2}$	6	11	0.0113 (+)
DBO	$7.536\times 10^{2}$	$2.138\times 10^{2}$	$1.493\times 10^{2}$	$4.582\times 10^{2}$	9	10	0.0073 (+)
FTTA	$5.984\times 10^{2}$	$2.925\times 10^{2}$	$1.053\times 10^{2}$	$4.240\times 10^{2}$	11	9	0.0091 (+)
BKA	$4.696\times 10^{2}$	$2.018\times 10^{2}$	$8.815\times 10^{1}$	$3.412\times 10^{2}$	4	5	0.1041 (=)
WOA	$5.088\times 10^{2}$	$3.291\times 10^{2}$	$6.219\times 10^{1}$	$4.045\times 10^{2}$	14	7	0.0073 (+)
HHO	$1.000\times 10^{33}$	$2.134\times 10^{2}$	$5.271\times 10^{32}$	$5.000\times 10^{32}$	8	15	0.0069 (+)
SMA	$3.623\times 10^{2}$	$2.926\times 10^{2}$	$1.949\times 10^{1}$	$3.241\times 10^{2}$	12	4	0.0640 (=)
SSA	$4.987\times 10^{2}$	$2.971\times 10^{2}$	$6.265\times 10^{1}$	$3.206\times 10^{2}$	13	3	0.1212 (=)
SCA	$1.476\times 10^{3}$	$3.792\times 10^{2}$	$3.141\times 10^{2}$	$6.534\times 10^{2}$	15	13	0.0003 (+)
SCSO	$9.399\times 10^{2}$	$2.450\times 10^{2}$	$2.219\times 10^{2}$	$5.028\times 10^{2}$	10	12	0.0113 (+)
MFO	$5.550\times 10^{2}$	$2.119\times 10^{2}$	$1.204\times 10^{2}$	$3.927\times 10^{2}$	7	6	0.0452 (+)
GWO	$1.000\times 10^{33}$	$1.987\times 10^{2}$	$4.216\times 10^{32}$	$2.000\times 10^{32}$	3	14	0.0311 (+)
SO	$4.999\times 10^{2}$	$1.973\times 10^{2}$	$9.894\times 10^{1}$	$4.145\times 10^{2}$	2	8	0.0140 (+)
RIME	$4.415\times 10^{2}$	$2.030\times 10^{2}$	$7.112\times 10^{1}$	$3.198\times 10^{2}$	5	2	0.1212 (=)

**Table 23:** The optimal statistical results for model of Mountain No. 6.

Algorithm	Worst	Best	Std	Mean	Best Rank	Mean Rank	Wilcoxon
HTSO	$3.828\times 10^{2}$	$2.541\times 10^{2}$	$4.883\times 10^{1}$	$3.306\times 10^{2}$	1	1	
GJO	$1.606\times 10^{3}$	$3.830\times 10^{2}$	$3.944\times 10^{2}$	$5.987\times 10^{2}$	13	11	0.0002 (+)
DBO	$7.444\times 10^{2}$	$3.303\times 10^{2}$	$1.595\times 10^{2}$	$4.451\times 10^{2}$	7	6	0.0757 (=)
FTTA	$5.859\times 10^{2}$	$3.610\times 10^{2}$	$8.415\times 10^{1}$	$4.528\times 10^{2}$	10	8	0.0010 (+)
BKA	$6.062\times 10^{2}$	$3.326\times 10^{2}$	$9.045\times 10^{1}$	$4.368\times 10^{2}$	8	5	0.0539 (=)
WOA	$6.192\times 10^{2}$	$3.354\times 10^{2}$	$9.431\times 10^{1}$	$4.475\times 10^{2}$	9	7	0.0017 (+)
HHO	$1.000\times 10^{33}$	$3.995\times 10^{2}$	$4.831\times 10^{32}$	$3.000\times 10^{32}$	14	14	0.0002 (+)
SMA	$5.395\times 10^{2}$	$3.125\times 10^{2}$	$8.863\times 10^{1}$	$4.073\times 10^{2}$	5	3	0.1405 (=)
SSA	$4.701\times 10^{2}$	$2.981\times 10^{2}$	$6.050\times 10^{1}$	$3.351\times 10^{2}$	3	2	0.9097 (=)
SCA	$9.628\times 10^{2}$	$4.441\times 10^{2}$	$1.775\times 10^{2}$	$6.748\times 10^{2}$	15	12	0.0002 (+)
SCSO	$1.000\times 10^{33}$	$3.048\times 10^{2}$	$4.216\times 10^{32}$	$2.000\times 10^{32}$	4	13	0.0172 (+)
MFO	$7.733\times 10^{2}$	$2.578\times 10^{2}$	$1.464\times 10^{2}$	$4.893\times 10^{2}$	2	10	0.0140 (+)
GWO	$1.000\times 10^{33}$	$3.691\times 10^{2}$	$4.831\times 10^{32}$	$3.000\times 10^{32}$	11	15	0.0002 (+)
SO	$5.207\times 10^{2}$	$3.693\times 10^{2}$	$4.715\times 10^{1}$	$4.571\times 10^{2}$	12	9	0.0003 (+)
RIME	$5.154\times 10^{2}$	$3.299\times 10^{2}$	$6.885\times 10^{1}$	$4.347\times 10^{2}$	6	4	0.0073 (+)

**Table 24:** The optimal statistical results for model of Mountain No. 7.

Algorithm	Worst	Best	Std	Mean	Best Rank	Mean Rank	Wilcoxon
HTSO	$5.046\times 10^{2}$	$4.890\times 10^{2}$	$4.554\times 10^{0}$	$4.919\times 10^{2}$	1	1	
GJO	$5.949\times 10^{2}$	$5.009\times 10^{2}$	$2.781\times 10^{1}$	$5.228\times 10^{2}$	10	4	0.0002 (+)
DBO	$1.015\times 10^{3}$	$5.159\times 10^{2}$	$1.740\times 10^{2}$	$6.706\times 10^{2}$	12	9	0.0002 (+)
FTTA	$7.807\times 10^{2}$	$4.918\times 10^{2}$	$8.840\times 10^{1}$	$5.536\times 10^{2}$	4	6	0.0006 (+)
BKA	$5.966\times 10^{2}$	$4.932\times 10^{2}$	$3.398\times 10^{1}$	$5.449\times 10^{2}$	5	5	0.0003 (+)
WOA	$1.000\times 10^{33}$	$5.623\times 10^{2}$	$4.216\times 10^{32}$	$2.000\times 10^{32}$	14	13	0.0002 (+)
HHO	$1.000\times 10^{33}$	$5.429\times 10^{2}$	$5.164\times 10^{32}$	$4.000\times 10^{32}$	13	15	0.0002 (+)
SMA	$5.058\times 10^{2}$	$4.896\times 10^{2}$	$5.882\times 10^{0}$	$4.956\times 10^{2}$	3	2	0.1212 (=)
SSA	$5.356\times 10^{2}$	$4.892\times 10^{2}$	$1.987\times 10^{1}$	$5.037\times 10^{2}$	2	3	0.4727 (=)
SCA	$9.877\times 10^{2}$	$6.348\times 10^{2}$	$1.191\times 10^{2}$	$8.040\times 10^{2}$	15	11	0.0002 (+)
SCSO	$1.452\times 10^{3}$	$5.010\times 10^{2}$	$3.191\times 10^{2}$	$7.636\times 10^{2}$	11	10	0.0002 (+)
MFO	$1.482\times 10^{3}$	$5.004\times 10^{2}$	$3.755\times 10^{2}$	$8.887\times 10^{2}$	9	12	0.0002 (+)
GWO	$1.000\times 10^{33}$	$4.946\times 10^{2}$	$4.216\times 10^{32}$	$2.000\times 10^{32}$	6	14	0.0004 (+)
SO	$7.434\times 10^{2}$	$4.989\times 10^{2}$	$9.094\times 10^{1}$	$5.978\times 10^{2}$	8	7	0.0003 (+)
RIME	$9.444\times 10^{2}$	$4.950\times 10^{2}$	$1.652\times 10^{2}$	$6.653\times 10^{2}$	7	8	0.0003 (+)

## Data Availability

All relevant data are within the paper.

## Supplementary Materials

The following supporting information can be downloaded at https://www.mdpi.com/article/10.3390/biomimetics11030191/s1: Table S1: The Experimental Results of the CEC Test Set; Table S2: The Results of Wilcoxon Rank Sum Test for the CEC Test Set; Table S3: Ablation Experiments; Table S4: Sensitivity Analysis.

## Appendix A. The Experimental Results of the CEC Test Set

Table S1 presents the experimental results of 22 algorithms on the CEC benchmark sets, including CEC-2017 with 30, 50, and 100 dimensions; CEC-2020 with 10, 15, and 20 dimensions; and CEC-2022 with 10 and 20 dimensions. The names of the benchmark sets and their corresponding dimensions are indicated in the names of the worksheets at the bottom of the file. To visually highlight the algorithms with the best performance, the minimum value in each row is shown in bold.

## Appendix B. The Results of Wilcoxon Rank Sum Test for the CEC Test Set

Table S2 presents the results of the Wilcoxon rank-sum test between HTSO and the other 21 comparison algorithms, based on the experimental data in Table S1. The names of the benchmark sets and their corresponding dimensions are likewise indicated in the worksheet names at the bottom of the file.

## Appendix C. Ablation Experiments

Table S3 consists of two tables. The first reports the experimental results of HTSO, HTSO-α, and HTSO-β on the 30-dimensional CEC-2017 benchmark suite, while the second presents the Wilcoxon rank-sum test results comparing HTSO against HTSO-α and HTSO-β.

## Appendix D. Sensitivity Analysis

Table S4 presents the results of the sensitivity analysis, specifically detailing the average performance on the 30-dimensional CEC-2017 benchmark suite across CT threshold values of 0.3, 0.4, 0.5, 0.6, and 0.7.

## References

[1] Yang X.S. (2010). Engineering Optimization: An Introduction with Metaheuristic Applications.

[2] Cavazzuti M. (2012). Deterministic optimization. Optimization Methods: From Theory to Design Scientific and Technological Aspects in Mechanics.

[3] Powell W.B. (2019). A unified framework for stochastic optimization. Eur. J. Oper. Res..

[4] Dantzig G.B. (2002). Linear programming. Oper. Res..

[5] Bellman R.E., Dreyfus S.E. (2015). Applied Dynamic Programming.

[6] Ruder S. (2016). An overview of gradient descent optimization algorithms. arXiv.

[7] Kelley C.T. (2003). Solving Nonlinear Equations with Newton’s Method.

[8] Dokeroglu T., Sevinc E., Kucukyilmaz T., Cosar A. (2019). A survey on new generation metaheuristic algorithms. Comput. Ind. Eng..

[9] Lian J., Hui G., Ma L., Zhu T., Wu X., Heidari A.A., Chen Y., Chen H. (2024). Parrot optimizer: Algorithm and applications to medical problems. Comput. Biol. Med..

[10] Gülmez B. (2023). Stock price prediction with optimized deep LSTM network with artificial rabbits optimization algorithm. Expert Syst. Appl..

[11] Mayer M.J., Szilágyi A., Gróf G. (2020). Environmental and economic multi-objective optimization of a household level hybrid renewable energy system by genetic algorithm. Appl. Energy.

[12] Houssein E.H., Helmy B.E.d., Oliva D., Elngar A.A., Shaban H. (2021). A novel black widow optimization algorithm for multilevel thresholding image segmentation. Expert Syst. Appl..

[13] Li J., Song Z., Wang X., Wang Y., Jia Y. (2022). A novel offshore wind farm typhoon wind speed prediction model based on PSO–Bi-LSTM improved by VMD. Energy.

[14] Mirjalili S. (2015). How effective is the Grey Wolf optimizer in training multi-layer perceptrons. Appl. Intell..

[15] Houssein E.H., Abdelkareem D.A., Emam M.M., Hameed M.A., Younan M. (2022). An efficient image segmentation method for skin cancer imaging using improved golden jackal optimization algorithm. Comput. Biol. Med..

[16] Oloyede M.O., Hancke G.P., Myburgh H.C. (2018). Improving face recognition systems using a new image enhancement technique, hybrid features and the convolutional neural network. IEEE Access.

[17] Fathollahi-Fard A.M., Woodward L., Akhrif O. (2024). A distributed permutation flow-shop considering sustainability criteria and real-time scheduling. J. Ind. Inf. Integr..

[18] Anosri S., Panagant N., Champasak P., Bureerat S., Thipyopas C., Kumar S., Pholdee N., Yıldız B.S., Yildiz A.R. (2023). A comparative study of state-of-the-art metaheuristics for solving many-objective optimization problems of fixed wing unmanned aerial vehicle conceptual design. Arch. Comput. Methods Eng..

[19] Phung M.D., Ha Q.P. (2021). Safety-enhanced UAV path planning with spherical vector-based particle swarm optimization. Appl. Soft Comput..

[20] Shabbar R., Kasasbeh A., Ahmed M.M. (2021). Charging station allocation for electric vehicle network using stochastic modeling and grey wolf optimization. Sustainability.

[21] Liu H., Basem A., Jasim D.J., Hashemian M., Eftekhari S.A., Al-fanhrawi H.J., Abdullaeva B., Salahshour S. (2025). Multi-objective optimization of buckling load and natural frequency in functionally graded porous nanobeams using non-dominated sorting genetic Algorithm-II. Eng. Appl. Artif. Intell..

[22] Ghobaei-Arani M., Shahidinejad A. (2022). A cost-efficient IoT service placement approach using whale optimization algorithm in fog computing environment. Expert Syst. Appl..

[23] Gaspar J., Loja M., Barbosa J. (2024). Metaheuristic optimization of functionally graded 2D and 3D discrete structures using the red fox algorithm. J. Compos. Sci..

[24] Blum C. (2005). Ant colony optimization: Introduction and recent trends. Phys. Life Rev..

[25] Jain M., Saihjpal V., Singh N., Singh S.B. (2022). An overview of variants and advancements of PSO algorithm. Appl. Sci..

[26] Yang X.S., Deb S. (2014). Cuckoo search: Recent advances and applications. Neural Comput. Appl..

[27] Yang X.S. (2010). A new metaheuristic bat-inspired algorithm. Nature Inspired Cooperative Strategies for Optimization (NICSO 2010).

[28] Li Y., Lin X., Liu J. (2021). An improved gray wolf optimization algorithm to solve engineering problems. Sustainability.

[29] Mirjalili S. (2015). Moth-flame optimization algorithm: A novel nature-inspired heuristic paradigm. Knowl. Based Syst..

[30] Mirjalili S., Lewis A. (2016). The whale optimization algorithm. Adv. Eng. Softw..

[31] Heidari A.A., Mirjalili S., Faris H., Aljarah I., Mafarja M., Chen H. (2019). Harris hawks optimization: Algorithm and applications. Future Gener. Comput. Syst..

[32] Xue J., Shen B. (2020). A novel swarm intelligence optimization approach: Sparrow search algorithm. Syst. Sci. Control. Eng..

[33] Faramarzi A., Heidarinejad M., Mirjalili S., Gandomi A.H. (2020). Marine Predators Algorithm: A nature-inspired metaheuristic. Expert Syst. Appl..

[34] Li S., Chen H., Wang M., Heidari A.A., Mirjalili S. (2020). Slime mould algorithm: A new method for stochastic optimization. Future Gener. Comput. Syst..

[35] Abdollahzadeh B., Gharehchopogh F.S., Mirjalili S. (2021). African vultures optimization algorithm: A new nature-inspired metaheuristic algorithm for global optimization problems. Comput. Ind. Eng..

[36] Abdollahzadeh B., Soleimanian Gharehchopogh F., Mirjalili S. (2021). Artificial gorilla troops optimizer: A new nature-inspired metaheuristic algorithm for global optimization problems. Int. J. Intell. Syst..

[37] Abualigah L., Yousri D., Abd Elaziz M., Ewees A.A., Al-Qaness M.A., Gandomi A.H. (2021). Aquila optimizer: A novel meta-heuristic optimization algorithm. Comput. Ind. Eng..

[38] Chopra N., Ansari M.M. (2022). Golden jackal optimization: A novel nature-inspired optimizer for engineering applications. Expert Syst. Appl..

[39] Hashim F.A., Hussien A.G. (2022). Snake Optimizer: A novel meta-heuristic optimization algorithm. Knowl. Based Syst..

[40] Wang L., Cao Q., Zhang Z., Mirjalili S., Zhao W. (2022). Artificial rabbits optimization: A new bio-inspired meta-heuristic algorithm for solving engineering optimization problems. Eng. Appl. Artif. Intell..

[41] Hashim F.A., Houssein E.H., Hussain K., Mabrouk M.S., Al-Atabany W. (2022). Honey Badger Algorithm: New metaheuristic algorithm for solving optimization problems. Math. Comput. Simul..

[42] Trojovská E., Dehghani M., Trojovskỳ P. (2022). Zebra optimization algorithm: A new bio-inspired optimization algorithm for solving optimization algorithm. IEEE Access.

[43] Xue J., Shen B. (2023). Dung beetle optimizer: A new meta-heuristic algorithm for global optimization. J. Supercomput..

[44] Abdel-Basset M., Mohamed R., Jameel M., Abouhawwash M. (2023). Nutcracker optimizer: A novel nature-inspired metaheuristic algorithm for global optimization and engineering design problems. Knowl. Based Syst..

[45] Seyyedabbasi A., Kiani F. (2023). Sand Cat swarm optimization: A nature-inspired algorithm to solve global optimization problems. Eng. Comput..

[46] Wang J., Wang W.C., Hu X.X., Qiu L., Zang H.F. (2024). Black-winged kite algorithm: A nature-inspired meta-heuristic for solving benchmark functions and engineering problems. Artif. Intell. Rev..

[47] Fu Y., Liu D., Chen J., He L. (2024). Secretary bird optimization algorithm: A new metaheuristic for solving global optimization problems. Artif. Intell. Rev..

[48] Abdel-Basset M., Mohamed R., Abouhawwash M. (2024). Crested Porcupine Optimizer: A new nature-inspired metaheuristic. Knowl. Based Syst..

[49] Amiri M.H., Mehrabi Hashjin N., Montazeri M., Mirjalili S., Khodadadi N. (2024). Hippopotamus optimization algorithm: A novel nature-inspired optimization algorithm. Sci. Rep..

[50] Yao X., Liu Y., Lin G. (1999). Evolutionary programming made faster. IEEE Trans. Evol. Comput..

[51] Mirjalili S., Mirjalili S. (2019). Genetic algorithm. Evolutionary Algorithms and Neural Networks: Theory and Applications.

[52] Koza J.R. (1994). Genetic programming as a means for programming computers by natural selection. Stat. Comput..

[53] Feoktistov V. (2006). Differential Evolution.

[54] Simon D. (2008). Biogeography-based optimization. IEEE Trans. Evol. Comput..

[55] Ghaemi M., Feizi-Derakhshi M.R. (2014). Forest optimization algorithm. Expert Syst. Appl..

[56] Kiran M.S. (2015). TSA: Tree-seed algorithm for continuous optimization. Expert Syst. Appl..

[57] Huang G. (2016). Artificial infectious disease optimization: A SEIQR epidemic dynamic model-based function optimization algorithm. Swarm Evol. Comput..

[58] Hayyolalam V., Kazem A.A.P. (2020). Black widow optimization algorithm: A novel meta-heuristic approach for solving engineering optimization problems. Eng. Appl. Artif. Intell..

[59] Alnahwi F.M., Al-Yasir Y.I., Sattar D., Ali R.S., See C.H., Abd-Alhameed R.A. (2021). A new optimization algorithm based on the fungi Kingdom expansion behavior for antenna applications. Electronics.

[60] Verij kazemi M., Veysari E.F. (2022). A new optimization algorithm inspired by the quest for the evolution of human society: Human felicity algorithm. Expert Syst. Appl..

[61] Abdel-Basset M., Mohamed R., Abouhawwash M. (2025). Fungal growth optimizer: A novel nature-inspired metaheuristic algorithm for stochastic optimization. Comput. Methods Appl. Mech. Eng..

[62] Van Laarhoven P.J., Aarts E.H., van Laarhoven P.J., Aarts E.H. (1987). Simulated Annealing.

[63] Erol O.K., Eksin I. (2006). A new optimization method: Big bang–big crunch. Adv. Eng. Softw..

[64] Formato R. (2007). Central force optimization: A new metaheuristic with applications in applied electromagnetics. Prog. Electromagn. Res..

[65] Shah-Hosseini H. (2009). The intelligent water drops algorithm: A nature-inspired swarm-based optimization algorithm. Int. J.-Bio-Inspired Comput..

[66] Eskandar H., Sadollah A., Bahreininejad A., Hamdi M. (2012). Water cycle algorithm—A novel metaheuristic optimization method for solving constrained engineering optimization problems. Comput. Struct..

[67] Abualigah L. (2020). Multi-verse optimizer algorithm: A comprehensive survey of its results, variants, and applications. Neural Comput. Appl..

[68] Ghasemi M., Davoudkhani I.F., Akbari E., Rahimnejad A., Ghavidel S., Li L. (2020). A novel and effective optimization algorithm for global optimization and its engineering applications: Turbulent Flow of Water-based Optimization (TFWO). Eng. Appl. Artif. Intell..

[69] Faramarzi A., Heidarinejad M., Stephens B., Mirjalili S. (2020). Equilibrium optimizer: A novel optimization algorithm. Knowl. Based Syst..

[70] Pan Q., Tang J., Lao S. (2022). EDOA: An elastic deformation optimization algorithm. Appl. Intell..

[71] Mahdavi-Meymand A., Zounemat-Kermani M. (2022). Homonuclear molecules optimization (HMO) meta-heuristic algorithm. Knowl. Based Syst..

[72] Abdel-Basset M., Mohamed R., Azeem S.A.A., Jameel M., Abouhawwash M. (2023). Kepler optimization algorithm: A new metaheuristic algorithm inspired by Kepler’s laws of planetary motion. Knowl. Based Syst..

[73] Hashim F.A., Mostafa R.R., Hussien A.G., Mirjalili S., Sallam K.M. (2023). Fick’s Law Algorithm: A physical law-based algorithm for numerical optimization. Knowl. Based Syst..

[74] Su H., Zhao D., Heidari A.A., Liu L., Zhang X., Mafarja M., Chen H. (2023). RIME: A physics-based optimization. Neurocomputing.

[75] Guan Z., Ren C., Niu J., Wang P., Shang Y. (2023). Great Wall Construction Algorithm: A novel meta-heuristic algorithm for engineer problems. Expert Syst. Appl..

[76] Ghasemi M., Zare M., Zahedi A., Akbari M.A., Mirjalili S., Abualigah L. (2024). Geyser inspired algorithm: A new geological-inspired meta-heuristic for real-parameter and constrained engineering optimization. J. Bionic Eng..

[77] Sowmya R., Premkumar M., Jangir P. (2024). Newton-Raphson-based optimizer: A new population-based metaheuristic algorithm for continuous optimization problems. Eng. Appl. Artif. Intell..

[78] Mirjalili S. (2016). SCA: A sine cosine algorithm for solving optimization problems. Knowl. Based Syst..

[79] Tanyildizi E., Demir G. (2017). Golden sine algorithm: A novel math-inspired algorithm. Adv. Electr. Comput. Eng..

[80] Ahmadianfar I., Bozorg-Haddad O., Chu X. (2020). Gradient-based optimizer: A new metaheuristic optimization algorithm. Inf. Sci..

[81] Abualigah L., Diabat A., Mirjalili S., Abd Elaziz M., Gandomi A.H. (2021). The arithmetic optimization algorithm. Comput. Methods Appl. Mech. Eng..

[82] Qais M.H., Hasanien H.M., Turky R.A., Alghuwainem S., Tostado-Véliz M., Jurado F. (2022). Circle search algorithm: A geometry-based metaheuristic optimization algorithm. Mathematics.

[83] Zhao W., Wang L., Zhang Z., Mirjalili S., Khodadadi N., Ge Q. (2023). Quadratic Interpolation Optimization (QIO): A new optimization algorithm based on generalized quadratic interpolation and its applications to real-world engineering problems. Comput. Methods Appl. Mech. Eng..

[84] Trojovskỳ P., Dehghani M. (2023). Subtraction-average-based optimizer: A new swarm-inspired metaheuristic algorithm for solving optimization problems. Biomimetics.

[85] Bai J., Li Y., Zheng M., Khatir S., Benaissa B., Abualigah L., Wahab M.A. (2023). A sinh cosh optimizer. Knowl. Based Syst..

[86] Glover F., Laguna M. (1998). Tabu Search.

[87] Rao R.V., Savsani V.J., Vakharia D.P. (2011). Teaching–learning-based optimization: A novel method for constrained mechanical design optimization problems. Comput.-Aided Des..

[88] Liu Z.Z., Chu D.H., Song C., Xue X., Lu B.Y. (2016). Social learning optimization (SLO) algorithm paradigm and its application in QoS-aware cloud service composition. Inf. Sci..

[89] Shi Y. (2011). Brain storm optimization algorithm. Proceedings of the Advances in Swarm Intelligence: Second International Conference, ICSI 2011, Chongqing, China, 12–15 June 2011.

[90] Ghorbani N., Babaei E. (2014). Exchange market algorithm. Appl. Soft Comput..

[91] Moosavian N., Roodsari B.K. (2014). Soccer league competition algorithm: A novel meta-heuristic algorithm for optimal design of water distribution networks. Swarm Evol. Comput..

[92] Kumar M., Kulkarni A.J., Satapathy S.C. (2018). Socio evolution & learning optimization algorithm: A socio-inspired optimization methodology. Future Gener. Comput. Syst..

[93] Moghdani R., Salimifard K. (2018). Volleyball premier league algorithm. Appl. Soft Comput..

[94] Askari Q., Younas I., Saeed M. (2020). Political Optimizer: A novel socio-inspired meta-heuristic for global optimization. Knowl. Based Syst..

[95] Yang Y., Chen H., Heidari A.A., Gandomi A.H. (2021). Hunger games search: Visions, conception, implementation, deep analysis, perspectives, and towards performance shifts. Expert Syst. Appl..

[96] Zeidabadi F.A., Dehghani M. (2022). POA: Puzzle Optimization Algorithm. Int. J. Intell. Eng. Syst..

[97] Naruei I., Keynia F., Sabbagh Molahosseini A. (2022). Hunter–prey optimization: Algorithm and applications. Soft Comput..

[98] Rehman H., Sajid I., Sarwar A., Tariq M., Bakhsh F.I., Ahmad S., Mahmoud H.A., Aziz A. (2023). Driving training-based optimization (DTBO) for global maximum power point tracking for a photovoltaic system under partial shading condition. IET Renew. Power Gener..

[99] Ayyarao T.S., Ramakrishna N., Elavarasan R.M., Polumahanthi N., Rambabu M., Saini G., Khan B., Alatas B. (2022). War strategy optimization algorithm: A new effective metaheuristic algorithm for global optimization. IEEE Access.

[100] Faridmehr I., Nehdi M.L., Davoudkhani I.F., Poolad A. (2023). Mountaineering team-based optimization: A novel human-based metaheuristic algorithm. Mathematics.

[101] Pira E. (2023). City councils evolution: A socio-inspired metaheuristic optimization algorithm. J. Ambient. Intell. Humaniz. Comput..

[102] Javed S.T., Zafar K., Younas I. (2024). Kids Learning Optimizer: Social evolution and cognitive learning-based optimization algorithm. Neural Comput. Appl..

[103] Tian Z., Gai M. (2024). Football team training algorithm: A novel sport-inspired meta-heuristic optimization algorithm for global optimization. Expert Syst. Appl..

[104] Wu X., Li S., Jiang X., Zhou Y. (2024). Information acquisition optimizer: A new efficient algorithm for solving numerical and constrained engineering optimization problems. J. Supercomput..

[105] Oladejo S.O., Ekwe S.O., Mirjalili S. (2024). The Hiking Optimization Algorithm: A novel human-based metaheuristic approach. Knowl. Based Syst..

[106] Lang Y., Gao Y. (2025). Dream Optimization Algorithm (DOA): A novel metaheuristic optimization algorithm inspired by human dreams and its applications to real-world engineering problems. Comput. Methods Appl. Mech. Eng..

[107] Morales-Castañeda B., Zaldivar D., Cuevas E., Fausto F., Rodríguez A. (2020). A better balance in metaheuristic algorithms: Does it exist?. Swarm Evol. Comput..

[108] Yang X.S. (2020). Nature-inspired optimization algorithms: Challenges and open problems. J. Comput. Sci..

[109] Tang J., Liu G., Pan Q. (2021). A review on representative swarm intelligence algorithms for solving optimization problems: Applications and trends. IEEE/CAA J. Autom. Sin..

[110] Adam S.P., Alexandropoulos S.A.N., Pardalos P.M., Vrahatis M.N. (2019). No free lunch theorem: A review. Approximation and Optimization: Algorithms, Complexity and Applications.

[111] Lewis A., Smith D. (1993). Defining higher order thinking. Theory Into Pract..

[112] Hwang G.J., Lai C.L., Liang J.C., Chu H.C., Tsai C.C. (2018). A long-term experiment to investigate the relationships between high school students’ perceptions of mobile learning and peer interaction and higher-order thinking tendencies. Educ. Technol. Res. Dev..

[113] Lu K., Pang F., Shadiev R. (2021). Understanding the mediating effect of learning approach between learning factors and higher order thinking skills in collaborative inquiry-based learning. Educ. Technol. Res. Dev..

[114] Seddon G.M. (1978). The properties of Bloom’s taxonomy of educational objectives for the cognitive domain. Rev. Educ. Res..

[115] Hockly N. (2018). Blended learning. Elt J..

[116] Song Y., Cao J., Yang Y., Looi C.K. (2022). Mapping primary students’ mobile collaborative inquiry-based learning behaviours in science collaborative problem solving via learning analytics. Int. J. Educ. Res..

[117] Lee H.Y., Chen P.H., Wang W.S., Huang Y.M., Wu T.T. (2024). Empowering ChatGPT with guidance mechanism in blended learning: Effect of self-regulated learning, higher-order thinking skills, and knowledge construction. Int. J. Educ. Technol. High. Educ..

[118] Lu K., Yang H.H., Shi Y., Wang X. (2021). Examining the key influencing factors on college students’ higher-order thinking skills in the smart classroom environment. Int. J. Educ. Technol. High. Educ..

[119] Saido G.M., Siraj S., Nordin A.B.B., Al_Amedy O.S. (2018). Higher order thinking skills among secondary school students in science learning. MOJES Malays. Online J. Educ. Sci..

[120] Collins R. (2014). Skills for the 21st Century: Teaching higher-order thinking. Curric. Leadersh. J..

[121] Facione P.A. (1990). Critical Thinking: A Statement of Expert Consensus for Purposes of Educational Assessment and Instruction. Research Findings and Recommendations. ERIC Document Reproduction Service.

[122] Krathwohl D.R. (2002). A revision of Bloom’s taxonomy: An overview. Theory Into Pract..

[123] Suganthan P.N., Hansen N., Liang J.J., Deb K., Chen Y.P., Auger A., Tiwari S. (2005). Problem Definitions and Evaluation Criteria for the CEC 2005 Special Session on Real-Parameter Optimization.

[124] Tanabe R., Fukunaga A.S. (2014). Improving the search performance of SHADE using linear population size reduction. Proceedings of the 2014 IEEE Congress on Evolutionary Computation (CEC).

[125] Mohamed A.W., Hadi A.A., Fattouh A.M., Jambi K.M. (2017). LSHADE with semi-parameter adaptation hybrid with CMA-ES for solving CEC 2017 benchmark problems. Proceedings of the 2017 IEEE Congress on Evolutionary Computation (CEC).

[126] Awad N.H., Ali M.Z., Suganthan P.N. (2017). Ensemble sinusoidal differential covariance matrix adaptation with Euclidean neighborhood for solving CEC2017 benchmark problems. Proceedings of the 2017 IEEE Congress on Evolutionary Computation (CEC).

[127] Ahmed R., Rangaiah G.P., Mahadzir S., Mirjalili S., Hassan M.H., Kamel S. (2023). Memory, evolutionary operator, and local search based improved Grey Wolf Optimizer with linear population size reduction technique. Knowl. Based Syst..

[128] Li T., Shi J., Deng W., Hu Z. (2022). Pyramid particle swarm optimization with novel strategies of competition and cooperation. Appl. Soft Comput..

[129] Wu G., Mallipeddi R., Suganthan P.N. (2017). Problem Definitions and Evaluation Criteria for the CEC 2017 Competition on Constrained Real-Parameter Optimization.

[130] Mohamed A.W., Hadi A.A., Mohamed A.K., Awad N.H. (2020). Evaluating the performance of adaptive gainingsharing knowledge based algorithm on CEC 2020 benchmark problems. Proceedings of the 2020 IEEE Congress on Evolutionary Computation (CEC).

[131] Ahrari A., Elsayed S., Sarker R., Essam D., Coello C.A.C. Problem definition and evaluation criteria for the CEC’2022 competition on dynamic multimodal optimization. Proceedings of the IEEE World Congress on Computational Intelligence (IEEE WCCI 2022).

[132] Jia H., Lu C., Xing Z. (2024). Memory backtracking strategy: An evolutionary updating mechanism for meta-heuristic algorithms. Swarm Evol. Comput..

[133] Okoye K., Hosseini S. (2024). Wilcoxon Statistics in R: Signed-Rank Test and Rank-Sum Test. R Programming: Statistical Data Analysis in Research.

[134] Liang J.J., Qu B.Y., Suganthan P.N. (2013). Problem Definitions and Evaluation Criteria for the CEC 2014 Special Session and Competition on Single Objective Real-Parameter Numerical Optimization.

[135] Meng X.B., Li H.X., Gao X.Z. (2019). An adaptive reinforcement learning-based bat algorithm for structural design problems. Int. J. -Bio-Inspired Comput..

[136] Belegundu A.D., Arora J.S. (1985). A study of mathematical programming methods for structural optimization. Part I: Theory. Int. J. Numer. Methods Eng..

[137] Wadkar V., Malgave S., Patil D., Bhore H., Gavade P. (2015). Design and analysis of pressure vessel using ANSYS. J. Mech. Eng. Technol..

[138] Parsopoulos K.E., Vrahatis M.N. (2005). Unified particle swarm optimization for solving constrained engineering optimization problems. Proceedings of the International Conference on Natural Computation.

[139] Siddall J.N. (1982). Optimal Engineering Design: Principles and Applications.

[140] Kumar A., Wu G., Ali M.Z., Mallipeddi R., Suganthan P.N., Das S. (2020). A test-suite of non-convex constrained optimization problems from the real-world and some baseline results. Swarm Evol. Comput..

[141] Kumar A., Wu G., Ali M.Z., Mallipeddi R., Suganthan P.N., Das S. (2020). Guidelines for real-world single-objective constrained optimisation competition. Tech. Rep..

[142] Gupta S., Tiwari R., Nair S.B. (2007). Multi-objective design optimisation of rolling bearings using genetic algorithms. Mech. Mach. Theory.

[143] Arora J.S. (2004). Introduction to Optimum Design.

[144] Chew S.H., Zheng Q. (2012). Integral Global Optimization: Theory, Implementation and Applications.

[145] Grandhi R. (1993). Structural optimization with frequency constraints—A review. AIAA J..

[146] Deng L., Liu S. (2023). A multi-strategy improved slime mould algorithm for global optimization and engineering design problems. Comput. Methods Appl. Mech. Eng..

[147] Xu X., Xie C., Luo Z., Zhang C., Zhang T. (2024). A multi-objective evolutionary algorithm based on dimension exploration and discrepancy evolution for UAV path planning problem. Inf. Sci..

[148] Jiang Y., Xu X.X., Zheng M.Y., Zhan Z.H. (2024). Evolutionary computation for unmanned aerial vehicle path planning: A survey. Artif. Intell. Rev..

[149] Kaloev M., Krastev G. (2023). Comprehensive Review of Benefits from the Use of Sparse Updates Techniques in Reinforcement Learning: Experimental Simulations in Complex Action Space Environments. Proceedings of the 2023 4th International Conference on Communications, Information, Electronic and Energy Systems (CIEES), Plovdiv, Bulgaria.

[150] Dong Z., Zhang X., Zhang L., Giannelos S., Strbac G. (2024). Flexibility enhancement of urban energy systems through coordinated space heating aggregation of numerous buildings. Appl. Energy.

